# Twisted Allenyl-Pyridocyclophanes by Templated Cyclooligomerization: Chiral Cavities for Precision Molecular Recognition

**DOI:** 10.1021/jacsau.5c01555

**Published:** 2026-01-02

**Authors:** Jonathan Álvarez-García, María Magdalena Cid

**Affiliations:** Departamento de Química Orgánica. Ciencias Experimentais, 16784 Universidade de Vigo, Vigo 36310, Spain

**Keywords:** allenyl-cyclophanes, shape-persistent chiral cavities, host–guest complexes, template-cyclooligomerization, enantioselective molecular-recognition

## Abstract

We report the palladium-catalyzed one-pot synthesis of enantiopure 20-, 30-, and 40-membered cyclophanes shaped by axially twisted allene units. Under Cs^+^-template, the two smaller macrocycles (**2** and **3**) are favored over the largest (**4**). X-ray studies reveal that *rac*-**2** assembles into homochiral helices that pack to form channels, while *rac*-**3** forms racemic dimers. These shape-persistent hosts, with unique chiral 3D cavities, undergo guest-induced conformational switching and (enantio)­selectively bind diols and ammonium cations, including hydroxycarboxylic acids and α-hydroxyammoniums, as evidenced by diagnostic ECD shifts.

## Graphical abstract

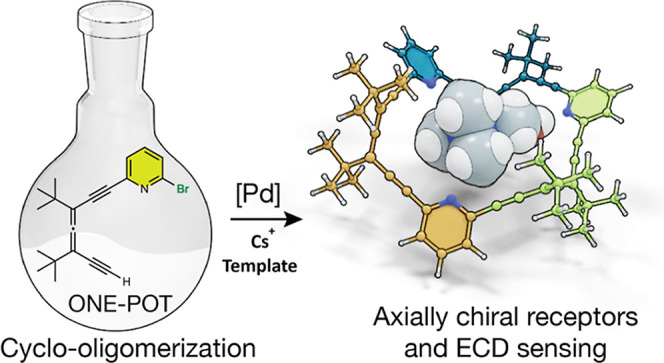

Chiral macrocycles are essential in chemistry, materials science, and pharmaceuticals thanks to their ability to form selective supramolecular assemblies and recognize specific guest molecules.^1^ In particular, twisted confined cavities achieve exceptional selectivity by balancing rigidity with adaptability (Scheme 1, top right).^2,3^ To generate such twisted chiral cavities, chiral axis, such as those found in allenes or biphenyls, have been employed.^4,5^ Their inherent 90° twist imparts a shape-persistent chiral topology, enabling the formation of structurally robust yet stereochemically responsive hosts (Scheme 1, top left).

**Scheme 1:**
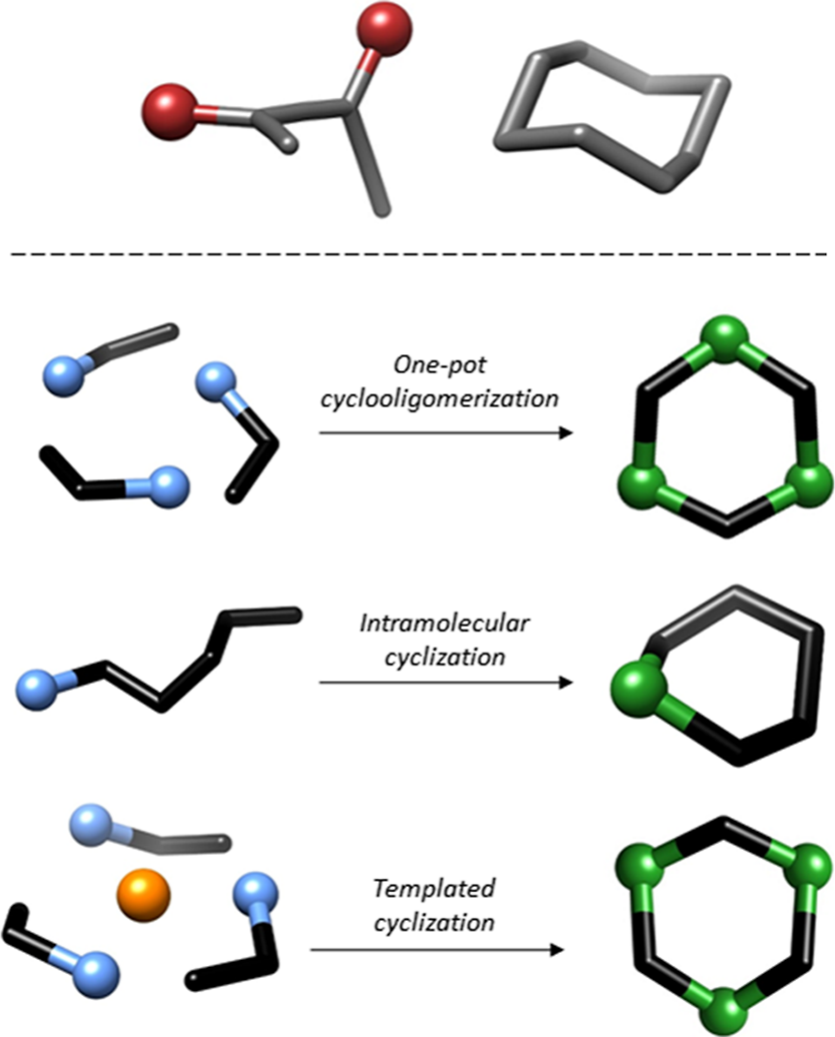
Top: Illustration of a Chiral Axis and a Chiral Twisted Cavity; Bottom: Some Strategies for the Synthesis of Macrocycles

Although macrocycle synthesis remains challenging,^6,7^ strategies such as one-pot cyclooligomerization or intramolecular closure provide efficient pathways (Scheme 1, bottom), typically relying on high dilution or preorganized precursors to counteract entropic penalties.^8–12^ Building on these design principles, our group has developed shape-persistent macrocycles containing 1,3-di-*tert*-butyl-1,3-diethynylallenes (**DEA**s),^5,13^ which confer rigidity and chirality to the backbone, along with pyridine rings to introduce functionality into the cavity. These chiral allenyl-cyclophanes, also termed **allenophanes**, display intense chiroptical signals that allowed the use of electronic circular dichroism (ECD) to fully characterize their conformational space and to monitor encapsulation processes.^14,15^

Initially, our strategy focused on maximizing chiroptical intensity by minimizing conformer diversity. More recently, we have shifted toward designing host molecules that adopt a single, optimal conformation upon guest binding. Although we have demonstrated the stepwise synthesis of several [14_*n*_]- and [7_*n*_]-allenophanes via final-stage Breslow oligodimerization or intramolecular Sonogashira macrocyclization,^16–18^ developing more concise and efficient routes remains a primary objective. Such advances would not only broaden access to these and other cyclic oligomers but also enable the synthesis of a plethora of structurally diverse molecules.

Addressing this challenge, we report here an enantioselective, one-pot synthesis of (*P*_2_)- and (*M*_2_)-[7_2_]-allenophane **2** via a palladium-catalyzed Sonogashira coupling, reducing both waste and reaction time compared to stepwise protocols.^19^ Applying this strategy, we synthesized two new allenophanes—(*P*_3_)- and (*M*_3_)-[7_3_]-allenophane **3**, and (*P*_4_)- and (*M*_4_)-[7_4_]-allenophane **4** (Scheme 2)—each featuring a shape-persistent, twisted 3D cavity that is otherwise challenging to construct. Theoretical calculations allowed us to map their conformational landscapes and assess their chiroptical performance. X-ray diffraction studies further enabled us to examine their chiral topologies in the solid state and to understand how they assemble into diverse supramolecular structures. Finally, leveraging the hydrogen-bond acceptor properties of the pyridine ring and the hydrophobic character of the **DEA** units, we demonstrate that the cavity shape and size of these allenophanes can be tuned to engage selectively with hydrogen-bond donor guests, thereby modulating the overall chiroptical response.

**Scheme 2:**
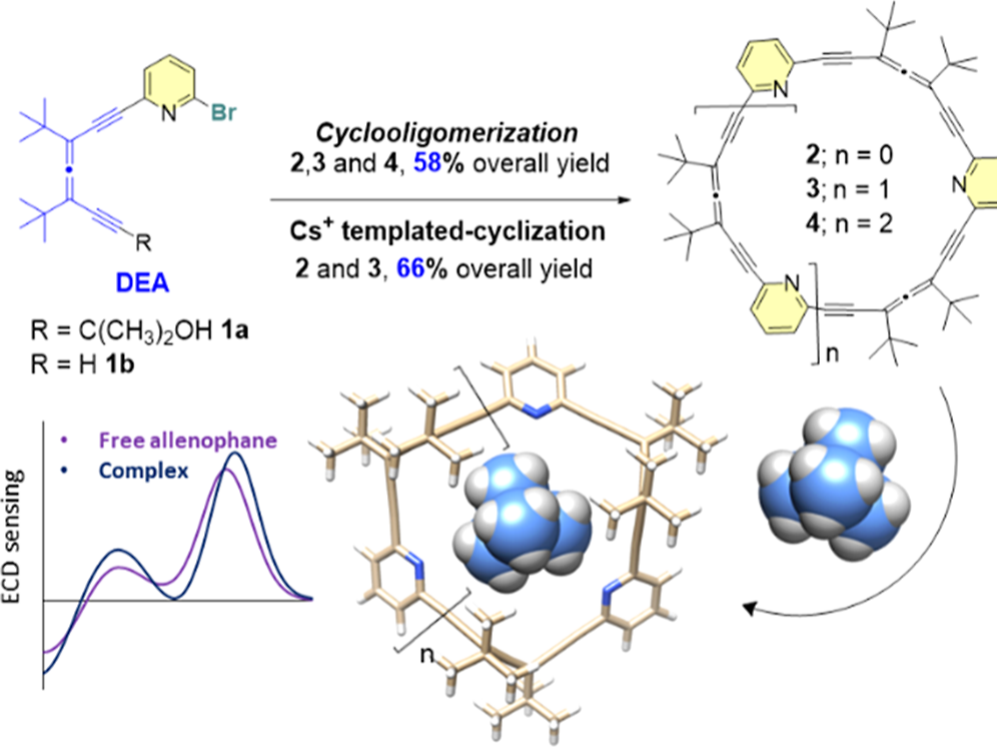
Pyrido-Allenophanes Presented in This Work and Chirality Sensing

## Results and Discussion

### Synthesis and Characterization of Allenophanes **2**, **3** and **4**

Previous computational studies revealed that the conformational space of [7_2_]-allenophane **2** is defined by a single conformer, which exhibits remarkable chiral efficiency, as evidenced by a dissymmetry (*g*)-factor^20^ of 0.006, an exceptional value for a small organic molecule.^19^ Consequently, efforts were directed toward developing a more efficient synthetic strategy. A cyclooligomerization of an allenyl-pyridyl monomer, such as **1**, would reduce the number of steps (Scheme 2) and, by adjusting reaction conditions, we anticipated the production of diverse allenophanes in a single batch.

Access to monomer **1b** required deprotection of **1a**.^19^ Based on our experience, the efficiency of alkyne-protecting-group removal is highly substrate-dependent. Among the explored bases for the deacetonation^21–23^ of monomer **1a**, excess NaOH in toluene at 110 °C gave the best results, yielding monomer **1b** in 97% yield (Table S1). As NaOH in toluene is compatible with palladium-catalyzed Sonogashira coupling, we explored the feasibility of oligomerizing (*P*)-**1a** with Pd­(PPh_3_)_4_ under the same conditions as in the deprotection reaction^24^ (Table 1, entry 1). Rewardingly, allenophane (*P*_2_)-**2** was formed along with two additional cyclooligomers, identified as new allenophanes with three ((*P*_3_)-**3**) and four ((*P*_4_)-**4**) monomer units, respectively (Table 1, entry 1). Mass spectrometry revealed that all the acyclic intermediates were completely consumed by 48 or 15 h, depending on whether (*P*)-**1a** or deprotected (*P*)-**1b** was used as the starting point (Table 1, entry 5). The reaction outcome was found to depend on the concentration of the monomer and on the temperature. The reaction using 5 mM of (*P*)-**1a** and 80 °C (Table 1, entry 4) gave an overall yield of 58%, which is remarkable considering the large number of steps involved (Table 1, entry 4). Allenophanes **2**, **3** and **4** were fully characterized and, as shown in their ^1^H NMR spectra, are highly symmetric (Figure S7b).

**Table 1:**
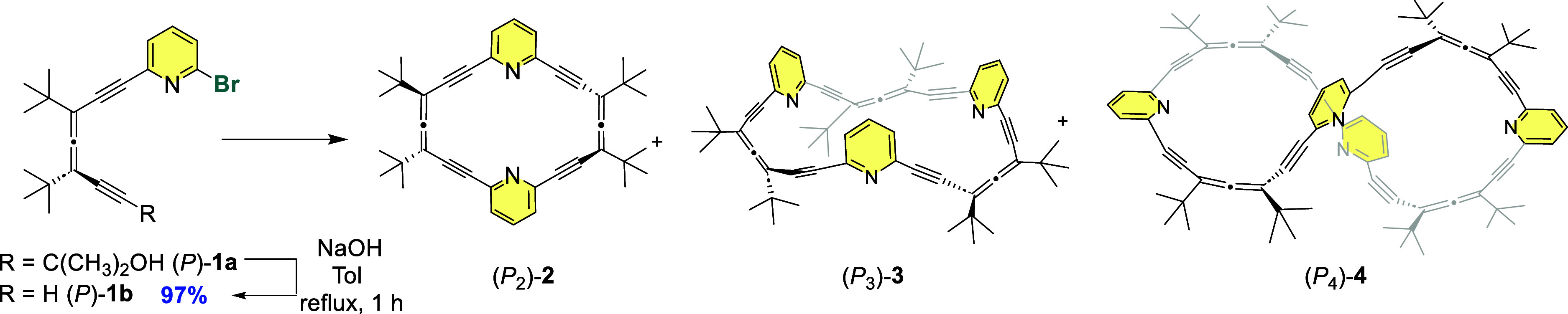
Synthetic Conditions for the Preparation of Allenophanes **2**, **3** and **4** ^d^

entry	solvent	base	1 [C]	*T* (°C)	time (h)	yield (%)	ratio **2**/**3**/**4**
1	toluene	NaOH	10.0 mM^a^	110	48	34	1/1.1/1.1
2	toluene	NaOH	5.0 mM^a^	110	48	49	1/1.05/0.8
3	toluene	NaOH	1.5 mM^a^	110	48	24	1/0.6/0
4	toluene	NaOH	5.0 mM^a^	80	48	58	1/0.85/0.8
5	toluene	NaOH	5.0 mM^b^	80	15	53	1/0.9/0.75
6	DMF	Cs_2_CO_3_ ^c^	5.0 mM^b^	80	18	57	1/0.36/0
7	DMF	Cs_2_CO_3_ ^c^	5.0 mM^b^	120	3	66	1/0.4/0
8	DMF	K_2_CO_3_ ^c^	5.0 mM^b^	120	8	38	1/0.55/0.55

a **1a** as starting material.

b **1b** as starting material.

c 6 equiv of base.

d General reaction conditions: monomer **1** was dissolved in the corresponding solvent together with 10% mol Pd­(PPh_3_)_4_ and base (1000 equiv) and heated at the indicated temperature and time under a N_2_ atmosphere.

Since the three obtained allenophanes, **2–4**, differ in the cavity size, template effects were investigated to assess selectivity in the cyclooligomerization process. Cesium cations (Cs^+^) are known for their pronounced templating effect in the synthesis of several heteroatom-containing macrocycles,^25–27^ particularly favoring the formation of 20 to 30 membered-rings.^28^ Considering that allenophanes (*P*_2_)-**2**, (*P*_3_)-**3** and (*P*_4_)-**4** have a 20-, 30-and 40-membered rings, respectively, one can reasonably anticipate selective formation of the macrocycles. To harness the cesium effect, we used Cs_2_CO_3_ as the base and DMF as the solvent, that significantly boosted the amount of (*P*_2_)-**2** relative to (*P*_3_)-**3**, at the expense of (*P*_4_)-**4** (Table 1, entry 6). Further increasing the temperature to 120 °C shortened the reaction time to just 3 h and increased the overall yield to 66%, with a product ratio of 1:0.4:0 for allenophanes (*P*_2_)-**2**/(*P*_3_)-**3**/(*P*_4_)-**4** (Table 1, entry 7). The templating effect of cesium was confirmed by ESI-HR, revealing the presence of [**2**·Cs]^+^ (*m*/*z* = 683.2397) and [**3**·Cs]^+^ (*m*/*z* = 958.4069) complexes in the reaction mixture (Figure S7a). In addition, when Cs_2_CO_3_ was replaced by K_2_CO_3_ (Table 1, entry 8), the overall yield dropped, and the largest macrocycle (*P*_4_)-**4** was again formed.

Accordingly, we have successfully developed an efficient one-pot methodology for the preparation of bis- and tris­(allenyl-pyrido) macrocyclophanes, affording the desired products in good overall yields. The results were consistent with both *M*- or (±)-**1**. We anticipate that the same strategy can be readily extended to the synthesis of other related systems.

### X-ray Studies

Slow evaporation of a solution of (*M*_2_)-**2** in Et_3_N yielded single crystals in the *P*3_1_ space group,^19^ with the allenophane as the asymmetric unit and showing a 6.03 Å separation between the pyridyl nitrogen atoms (Figure 1a). (*M*_2_)-**2** molecules arrange themselves into helical columns with *P*-helicity along the *c*-axis —opposite to the configuration of the monomer—featuring a helical pitch of 14.96 Å and a width of 17.02 Å (Figure 1b,c). The primary noncovalent interactions stabilizing this packing are CH···N interactions,^29^ occurring between the hydrogen atom at the 4-position of a pyridine ring and the nitrogen atom of the adjacent macrocycle, with an average distance of 2.44 Å. Furthermore, this packing generates elongated helical cavities (diameter ≈ 5.6 Å) running along the *c*-axis of the crystal, forming continuous channels throughout the crystal lattice (Figure 1d).

**Figure 1:**
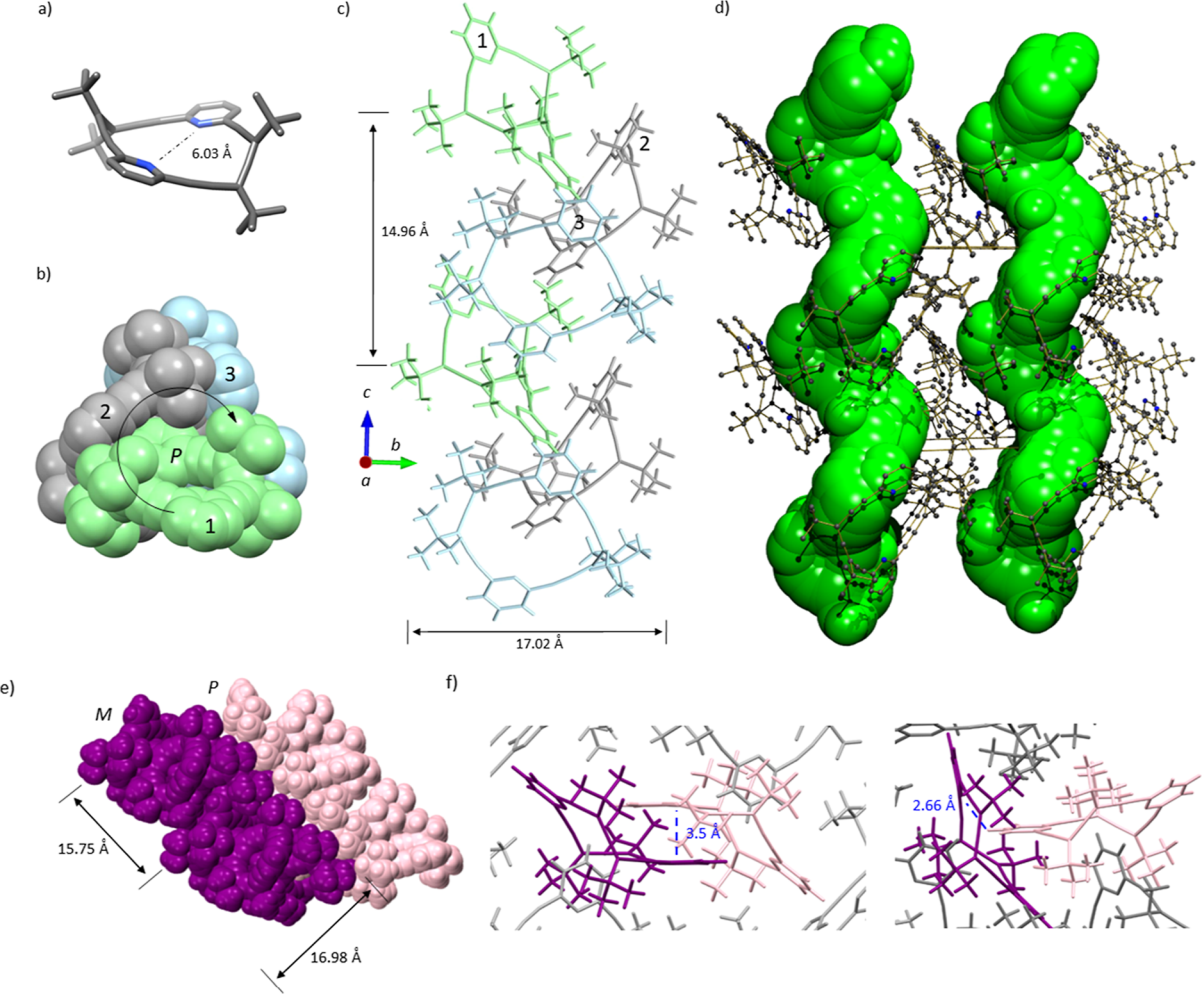
X-ray diffraction structures. (a) Molecular structure of (*M*_2_)-**2**; (b,c) supramolecular helical packing of (*M*_2_)-**2** along the *c*-axis; (d) continuous helical cavities along the *c*-axis (diameter ≈ 5.6 Å); (e) packing mode of the (*M*_2_)/(*P*_2_)-**2** racemate: *M*-helices (purple, composed of (*P*_2_)-**2**) and *P*-helices (pink, composed of (*M*_2_)-**2**) (f) close-up view of the intermolecular interactions between (*M*_2_)-**2** (pink) and (*P*_2_)-**2** (purple) macrocycles.

Crystallization of the racemate (*M*_2_)/(*P*_2_)-**2** by slow concentration of a stereoisomeric mixture of **2** in heptane/methyl *tert*-butyl ether (MTBE) produced crystals in the P2_1_/n space group with three homochiral macrocycles in the asymmetric unit. The racemic system exhibits a narcissistic self-sorting phenomenon,^30,31^ in which (*M*_2_)-**2** selectively assembles with itself to form *P*-helical columns, while (*P*_2_)-**2** assembles into *M*-helices (Figure 1e, CCDC 2454364). In contrast to the enantiopure crystal—where helices interact only through van der Waals forces—the racemic helices engage in strong interhelix interactions. These include both CH···N hydrogen bonds (2.66 Å) and π–π stacking interactions^32^ (3.5 Å), between *M* and *P* macrocycles (Figure 1f). This close packing induces a distortion in the helical architecture, leading to an increase in helical pitch (15.75 Å) compared to the enantiopure counterpart. Moreover, the interlocking of the helices eliminates the continuous channels observed in the enantiopure structure. Instead, the racemic crystal features smaller, isolated solvent-filled pores, occupied by heptane molecules.

Overall, the enantiopure and racemic forms of (*M*_2_)/(*P*_2_)-**2** exhibit distinct packing behaviors, leading to either porous channel-like structures in the enantiopure crystal or compact, interlocked architectures in the racemate.

On the other hand, the stereoisomeric mixture of allenophane **3**, obtained from (±)-**1**, afforded crystals from MTBE in the form of a racemate of the homochiral (*P*_3_)-**3** and (*M*_3_)-**3** that were suitable for X-ray diffraction. The crystallographic data present **3** adopting a crown-shaped conformation, with the pyridyl nitrogen atoms positioned equidistantly at 8.36 Å from one another (Figure 2a, CCDC 2365576). Supramolecular racemate dimers of **3** are formed through the establishment of nonclassical CH···N hydrogen bonding interactions, where methyl groups interact with pyridine rings of neighboring allenophanes. Since enantiopure (*P*_3_)-**3** or (*M*_3_)-**3** did not produce suitable crystals for X-ray diffraction, the social self-sorting^33^ exhibited by the dimers in the racemate seems to be crucial for successful crystallization, as the same interactions cannot be established between enantiopure stereoisomers. Moreover, each of these dimers establishes CH···π interactions with six other dimers, three on the upper face and three on the lower face, creating a three-dimensional network reminiscent of a honeycomb pattern (Figure 2b).

**Figure 2:**
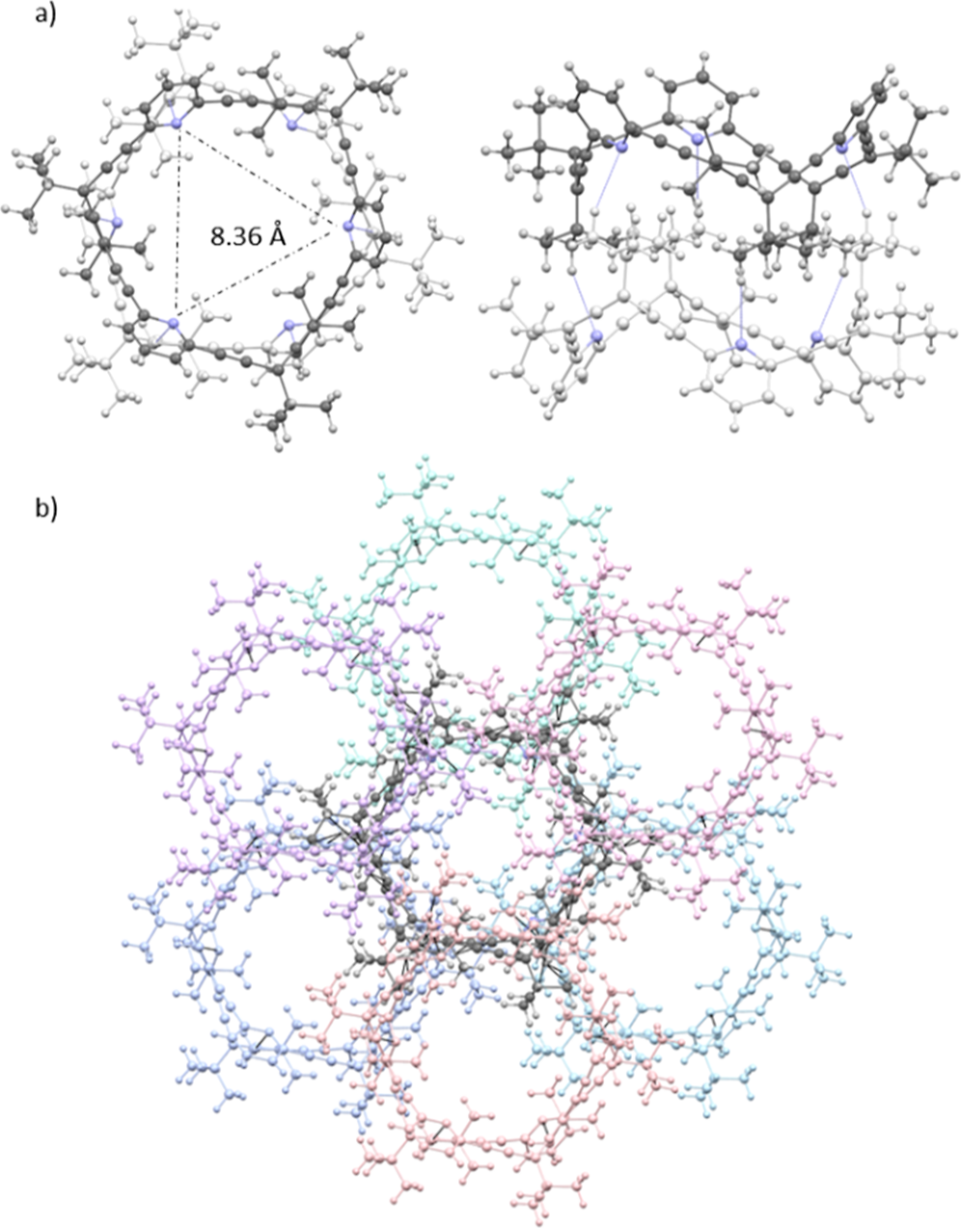
X-ray diffraction structure for (a) dimer of *rac*-(*P*_3_)/(*M*_3_)-**3**; (b) 3D-distribution composed of seven dimers of *rac*-(*P*_3_)/(*M*_3_)-**3**. The central dimer is depicted in dark gray, with three dimers in shades of pink above and three dimers in shades of blue below.

These results highlight the inherent structural features of allenophanes as powerful drivers of supramolecular organization, capable of directing the formation of diverse packing motifs—from helical porous channels to compact 3D networks—through well-defined noncovalent interactions.

### Chiroptical Characterization of Allenophanes **2**, **3** and **4**

To investigate the effect of cyclooligomerization of (*P*)-**1** into macrocyclic systems, the chiroptical properties of allenophanes **2**, **3**, and **4** were examined. (*P*_2_)-**2** displays an ECD spectrum in acetonitrile with two negative Cotton effects at 302 and 320 nm, and a strong positive one at 261 nm, with a *g*-factor of 0.006 ^19^ (Figure 3a, blue line). (*P*_3_)-**3** exhibits the same sign pattern of Cotton effects (−/−//+), from lower to higher energy (Figure 3b, blue line), also with a *g*-factor of 0.006, despite featuring a more flexible cavity. Finally, the ECD spectrum of (*P*_4_)-**4** shows a (−/+/+) pattern centered at 348, 319, and 279 nm (Figure 3c, blue line), with a *g*-factor of 0.002. The ECD spectra of the enantiomers (*M*, (–/+*M*_3_)-**3** and (*M*_4_)-**4** are mirror images of those of the corresponding *P* enantiomers (Figure 3a–c, green lines).

**Figure 3:**
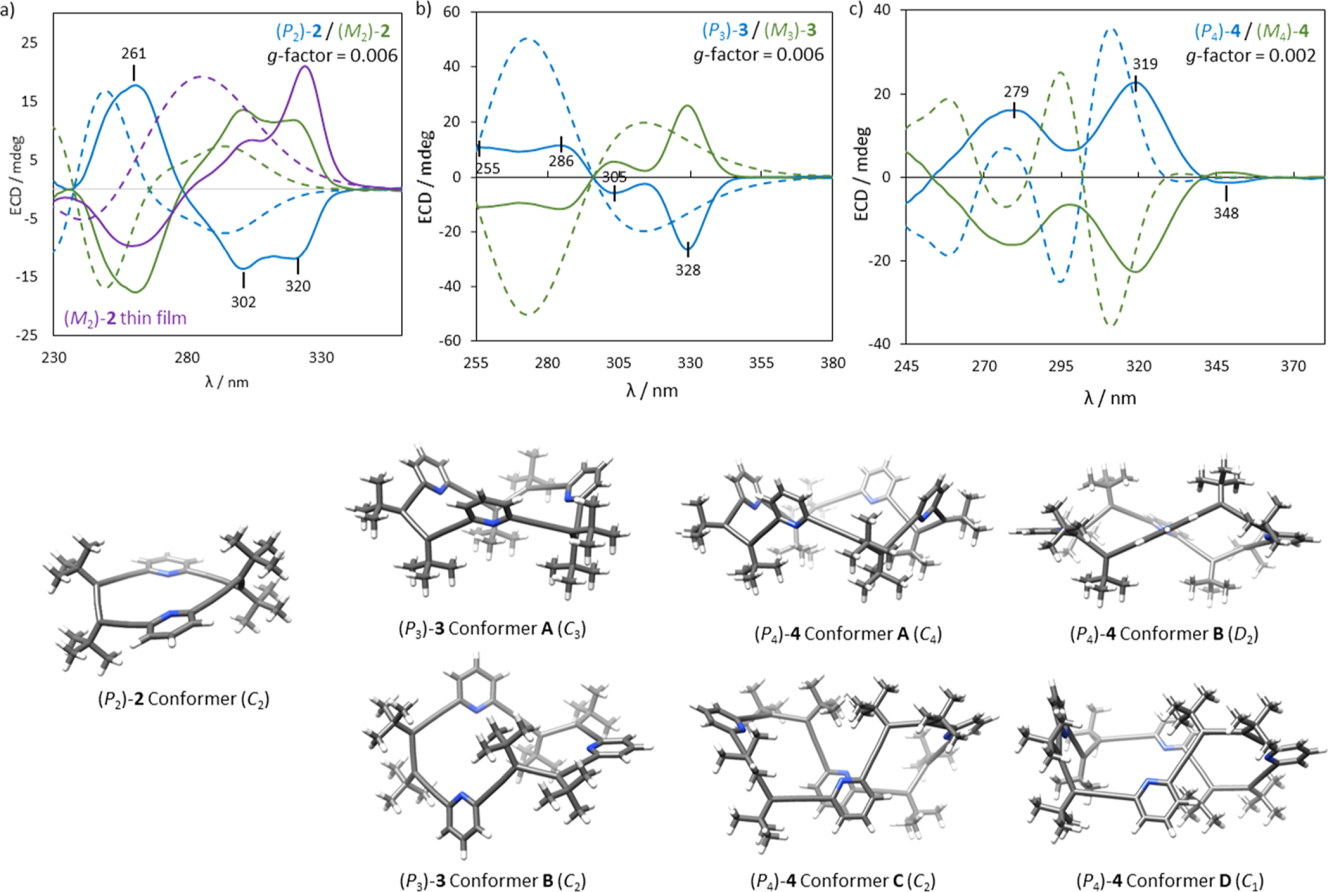
ECD spectra for homochiral enantiomeric pair of (a) **2** (CH_3_CN, 1.5 × 10^–5^ M), (b) **3** (CH_3_CN, 1.5 × 10^–5^ M) and (c) **4** (CH_3_CN, 4 × 10^–6^ M). Experimental ECD in solid lines and calculated ECD in dashed lines (TDDFT CAMB3LYP/6-31G+(d,p), smd = acetonitrile). Conformers identified for each allenophane (DFT CAMB3LYP/6-31G+(d,p)). In (a), the ECD spectrum of solid-state (*M*_2_)-**2** is also included in purple.

Since circular dichroism efficiency is sensitive to individual geometries and, hence, to the conformational space population,^34,35^ DFT calculations were performed at the CAM-B3LYP/6-31G+(d,p)^36^ level to identify the predominant conformers of each allenophane in solution. (*P*_2_)-**2** populates a single conformer of *C*_2_-symmetry whose geometry coincides with the X-ray structure (Figures 1a vs [Fig fig3]); for (*P*_3_)-**3**, there are two conformers with nearly equal energy, with *C*_3_- and *C*_2_-symmetry (Figure 3). While for (*P*_4_)-**4**, four distinct conformers were identified with similar energy, displaying *C*_4_-, *C*_2_-, *C*_1_- and *D*_2_-symmetry (Figure 3). The greater conformational plasticity of (*P*_4_)-**4** explains its lower *g*-factor. The signs of the main ECD bands of allenophanes **2**–**4** were successfully reproduced by theoretical calculations (Figure 3a–c, solid vs dashed lines).

In addition, given that enantiopure allenophane (*M*_2_)-**2** forms helical assemblies in the solid state, its ECD spectrum was also measured in the solid phase.^37^ To this end, a solution of (*M*_2_)-**2** in Et_3_N was allowed to slowly evaporate on a quartz plate (see Supporting Information for details). Notably, the intensity of the band at 320 nm was significantly enhanced relative to the other bands (Figure 3a, purple line). To investigate whether this effect arises from supramolecular structures similar to those observed in the crystal state, the ECD spectrum of the X-ray-based supramolecular assembly was computed (Figure 3a, purple dashed line, and Figures S66 and S67). The calculated spectrum closely reproduced the experimental one, including the hyperchromic effect of the 320 nm band compared to the spectrum of the isolated allenophane.

These results underscore the pivotal role of macrocyclic topology and supramolecular organization in shaping the chiroptical signatures of allenophanes.

### Host–Guest Chemistry

With the structure and chiroptical properties of enantiopure allenophanes **2** and **3** established, their molecular recognition potential was investigated, leveraging their distinct twisted topologies.

### Allenophane **2**

Inspired by the orientation of the lone pairs of the nitrogens, we envisioned allenophane **2** as a tool for sensing small organic molecules with appropriately positioned hydrogen bond donors, that would enable bidentate binding (Figure 4).

**Figure 4:**
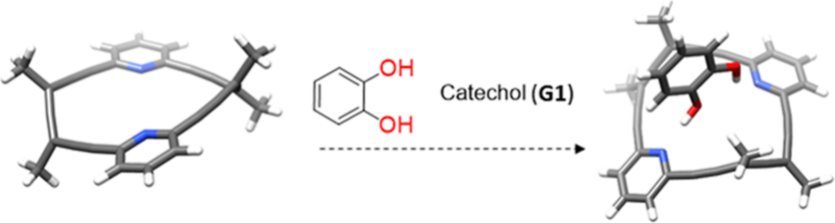
Computational model of (*P*_2_)-**2**, left; (*P*_2_)-**2** and catechol (**G1**), right. The *tert*-butyl groups were replaced by methyl groups (DFT CAM-B3LYP 6-31-G+(d,p)).

Catechol (**G1**), featuring ortho hydroxyl groups, was selected as a model substrate due to its role as a common structural motif in many biologically relevant molecules, including drugs and neurotransmitters.^38^ In addition, catechoĺs persistence, low biodegradability, and toxicity to both humans and aquatic life^39^ make its detection and quantification particularly important. Although numerous analytical methods have been reported for catechol detection, among them electrochemical sensing,^40^ there remains a clear need to develop selective protocols to distinguish the different isomers of dihydroxybenzene.^41^

Preliminary computational studies indicated that catechol fits into the macrocycle’s cavity forming hydrogen bonds with both pyridine rings (Figure 4). Complexation was studied in chloroform by ^1^H NMR spectroscopy (Figure 5a).

**Figure 5:**
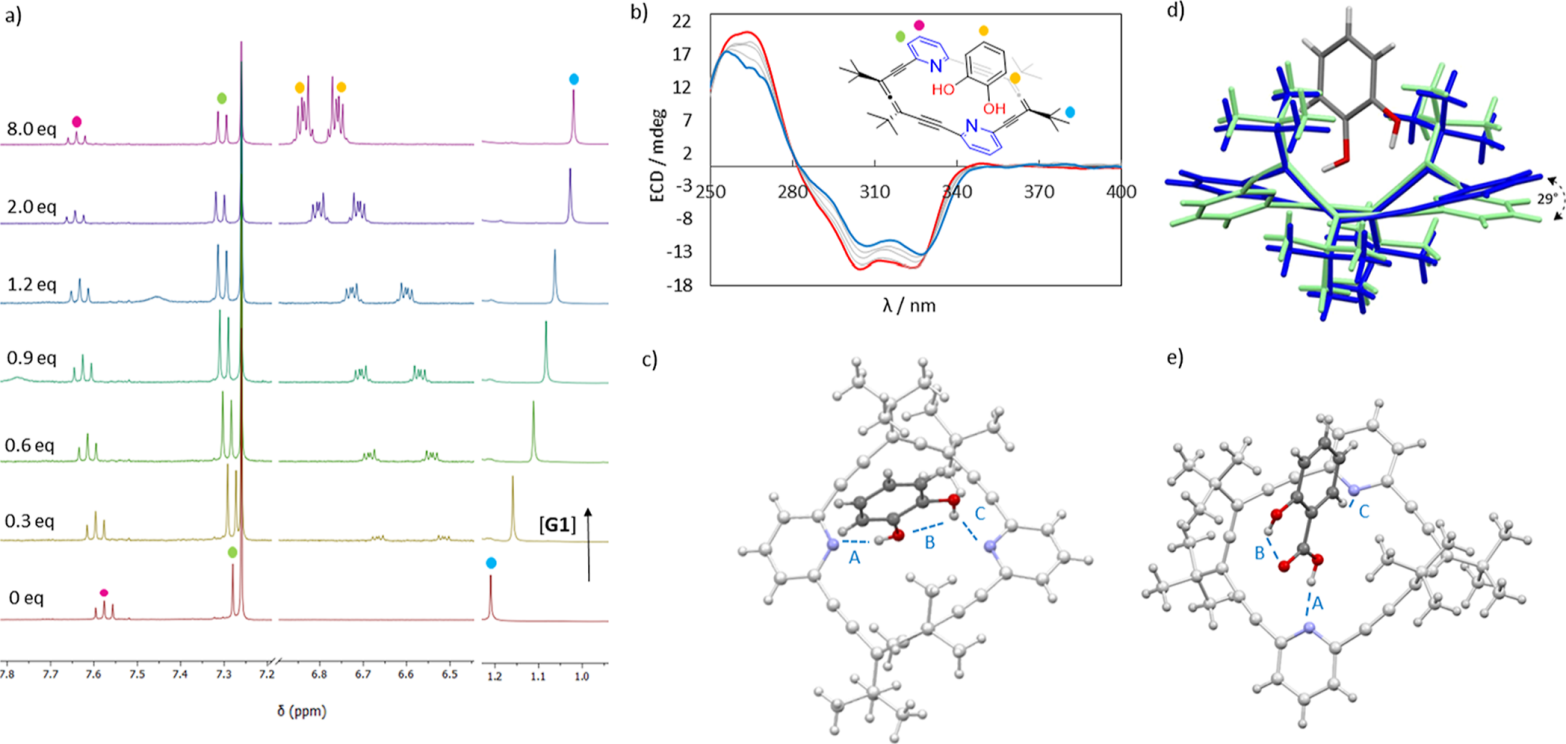
(a) ^1^H NMR titration of (*P*_2_)-**2** (0.018 M) with catechol (**G1**) in CDCl_3_; dots correspond to py-H as marked in (b). (b) ECD titration of (*P*_2_)-**2** with catechol **G1**. Original spectrum (red line, CHCl_3,_ 1.5 × 10^–5^ M) and end point of titration (blue line). (c) Crystal structure of complex [(*rac*)-**2**·**G1**]. Dashed lines denote hydrogen bonds [Å]: A 1.92, B 2.15, and C 2.29. (d) Comparison between the crystal structures obtained by X-ray diffraction of unbound allenophane **2** (dark blue) and bound to catechol (green). (e) Crystal structure of the 1:1 complex [(*rac*)-**2**·**G5**]. Dashed lines denote hydrogen bonds [Å]: A 1.79, B 1.83, and C 2.51.

As the concentration of catechol (**G1**) increased, the pyridine signals were gradually deshielded, consistent with intermolecular hydrogen bonding. Simultaneously, *tert*-butyl signals underwent a more significant upfield shift, indicating shielding by the catechol ring and inclusion complex formation. Titration data fit a 1:1 binding model using BindFit software,^42,43^ resulting in an association constant of *K*_1_ = (321 ± 44) M^–1^.

The negative Cotton effects at 305 and 324 nm in the ECD spectrum (Figure 5b, red line) became slightly more positive and shifted to 307 and 329 nm, respectively, as the catechol concentration increased (Figure 5b, blue line). Isodichroic points at 280 and 329 nm support the formation of a 1:1 complex. To our knowledge, this is the first chiroptical (ECD) detection of an achiral catechol ring. Single crystals of the host–guest complex racemate, obtained by slow evaporation of a stereoisomeric mixture of 2 from a heptane/MTBE/methanol solution, revealed that catechol (**G1**, Table 2) inserts vertically into the host cavity. It forms hydrogen bonds between its two phenol groups and the pyridine nitrogen atoms of the macrocycle, with OH···N distances of 1.92 and 2.29 Å. Additionally, catechol retains an intramolecular hydrogen bond between its hydroxyl groups, while CH···π interactions^44^ are stablished between the *tert*-butyl groups of the allenophane and the aromatic ring of the guest (Figure 5c, CCDC 2365579). Comparing the crystal structure of free allenophane **2** with its complex with catechol, pyridine rings tilt by 29° relative to their orientation in the unbound form to facilitate hydrogen bonding with the phenolic groups (Figure 5d).

**Table 2:**
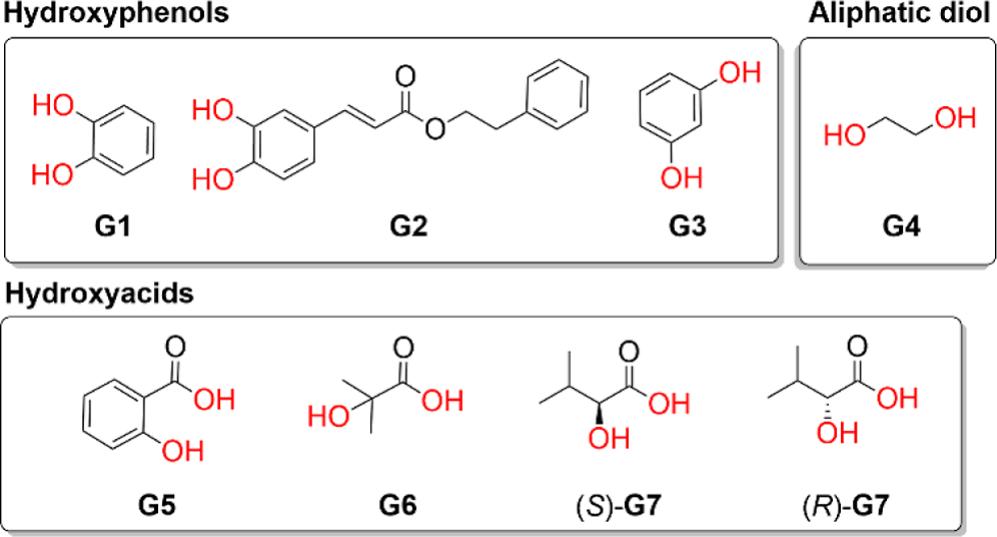
Structures of Tested Guests **G1–G7** for Allenophane 2 and Corresponding Association Constants

entry	guest	(H/G)	*K* (M^–1^)
1	**G1** ^a^	1:1	*K*_1_ = 321 ± 44
3	**G2** ^b^	1:1	*K*_1_ = 122.6 ± 9.8
4	**G3** ^b^	1:2	*K*_11_ = 4.5 ± 0.2, *K*_12_ = 1.20 ± 0.09
5	**G4** ^a^	-	-
6	**G5** ^a^	1:2	*K*_11_ = 100.4 ± 6.9, *K*_12_ = 1.22 ± 0.09
7	**G6** ^a^	1:1	*K*_1_ = 120.2 ± 12.0
8	(*S*)-**G7^c^** ^a^	1:1	*K*_1_ = 12.9 ± 0.7^c^
9	(*R*)-**G7^c^** ^a^	1:1	*K*_1_ = 27.3 ± 1.7^c^

a CHCl_3_ as solvent, NMR-titration.

b CHCl_3_/MeOH 5% as solvent. NMR-titration. Methanol was added to solve solubility issues but used sparingly to avoid interfering with the complexation process.

c Constants calculated from ECD-titration.

To extract thermodynamic parameters and gain deeper insight into the binding process, the association constant of the catechol complex was determined at different temperatures by ECD (Figures S69 and S71). Notably, the association constants obtained by ECD (*K*_a_ = 1261 M^–1^ at 25 °C, Table S7) are higher than those determined by NMR (*K*_a_ = 321 M^–1^ at 25 °C), likely reflecting differences in sensitivity and concentration-dependent effects: NMR averages all species in fast exchange, while ECD primarily detects the most strongly bound, chiral complex.^45^ The thermodynamic analysis (Table S8, Δ*H* ≈ −16 kJ·mol^–1^, Δ*S* = +7.9 J·mol^–1^ K^–1^, Δ*G* ≈ −18 kJ·mol^–1^ at 298 K) indicates that the complexation is enthalpy-driven, consistent with the formation of hydrogen bonds within a preorganized host. These results agree well with the X-ray and spectroscopic data, confirming a hydrogen-bond-driven inclusion within a semiflexible cavity.

In addition to catechol, allenophane **2** binds a range of structurally diverse biologically compounds containing two hydrogen bond donors, such as either two hydroxyl groups or a hydroxyl group paired with a carboxylic acid (Table 2).

This binding was demonstrated by several complexation experiments, primarily monitored by ^1^H NMR spectroscopy (Figures S17–S31). Caffeic acid phenethyl ester (CAPE, **G2**), a bioactive natural product,^43,44^ formed a 1:1 complex when a small amount of methanol was added to chloroform to solve solubility issues (Table 2, entry 2).

To investigate the significance of the relative positioning of the hydrogen-donating groups, additional systems were examined. In the binding study of resorcinol (**G3**), a regioisomer of catechol, the meta-positioning of hydroxyl groups led to a 1:2 stoichiometry, with two resorcinol molecules binding to allenophane **2** (Figure S56). Resorcinol’s reduced ability to form two hydrogen bonds with the host allowed a second molecule to bind (Table 2, entry 4). The binding was noncooperative as indicated by the interaction parameter α ^45,46^ (α ≈ 1, where α = 4*K*_12_/*K*_11_), suggesting that the binding of the first resorcinol molecule does not influence the binding of the second.

Ethylene glycol (**G4**), with two aliphatic hydroxyl groups, did not show any complexation with allenophane **2** in ^1^H NMR studies, even at high concentrations (up to 1.5 M). We attribute the lack of binding to the lower acidity of aliphatic hydroxyls, the antiorientation of the O–H bonds in **G4́**s more stable conformation, and the absence of an aromatic ring—unlike the successful complexation seen with aromatic compounds like catechol, CAPE, and, to a lesser extent, resorcinol. Notable, the superior binding of catechol highlights the selectivity of this pyridoallenophane toward *ortho*-dihydroxybenzenes, underscoring its potential as selective receptor for catechol derivatives in the presence of other structurally related isomers within complex matrices.

α-Hydroxy acids such as salicylic acid (**G5**), 2-hydroxyisobutyric acid (**G6**), and 2-hydroxy-3-methylbutyric acid (**G7**) can form salt bridges and hydrogen bonds with the pyridine rings, making them promising candidates for strong inclusion complexes (Table 2, entries 6–9). Surprisingly, salicylic acid (**G5**) formed a 1:2 complex despite the *ortho* positioning of its hydroxyl and carboxylic acid groups. The interaction parameter α (α = 0.05) indicated a strong negative cooperativity—binding the first guest prevents binding of a second—an uncommon phenomenon in synthetic receptors.^47,48^

The crystal structure of [(*rac*)-**2**·**G5**], obtained from a heptane/MTBE/methanol mixture, presents salicylic acid positioned vertically within the host. Its carboxyl group forms a hydrogen bond (*d*_(OH···N)_ = 1.79 Å) with one pyridine nitrogen, rather than a salt bridge, while the hydroxyl group engages in intramolecular hydrogen bonding with the neighboring carboxyl group, precluding interacting with the second pyridine. A nonclassical CH···N hydrogen bond (2.51 Å) is also formed between the CH group *ortho* to the carboxylic acid in **G5** and the second pyridine nitrogen (Figure 5e, CCDC 2365580), helping to explain the negative cooperativity by electronically hindering a second binding event.

In contrast, the aliphatic hydroxyacid, 2-hydroxyisobutyric acid (**G6**) formed the expected 1:1 complex with allenophane **2** (Table 2, entry 7). In this case, the host establishes two hydrogen bonds with both the carboxylic and the hydroxy groups of the guest, favoring inclusion (Figure S57).

Given the intrinsic chirality of **2**, we then explored its potential for enantiomeric discrimination using the human metabolite 2-hydroxyisovaleric acid (**G7**). Titration studies monitored by ECD revealed enantioselective binding, with a clear preference for the (*R*)-**G7** enantiomer (*K*_*R*_/*K*_*S*_ = 2.1) (Table 2, entry 8 and 9). Computational models suggest that (*R*)-**G7** fits more favorably into the allenophane cavity, stabilizing (*P*_2_)-**2**·(*R*)-**G7** complex over its (S) counterpart (Figure S58 and S59). The observed enantiodiscrimination likely arises from steric effects, as the isopropyl side chain of (*S*)-**G7** is positioned closer to the bulky *tert*-butyl groups of host (*P*_2_)-**2**.

### Allenophane **3**

In the *C*_3_-symmetric conformer of allenophane **3**, the three pyridine units are oriented inward, toward the central cavity, suggesting that *C*_3_-symmetric, suitably sized cations could fit well and form stable complexes. The objective of this study was to explore the potential of **3** to bind (chiral)­ammonium cations, which are biologically relevant and frequently targeted by synthetic receptors.^49–51^ To evaluate its binding capabilities, a series of ammonium salts (**G8**–**G15**) with varying aliphatic chain lengths and degrees of branching were selected (Figure 6a). Additionally, α-hydroxy-ammoniums salts such as choline^52–54^ and an l-carnitine^55^ derivative (**G16**–**G17**)—both essential human nutrients—were included as potential guests.

**Figure 6:**
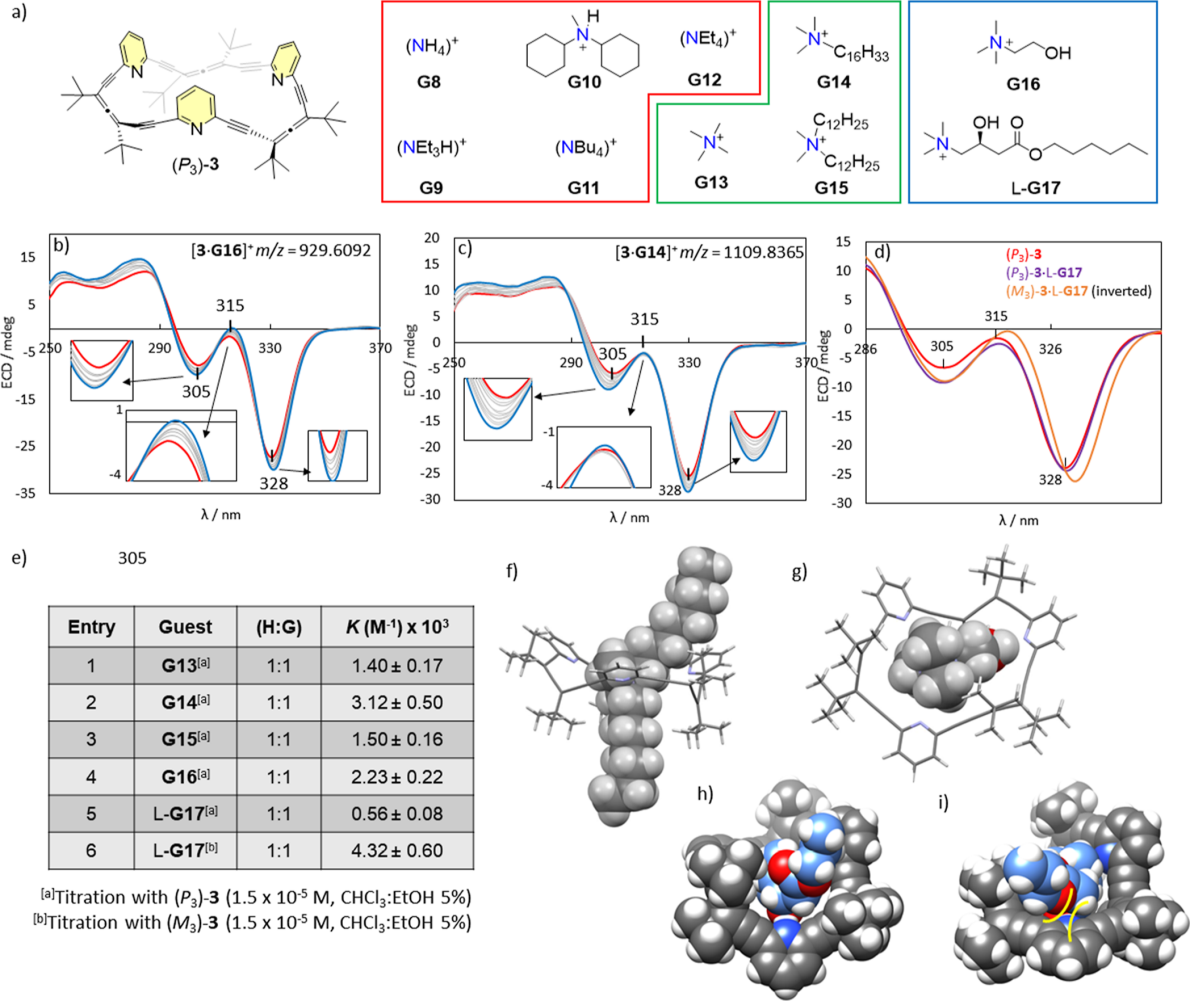
(a) Structures of the ammoniums tested as potential guests for allenophane **3**, with counterions as follows: 2-hydroxyisobutyrate for **G10** and **G16**, chloride for **G8**–**G9** and **G11**–**G15**, and iodide for L-**G17**. (b,c) ECD titrations of (*P*_3_)-**3** (1.5 × 10^–5^ M, CHCl_3_/EtOH 5%) with guests **G14** and **G16**, respectively. The original spectra (red lines) and final spectra at saturation (blue lines) show a notable difference near 315 nm (highlighted with a green circle). (d) ECD spectra of free (*P*_3_)-**3** (red), its complex with L-**G17** (purple), and the inverted spectrum of the complex with (*M*_3_)-**3**·L-**G17** (orange). (i) Table summarizing the association constants (*K*) between allenophane **3** and each guest. (f–i) DFT-optimized computational models (WB97XD or CAMB3LYP, 6-31+G­(d,p)) of the inclusion complexes: [(*P*_3_)-**3**·**G15**]^+^, [(*P*_3_)-**3**·**G16**]^+^, [(*P*_3_)-**3**·L-**G17**]+, and [(*P*_3_)-**3**·D-**G17**]^+^.

Binding interactions were monitored by ECD spectroscopy in chloroform with a minimal amount of ethanol added to mitigate solubility issues. High-resolution mass spectrometry further confirmed host–guest complex formation (Figures S34–S48).

Ammonium salts **G8**–**G12** showed no noticeable changes in the original ECD spectrum of (*P*_3_)-**3**, even at concentrations exceeding 0.3 M, suggesting no detectable complexation under the experimental conditions. In contrast, salts **G13**-**G15** induced consistent and measurable alterations in the ECD spectra. These results demonstrated that the size and steric bulk of the ammonium cation’s R groups play a key role for effective binding. Ammonium ions that were too small (**G8**), overly large (**G9**, **G11**–**G12**) or sterically hindered (**G10**) were unable to fit into the host́'s cavity. However, tetraalkylammonium salts **G13**–**G15**—with at least two methyl groups—formed stable complexes. Figure 6b illustrates these spectral changes using cetrimonium chloride (**G14**) as a representative example. Increasing concentrations of **G14** caused hyperchromic shifts at 305 and 328 nm (red vs blue line), and the presence of three isodichroic points at 290, 310, and 319 nm supports a 1:1 binding equilibrium between (*P*_3_)-**3** and **G14**. Fitting the data to a 1:1 binding model using BindFit software yielded association constants on the order of 10^3^ M^–1^ (Figure 6e, entries 1–3).

Encouragingly, increasing choline (**G16**) concentrations induced distinct changes in the ECD spectrum of allenophane (*P*_3_)-**3**, indicating strong host–guest interactions (Figure 6c, red line). The original bands intensified, with slight blue shifts at 284 and 305 nm and red shifts at 315 and 328 nm (Figure 6c, blue line). These spectral features differed notably from those seen with **G13**–**G15**, particularly around 315 nm (Figure 6b). Isodichroic points at 290, 310, and 327 nm, along with data fitting, supported a well-defined 1:1 binding model, yielding an association constant of 2.23 × 10^3^ M^–1^ (Figure 6e, entry 4). In contrast to allenophane **2**, complexation in this case is entropy-driven (Table S8, Δ*H* ≈ −3 kJ·mol^–1^, Δ*S* = +60 J·mol^–1^ K^–1^, Δ*G* ≈ −20 kJ·mol^–1^ at 298 K) as indicated by the thermodynamic parameters determined from variable temperatures studies with **G16** (Table S7, Figure S70 and S72), suggesting that desolvation and the release of ordered solvent molecules play a key role in the stabilization of the complex.

Next, enantioselective sensing ability of allenophane **3** was evaluated using its homochiral enantiomers, (*P*_3_)-**3** and (*M*_3_)-**3**, against *l* -carnitine hexyl ester (L-**G17**). The resulting ECD spectra (Figure 6d) were similar to those with choline but showed distinct enantiodifferentiation. The band at 305 nm exhibited a hyperchromic shift, while the 315 nm band shifted batochromically—becoming more positive for (*M*_3_)-**3**·L-**G17** (Figure 6d, orange line) and more negative for (*P*_3_)-**3**·L-**G17** (Figure 6d, purple line). The 328 nm band remained unchanged for (*P*_3_)-**3**·L-**G17** but intensified and shifted to 331 nm for (*M*_3_)-**3**·L-**G17**. Both titrations fit well to a 1:1 binding model (Figure 6e, entries 5 and 6), with an association constant for (*M*_3_)-**3**·L-**G17** nearly eight times higher than that for (*P*_3_)-**3**·L-**G17** (*K*_*M*_/*K*_*P*_ of 7.7), underscoring the strong enantioselectivity.

The characteristic spectral profiles observed for choline (**G16**) and (d)­l-**G17** suggest that α-hydroxy ammoniums salts interact uniquely with allenophane **3**.

DFT calculations showed that in complexes with **G13**–**G15**, allenophane (*P*_3_)-**3** adopts a *crown-like* conformation, with the ammonium cation nestled centrally and two alkyl chains extending outward to reduce steric strain (Figures 6f and S60–S65). This arrangement resembles a pseudorotaxane, where allenophane **3** acts as the wheel and the cation serves as the axle.

Optimized structures of [(*P*_3_)-**3**·**G16**]^+^ and [(*P*_3_)-**3**·**G17**]^+^ revealed that α-hydroxy ammonium guests induce a *helical* conformation in (*P*_3_)-**3**. One pyridine unit forms a hydrogen bond with the hydroxyl group of the guest side chain, leading to a nonthreaded assembly (Figure 6g). The resulting *C*_2_-symmetric helical conformation positions the alkoxy chain away from the pyridine unit to avoid repulsion with the nitrogen atom (Figure 6h–i).

While previous studies have reported enantioselective recognition of carnitine using nanomaterials or organic synthetic hosts,^56,57^ this work represents the first demonstration of such pronounced enantioselectivity for carnitine derivatives using a discrete molecular receptor.

Allenophane **3** exhibits remarkable selectivity by (1) differentiating ammoniums based on size and R-group bulk, favoring those with two methyl groups and linear chains that form pseudorotaxane-like structures; (2) distinguishing α-hydroxy ammoniums via diagnostic ECD shifts; and (3) exhibiting strong enantioselectivity toward l-carnitine derivatives.

These findings highlight its promise as a versatile and selective synthetic receptor for biologically relevant ammonium cations.

## Conclusions

We report the preparation, characterization, and application of chiral, shape-persistent cyclophanes—allenophanes—as powerful synthetic receptors for molecular recognition and chiral discrimination. Their unique 3D cavities arise from the intrinsic 90° axial twist of allenes, imparting a chiral topology that is inaccessible through conventional architectures. These features position allenophanes as valuable elements in the field of supramolecular chemistry.

A one-pot synthetic strategy was developed to efficiently access three discrete allenophanes **2**–**4** containing two, three- or four pyridine-allenyl monomer units, respectively. The use of Cs^+^ as a templating agent significantly enhanced overall yields and modulated product distribution, favoring the smaller dimer **2** in up to 49% yield, while suppressing the larger tetramer **4**. This templated approach allows for selective access to targeted macrocycles through control of reaction conditions.

The resulting allenophanes (*P*_2_)-**2** and (*P*_3_)-**3** were explored as synthetic hosts for biologically relevant guest molecules. Allenophane (*P*_2_)-**2** demonstrated high selectivity for small, neutral organic molecules bearing two hydrogen-bond donor groups, particularly catechol and its derivatives (e.g., CAPE). This recognition is governed by structural complementarity, where the two pyridine units engage the guest in a chelating fashion. Additionally, (*P*_2_)-**2** showed enantioselectivity toward 2-hydroxy-3-methylbutyric acid.

On the other hand, allenophane (*P*_3_)-**3** exhibited strong and selective binding toward ammonium cations (NR_4_)^+^, particularly those bearing two or three methyl groups. It clearly differentiates ammoniums with linear carbon chains from α-hydroxyammoniums such as choline and carnitine. These distinctions were revealed through diagnostic spectral changes at 315 nm in ECD spectroscopy, consistent with guest-induced conformation changes in the host. Notably, (*P*_3_)-**3** displayed exceptional enantioselectivity in recognizing an l-carnitine derivative, with a *K*_*M*_/K_*P*_ ratio of 7.7—highlighting the crucial role of its helical conformation in stereoselective interactions.

Overall, this study showcases the versatility, tunability, and precision of axially chiral allenophanes in host–guest chemistry. Their ability to discriminate guest molecules based on size, hydrogen-bonding patterns, and chirality underscore their potential as advanced tools for chiral sensing, molecular recognition, and supramolecular design.

## Supplementary Material



## Notes

General experimental methods, including synthesis conditions, characterization techniques, titration protocols, and computational details; optimization studies for the deprotection of monomer **1a**; compound characterization data including images of ^1^H and ^13^C NMR, UV–vis, and ECD spectra; X-ray crystallographic data; host–guest titration experiments monitored by NMR and ECD spectroscopy with mathematical fitting and species distribution plots; images of HRMS evidence for host–guest complexes with allenophane **3**; density functional theory (DFT) calculations of conformers and host–guest complexes, including optimized geometries, Cartesian coordinates, and calculated ECD spectra; ECD measurements of thin films (PDF)

## †

Departamento de Química Orgánica, Universidade de Santiago de Compostela, 15782 Santiago de Compostela, Spain
