# Supplementary material for: Twisted Allenyl-Pyridocyclophanes by Templated Cyclooligomerization: Chiral Cavities for Precision Molecular Recognition
Source: JACS Au. 2026 Jan 2;6(1):589–99. doi: 10.1021/jacsau.5c01555 (PMC12848741; doi:10.1021/jacsau.5c01555)
Supplement: Supplementary file 1 [file au5c01555_si_001.pdf]

Supporting Information

Twisted Allenyl-pyridocyclophanes by Templated Cyclooligomerization: Chiral Cavities for Precision Molecular Recognition

Jonathan Álvarez-García, María Magdalena Cid*

Departamento de Química Orgánica, Edificio Ciencias Experimentais, Campus Lagoas-Marcosende, Vigo E-36310, Spain, E-mail: mcid@uvigo.gal

Table of Contents

1. General Methods 2
   1.1. Synthesis 2
   1.2. Characterization techniques 2
   1.3. Titration experiments 2
   1.4. Density Functional Theory calculations 3
2. Synthetic procedures 3
   2.1. Optimization of the Deprotection Reaction of Monomer **1a** 3
   2.2. One-pot synthesis of allenophanes **2**, **3** and **4** 4
   2.3. One-pot templated synthesis of allenophanes **2** and **3** 8
   2.4. Synthesis of potential guests 10
3. Crystallographic data 15
4. Titrations experiments 20
   4.1. Allenophane 2 22
   4.2. Allenophane 3 30
5. Theoretical Calculations 41
   5.1. Conformers of (P_3)-**3** and (P_4)-**4** 41
   5.2. Complexes 69
6. ECD Measurements of Thin Films 111
7. References 115

1. General Methods

1.1. Synthesis

All reactions were carried out under nitrogen atmosphere unless otherwise stated. Reactions that required anhydrous conditions were carried out in oven-dried glassware at 120 °C for at least 24 hours. The reaction flasks were further dried by heating and subsequent cooling under a stream of nitrogen. The transfer of solvents or anhydrous solutions was carried out using syringes or cannulas, dried as described and stored in a desiccator with potassium hydroxide. Solvents were dried according to published methods and distilled before use.[1] Triethylamine was freshly distilled from CaH_2 under argon atmosphere. All other reagents were commercial compounds of the highest purity available. Silica gel 60F-254 Merck was used for thin layer chromatography and were visualized by exposure to UV light (254 nm) and revealed by treatment with a solution of phosphomolybdic acid or potassium permanganate. Merck silica gel 60 (230-240 mesh) was used under pressure for flash column chromatography.

1.2. Characterization techniques

^{1}H-NMR spectra were recorded at 25 ^{o}C (unless otherwise stated) on Bruker AMX-400 at 400 MHz with residual protic solvent as internal reference [$CDCl_3$, δH = 7.26 ppm], [D_2O, δ = 4.79 ppm]. Chemical shifts (δ) are given in parts per million (ppm) and coupling constants (*J*) are given in Hertz (Hz). The proton spectra are reported as follows: chemical shift δ (multiplicity, coupling constant J, number of protons). The following symbols were used for the description of coupling patterns: multiplet (m), singlet (s), doublet (d), triplet (t). **^{13}C-NMR spect**ra were recorded on the same spectrometer at 100 MHz at 25 ^{o}C (unless otherwise stated) with residual protic solvent as internal reference [$CDCl_3$, δ = 77.16 ppm]. **ECD** and **UV-Vis spectra** were recorded on a Jasco J-815 spectropolarimeter using a one-centimetre thick quartz cuvette at 25 °C (unless otherwise stated). The background was always obtained against the solvent. **ESI mass spectra** were recorded with an APEX3 instrument. Ions were generated using a Combi MALDI-- electrospray ionization (ESI) source. High-resolution mass spectra were taken on a VG Autospec instrument. **Crystallographic data** were collected at 100 K using a Bruker D8 Venture diffractometer with a Photon II CMOS detector and Mo-Kα radiation (λ = 0.71073 Å) generated by an Incoatec high brilliance microfocus source equipped with Incoatec Helios multilayer optics. The software APEX4[2] was used for collecting frames of data, indexing reflections, and determination of lattice parameters, SAINT[3] for integration of intensity of reflections, and SADABS[4] for scaling and empirical absorption correction. The structure was solved by dual space algorithm using the program SHELXT.[5] All non-hydrogen atoms were refined with anisotropic displacement parameters by full-matrix least-squares calculations on F2 using the program SHELXL[6] with OLEX2[7]. Hydrogen atoms were inserted at calculated positions and constrained with isotropic displacement; except for the hydrogen atoms of water molecule and the pyridinium proton, which were located from a Fourier-difference map and refined restraining the O-H and N-H distances. Drawings were produced with PLATON.[8]

1.3. Titration experiments

Solutions of the host compounds (allenophanes) were always prepared by weighing at least 5 mg of each host, using an analytical balance. The weighed host compound was dissolved in an appropriate solvent to make a stock solution of known concentration. This stock solution was then used to prepare a series of diluted solutions by using volumetric flasks or micropipettes to ensure accurate and consistent dilution. The same procedure was followed to prepare the solutions of the different guest molecules. Complexation experiments were monitored and analyzed using ECD/UV-Vis spectroscopy and/or NMR spectroscopy. In these experiments,

different additions of guest solutions were added to a host solution of appropriate concentration, in volumes ranging from 5 to 50 µL, using RAININ Pos·D positive displacement micropipettes. The mathematical fitting of the experimental data and the calculation of the corresponding association constants were performed using the BindFit software package, applying either a [1:1] or a [1:2] binding model, and employing the *Nelder–Mead* fitting algorithm.[9,10] Each of the titrations was repeated at least 3 times to ensure the reproducibility of the results. All host–guest processes described in this work are reversible. The hosts can be easily recovered after each titration experiment simply by washing the organic phase containing the complex with water (three times), which efficiently removes the excess guest molecules. All titrations were carried out at 25 °C unless otherwise stated.

1.4. Density Functional Theory calculations

Computational analyses were undertaken using the Gaussian 09 software package.[11] Structures of macrocycles and complexes were optimized without symmetry constraints using the CAM-B3LYP functional[12] with the 6-31g+(d,p) basis set, including solvation effects using the SMD model[13] with chloroform parameters. This level of calculation has already proven to be useful within the research group. for characterizing the different conformers of allenophanes as well as for reproducing their ECD spectra.[14] The diastereomeric complexes resulting from the interaction between allenophanes and chiral guests were re-optimized using the wB97XD hybrid functional[15] and the 6-31g+(d,p) basis set. The structures were characterized as minima in the potential surface by analytical computation of vibrational frequencies. Excited state energies and properties have been computed at TD-DFT CAM-B3LYP 6-31g+(d,p) level, including solvation effects using the SMD model with chloroform parameters. For circular dichroism spectra, the first 50 excited states were computed.

2. Synthetic procedures

2.1. Optimization of the Deprotection Reaction of Monomer **1a**

Table S1. Reaction conditions tested for the deprotection of **1a**.

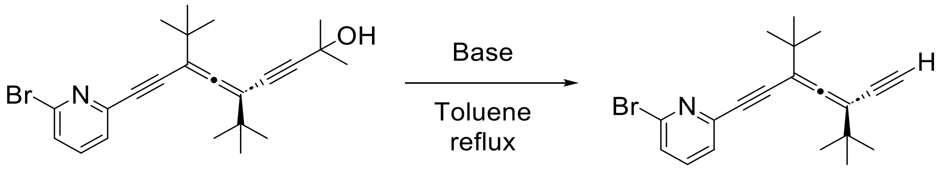

(*P*)-**1a** (*P*)-**1b**

Entry	Base	Time (h)	Yield **3.9b** (%)
**1**	**NaOH**	**1**	**97**
**2**	^{t}BuOK[a]	1	-
**3**	K_3PO_4	30	19[b]
**4**	K_2CO_3	30	22[b]
**5**	Cs_2CO_3	30	27[b]
**6**	NaH[a]	0.5	53

*General conditions: Monomer **1a** was dissolved in toluene (0.01 M) together with the appropriate base (300 eq) and heated at reflux under a N_2 atmosphere. [a]5 eq of base. [b]A portion of the starting material was recovered.*

Entry 1: Pulverized NaOH (2 g, 50 mmol, 300 eq) was placed into a 100 mL round-bottom flask and flamed while purging with N_2. (*P*)-**1a** (72 mg, 0.17 mmol, 1 eq) was also purged with N_2, dissolved in dry toluene (20 mL) and transferred *via* cannula to the previous flask. The reaction mixture was stirred and refluxed for 1 h. Then, the solvent was removed under reduce pressure and distilled water was added (20 mL). The aqueous phase was extracted with AcOEt (20 mL x 3). The organic phase was dried with $Na_2SO_{4(anh)}$ and the solvent removed under reduced pressure. (*P*)-**1b** was obtained as a white solid in 97% yield (59 mg) and no further purification was needed.[16]

2.2. One-pot synthesis of allenophanes **2**, **3** and **4**

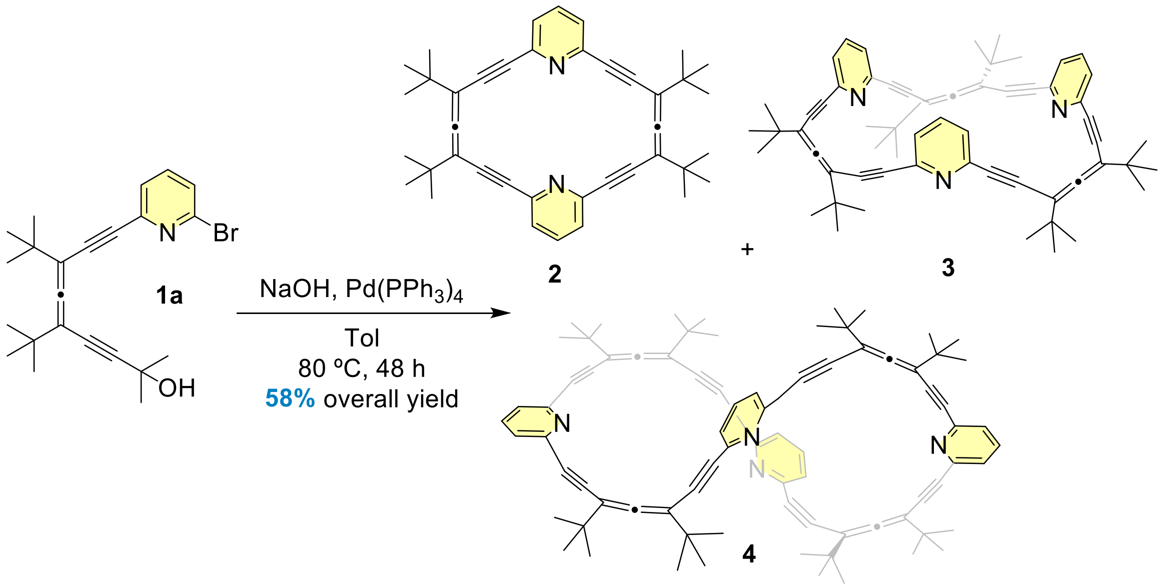

Pulverized NaOH (3.6 g, 90 mmol, 300 eq) was placed into a 250 mL round-bottom flask and flamed while purging with N_2. A solution of monomer **1a**[16] (125 mg, 0.30 mmol, 1 eq) and $Pd(PPh_3)_4$ (35 mg, 0.03 mmol, 0.1 eq) in dry toluene (60 mL) was added to the flask. The reaction mixture was stirred at 80 °C for 48 h. Then, the solvent was removed under reduce pressure and distilled water (100 mL) was added carefully, placing the reaction flask in an ice bath. The aqueous phase was extracted with AcOEt (100 mL x 3). The organic phase was dried with $Na_2SO_{4(anh)}$ and the solvent removed under reduced pressure. The remaining solid was purified by flash chromatography (SiO_2, Hex:Acetone 85:15) to give **2** as a white solid in 22% yield (18 mg), **3** as a white solid in 19% yield (15 mg) and **4** as a white solid in 17% yield (14 mg). Each of the enantiomers of **1a** (*P* or *M*) was taken as a starting point to characterize each homochiral enantiomer of each of the macrocycles.

(P_2)-**2**/(M_2)-**2**: **^{1}H-NMR** (400 MHz, $CDCl_3$) δ= 7.59 (t, *J* = 7.8 , 2H, Ar), 7.27 (d, *J* = 7.8 , 4H, Ar), 1.21 (s, 36H, *t*Bu). **^{13}C-NMR** (100 MHz, $CDCl_3$) δ 214.5 (C), 144.8 (C), 136.5 (CH), 124.7 (CH), 103.8 (C), 92.8 (C), 83.9 (C), 35.3 (C), 29.3 (tBu). **UV/Vis** ($CHCl_3$): λ_{max} (ε)= 264.0 nm (22925 mol^{-1} dm^3 cm^{-1}), 288.0 nm (18428 mol^{-1} dm^3 cm^{-1}), 313.0 nm (7138 mol^{-1} dm^3 cm^{-1}). **HRMS-ESI**: m/z calcd. for $C_{40}H_{43}N_2^+$ 551.3431; found 551.3421 $[M+H]^+$.[16]

(P_3)-**3**/(M_3)-**3**: **^{1}H-NMR** (400 MHz, $CDCl_3$): δ 7.56 (t, *J* = 7.8, 3H, Ar), 7.32 (d, *J* = 7.8, 6H, Ar), 1.22 (s, 54H, ^{t}Bu). **^{13}C-NMR** (100 MHz, $CDCl_3$): δ 213.0 (C), 144.1 (C), 136.0 (CH), 126.2 (CH), 103.4 (C), 92.1 (C), 83.4 (C), 35.9 (C), 29.2 (^{t}Bu). **UV/Vis** ($CHCl_3$): λ_{max}(ε)= 252 nm (34950 mol^{-1} dm^{3} cm^{-1}), 284 nm (40246 mol^{-1} dm^{3} cm^{-1}), 313 nm (35644 mol^{-1} dm^{3} cm^{-1}). **HRMS-ESI**: *m/z* calcd. for $C_{60}H_{64}N_3^+$ 826.5094; found 826.5107 [M+H]$^+$, 848.4914 [M+Na]$^+$ and 413.7584 [M+H$_2$]$^{+2}$.

(P_4)-**4**/(M_3)-**4**: **^{1}H-NMR** (400 MHz, $CDCl_3$): δ 7.52 (t, *J* = 7.7, 4H, Ar), 7.40 (d, *J* = 7.7, 8H, Ar), 1.21 (s, 72H, ^{t}Bu). **^{13}C-NMR** (100 MHz, $CDCl_3$): δ 213.3 (C), 144.1 (C), 136.3 (CH), 126.5 (CH), 103.5 (C), 92.1 (C), 83.7 (C), 35.8 (C), 29.4 (^{t}Bu). **UV/Vis** ($CHCl_3$): λ_{max}(ε)= 250 nm (59231 mol^{-1} dm^{3} cm^{-1}), 283 nm (60634 mol^{-1} dm^{3} cm^{-1}), 299 nm (57862 mol^{-1} dm^{3} cm^{-1}), 319 nm (53261 mol^{-1} dm^{3} cm^{-1}). **HRMS-ESI**: *m/z* calcd. for $C_{80}H_{85}N_4^+$ 1101.6769; found 1101.6777 [M+H]$^+$ and 551.3421 [M+H$_2$]$^{+2}$.

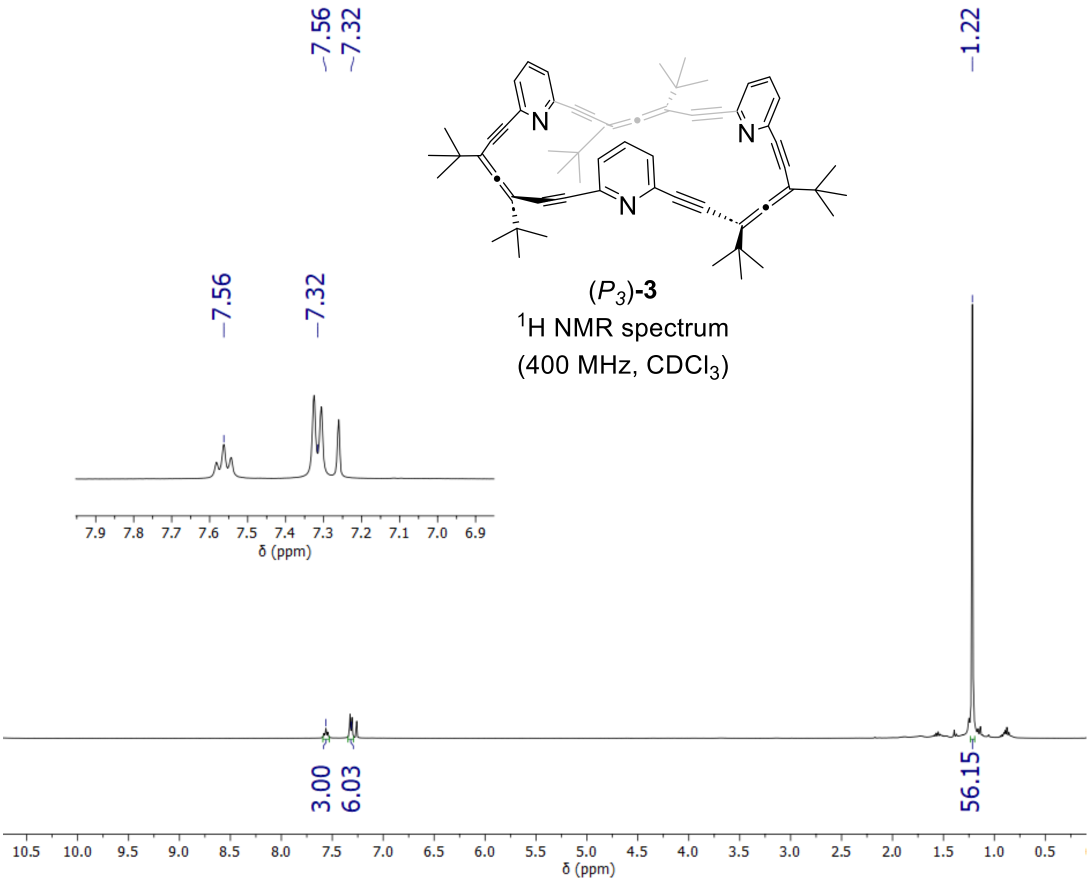

Figure S1. (P_3)-**3** ^{1}H-NMR ($CDCl_3$) spectrum.

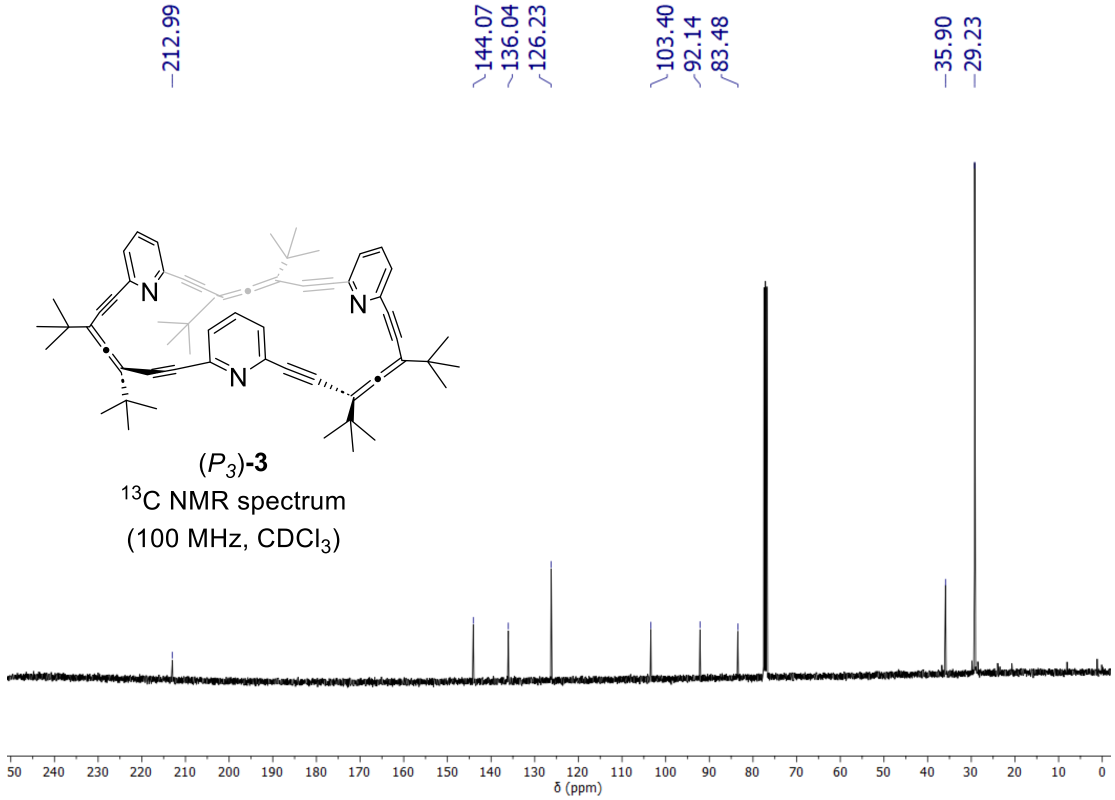

Figure S2. (P_3)-**3** ^{13}C-NMR ($CDCl_3$) spectrum.

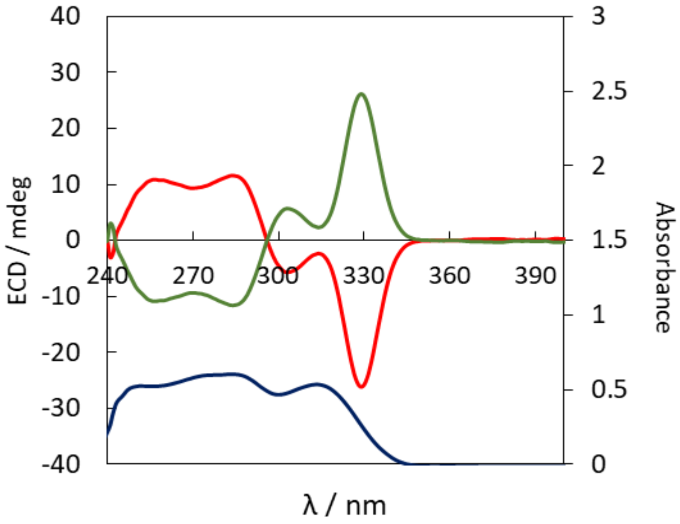

Figure S3. Red line (P_3)-**3** and green line (M_3)-**3** ECD spectrum. Blue line (P_3)/(M_3)-**3** UV/Vis spectrum [Chloroform, $1.5 \cdot 10^{-5}$M].

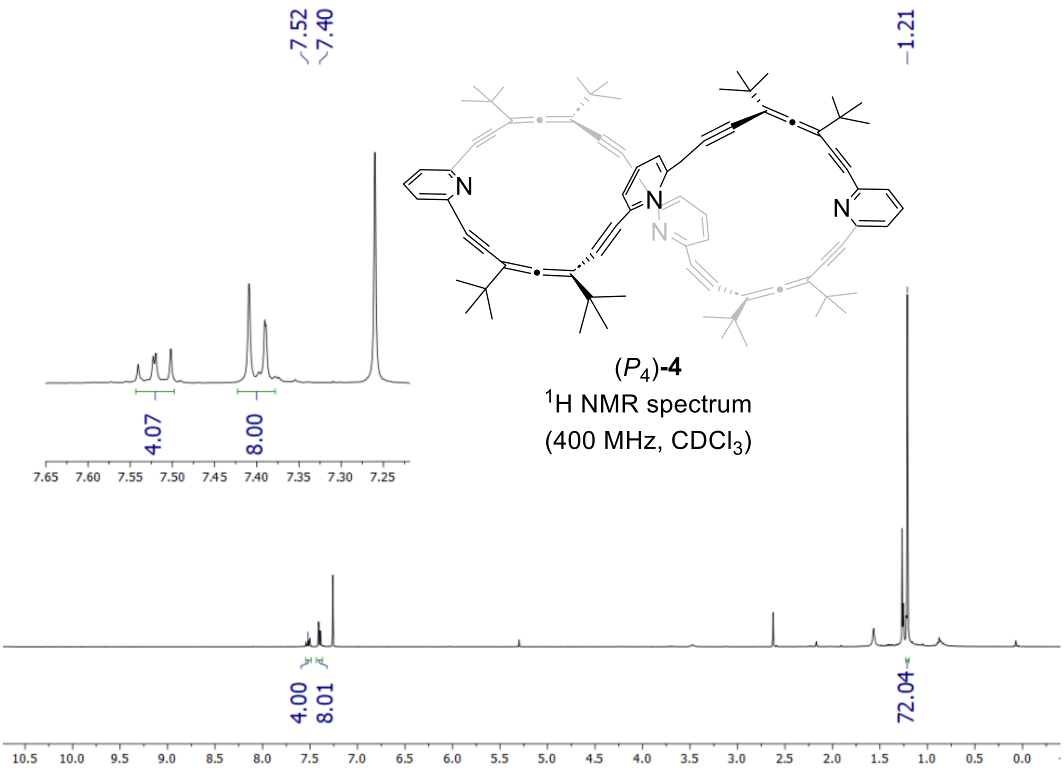

Figure S4. (P_4)-**4** ^{1}H-NMR ($CDCl_3$) spectrum.

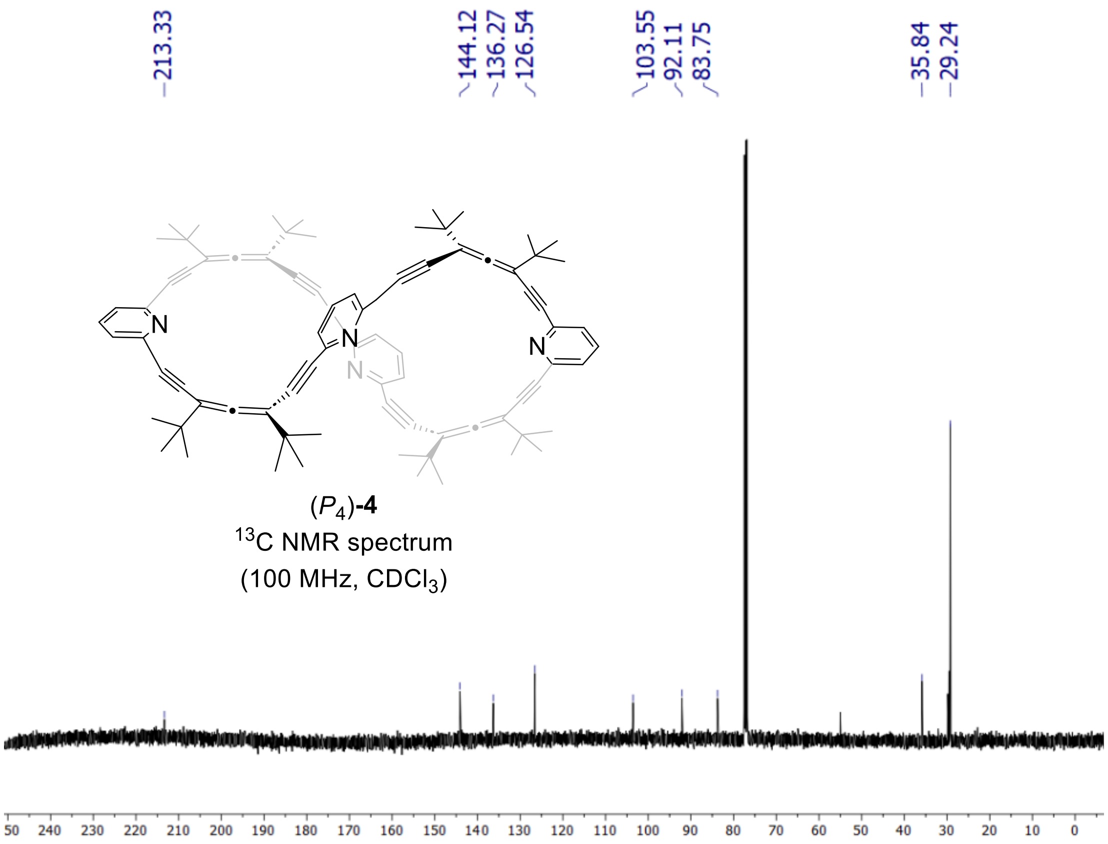

Figure S5. (P_4)-**4** ^{13}C-NMR ($CDCl_3$) spectrum.

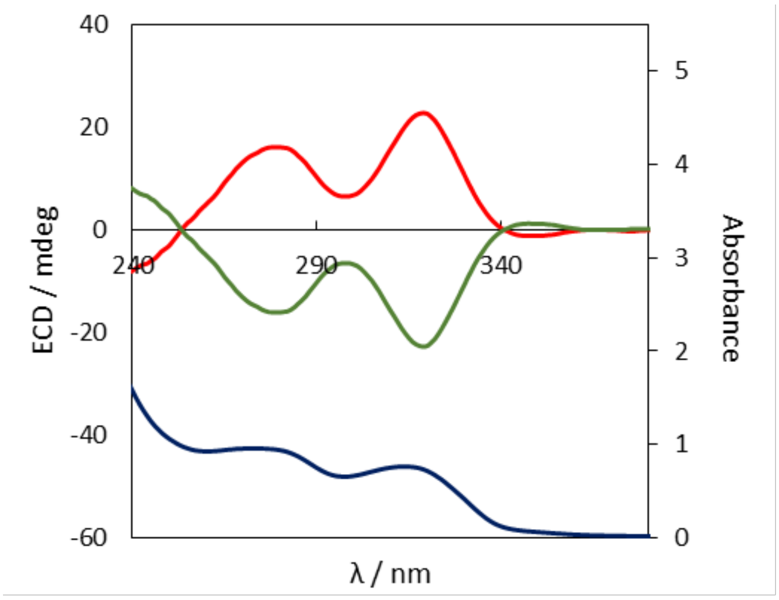

Figure S6. Red line (P_4)-**4** and green line (M_4)-**4** ECD spectrum. Blue line (P_4)/(M_4)-**4** UV/Vis spectrum [CH_3CN, $4.0\cdot10^{-6}$M].

2.3. One-pot templated synthesis of allenophanes **2** and **3**

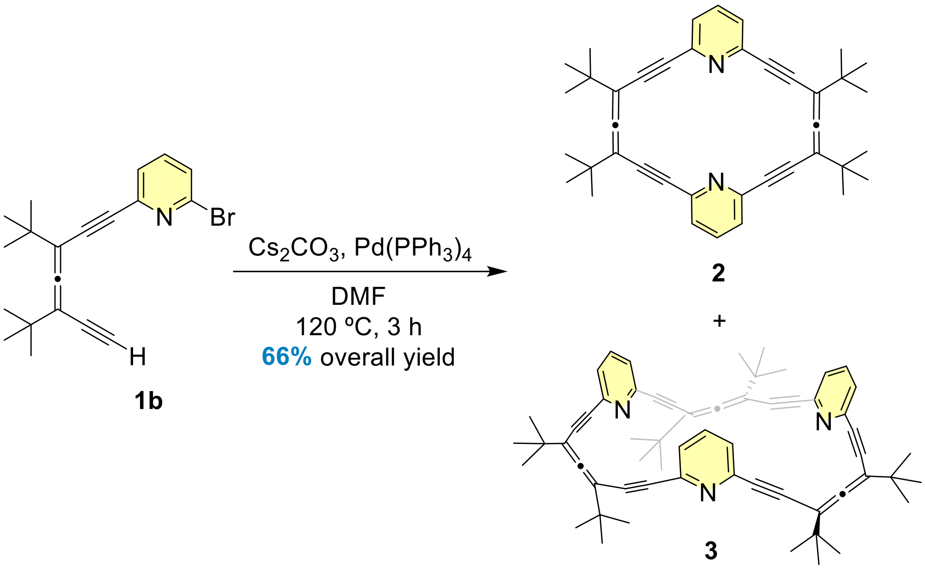

Monomer **1b** (50 mg, 0.14 mmol, 1 eq), $Pd(PPh_3)_4$ (17 mg, 0.014 mmol, 0.1 eq) and Cs_2CO_3 (272 mg, 0.84 mmol, 6 eq) were placed into a 100 mL round-bottom flask previously flamed and purged with N_2. Dry DMF (30 mL) was added and the reaction mixture was stirred at 120 °C for 3 h. Then, the solvent was removed under reduced pressure. DCM (25 mL) and distilled water (25 mL) were added. Both phases were separated, and the aqueous phase was further extracted with DCM (25 mL x2). The organic phase was dried with $Na_2SO_{4(anh)}$ and the solvent removed under reduced pressure. The remaining solid was purified by flash chromatography (SiO_2, Hex:Acetone 85:15) to give **2** as a white solid in 47% yield (18 mg) and **3** as a white solid in 19% yield (8 mg).

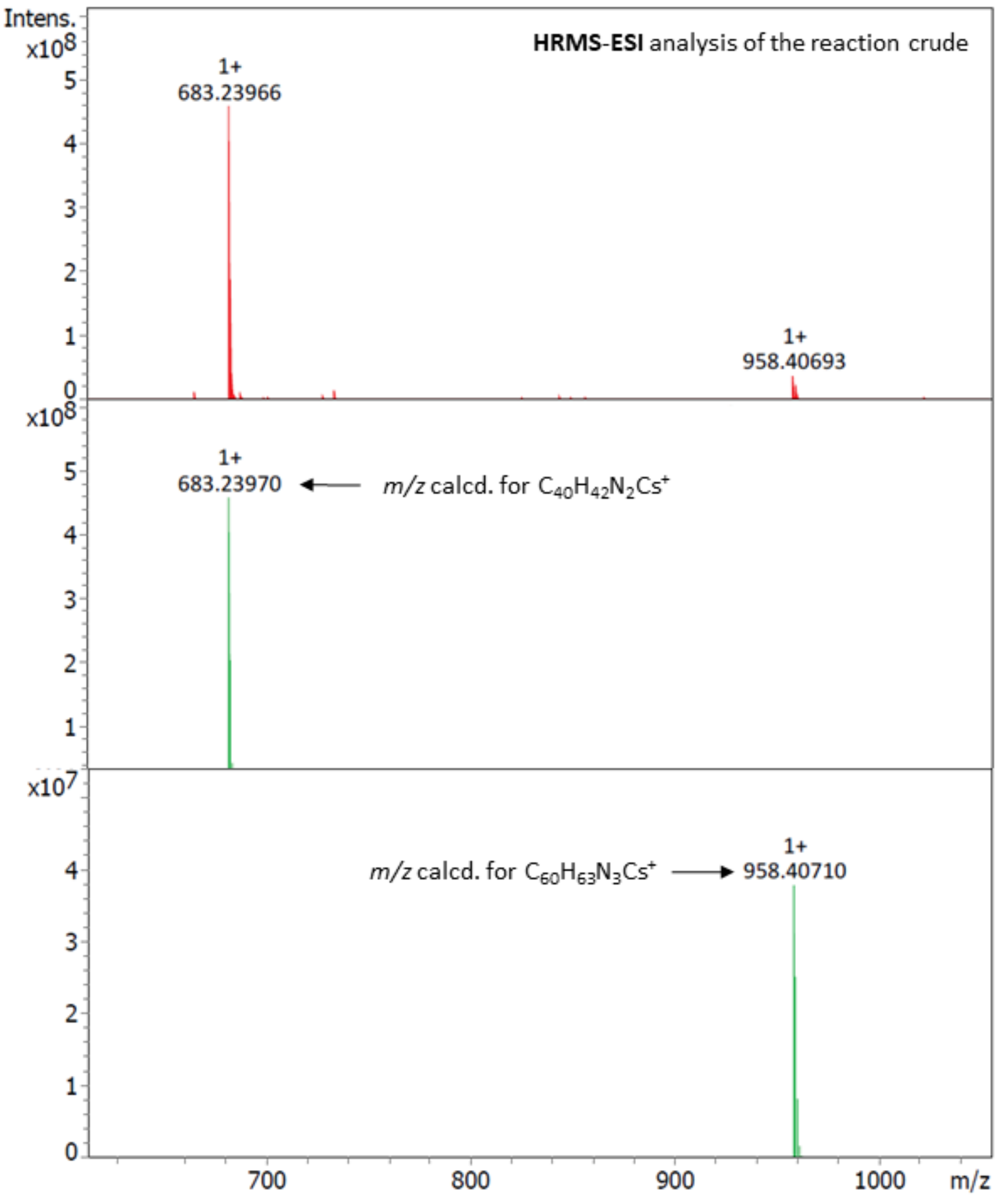

Figure S7a. HRMS-ESI analysis of the reaction crude.

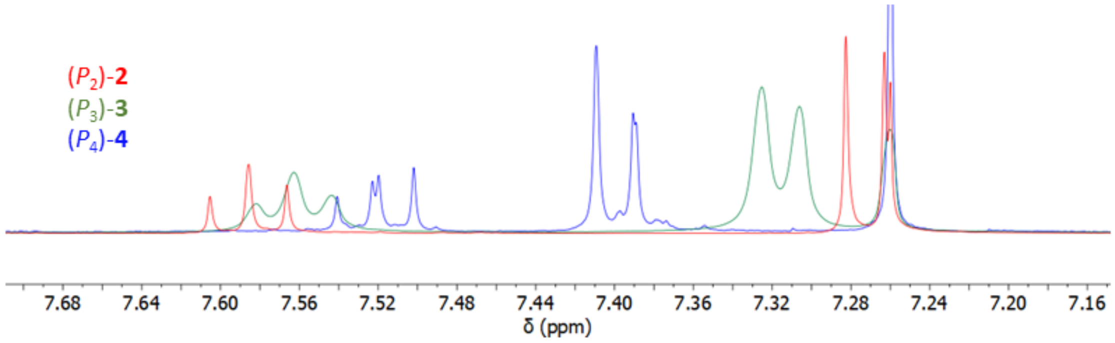

Figure S7b. Comparison of the ^{1}H-NMR spectrum ($CDCl_3$) of (P_2)-**2**, (P_3)-**3** and (P_4)-**4**.

2.4. Synthesis of potential guests

Synthesis of choline 2-hydroxyisobutyrate (**G8**)

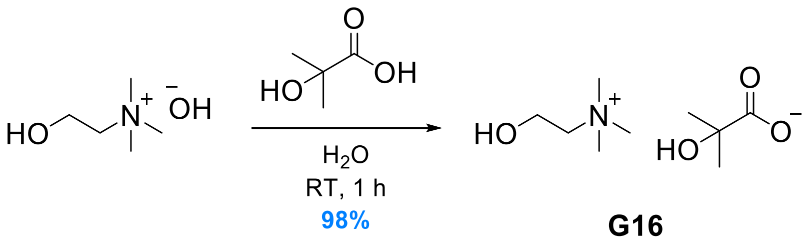

G16

In a 25 mL round-bottom flask, a choline hydroxide solution (2 mL, 46 wt. % in H_2O, 8.25 mmol, 1 eq) was added. Distilled water (10 mL) and 2-hydroxyisobutyric acid (860 mg, 8.25 mmol, 1 eq) were added, and the mixture was stirred at room temperature for 1 h. Then, the solvent was removed under reduced pressure. The resulting residue was washed with Et_2O (10 mL x 2) and dried under reduced pressure at 60 °C overnight. Choline 2-hydroxyisobutyrate (**G16**) was obtained as a yellowish liquid in 98% yield (1.66 g) and was stored under N_2 atmosphere.

G16: ^{1}H-NMR (400 MHz, D_2O): δ 4.03 (m, 2H, -CH_2-), 3.49 (m, 2H, -CH_2-), 3.18 (s, 9H, -CH_3), 1.34 (s, 6H, -CH_3). **^{13}C-NMR** (100 MHz, D_2O): δ 183.6 (C), 73.5 (C), 67.4 (-CH_2-), 55.6 (CH_3), 53.8 (CH_3), 26.7 (C). **Elemental analysis**: calcd. for $C_9H_{21}NO_4$ C, 52.15; H, 10.21; N, 6.76; found C, 51.83; H, 10.11; N, 6.75.

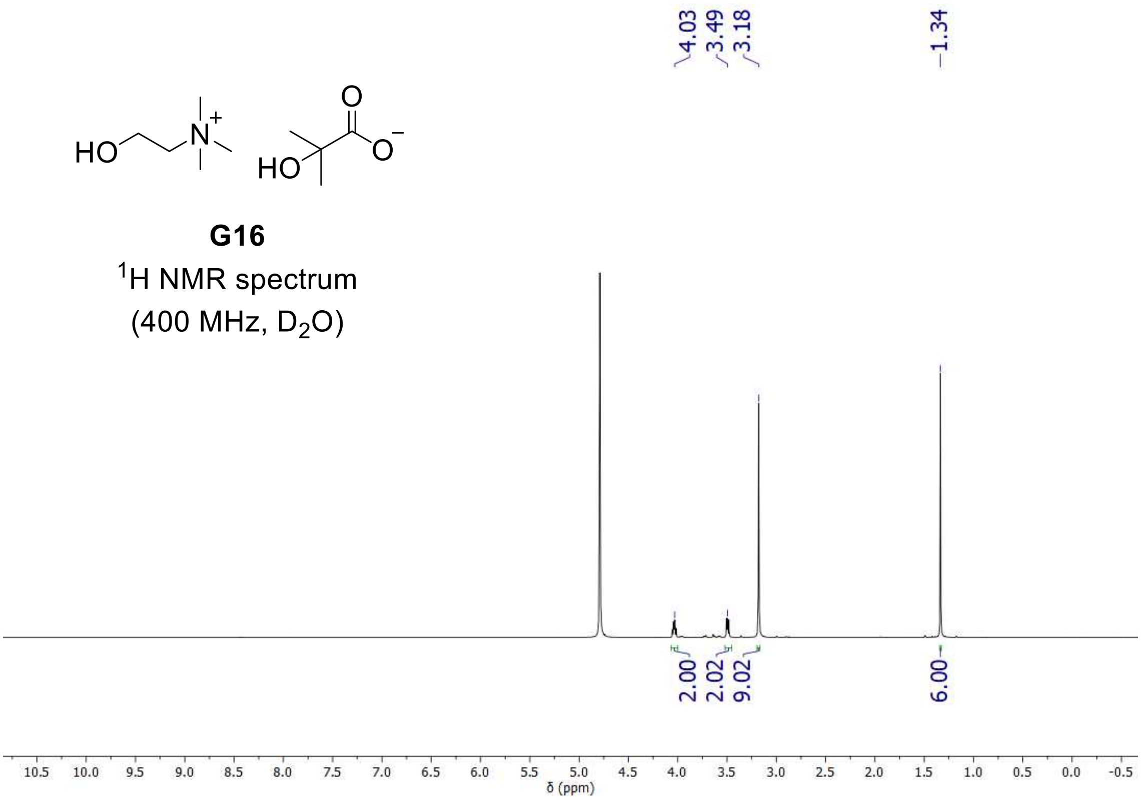

Figure S8. G16 ^{1}H-NMR (D_2O) spectrum.

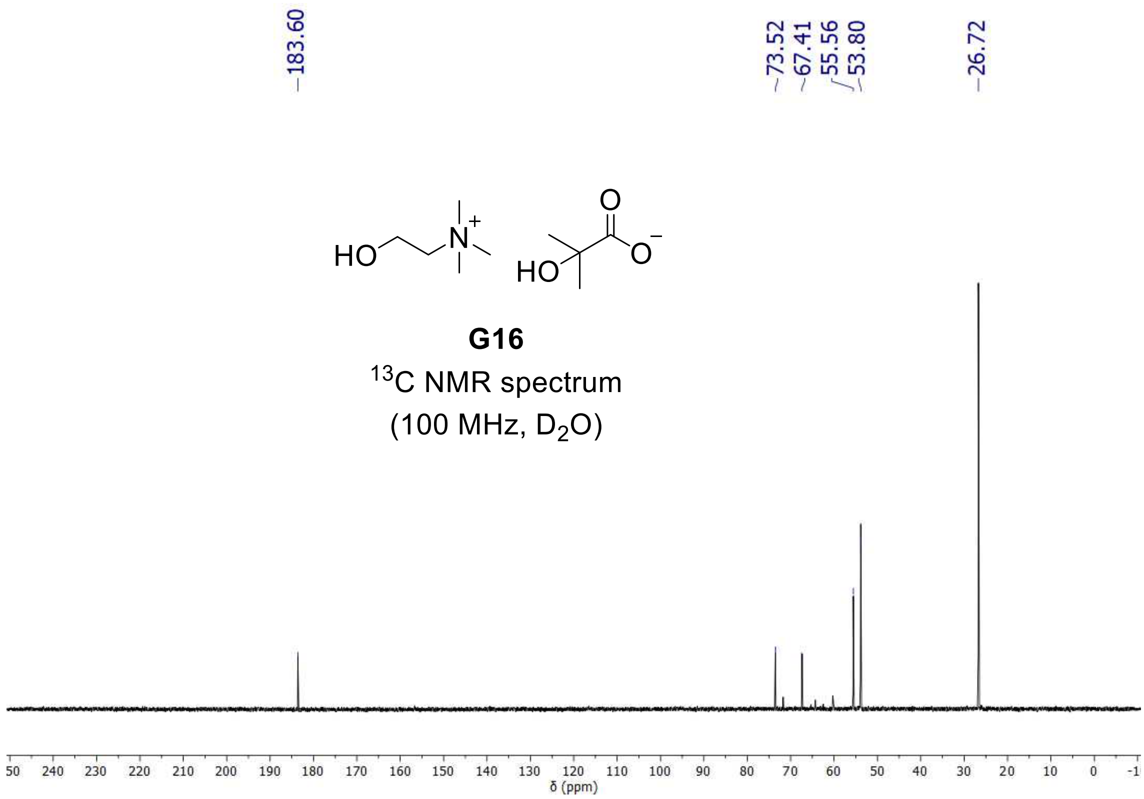

Figure S9. G16 ^{13}C-NMR (D_2O) spectrum.

Synthesis of N,N-dicyclohexylmethylammonium 2-hydroxyisobutyrate (**G10**)

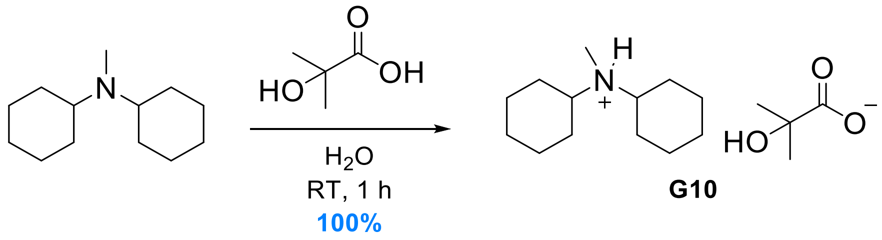

N,*N*-dicyclohexylmethylamine (1 g, 5.12 mmol, 1 eq) and 2-hydroxyisobutyric acid (533 mg, 5.12 mmol, 1 eq) were added into a 25 mL round-bottom flask. Then, distilled water (15 mL) was added and the mixture was stirred at room temperature for 1 hour. The solvent was removed under reduced pressure. The resulting residue was washed with Et_2O (10 mL x2) and dried under reduced pressure at 60 °C overnight. **G10** was obtained as a colorless liquid in 100% yield (1.53 g) and was stored under N_2 atmosphere.

G10: ^{1}H-NMR (400 MHz, D_2O): δ 3.37 (m, 2H, -CH-), 2.72 (s, 3H, $-CH_3$), 2.03 (m, 4H, $-CH_2-$), 1.88 (m, 4H, $-CH_2-$), 1.67 (m, 4H, $-CH_2-$), 1.51-1.29 (m, 8H, $-CH_2-$), 1.35 (s, 3H, $-CH_3$), 1.17 (m, 2H, $-CH_2-$). **^{13}C-NMR** (100 MHz, D_2O): δ 184.0 (C), 73.7 (C), 62.1 (CH), 31.9 (CH_3), 27.3 ($-CH_2-$), 26.7 (CH_3), 24.5 ($-CH_2-$). **Elemental analysis**: calcd. for $C_{17}H_{33}NO_3$ C, 68.19; H, 11.11; N, 4.68; found C, 67.78; H, 11.01; N, 4.64.

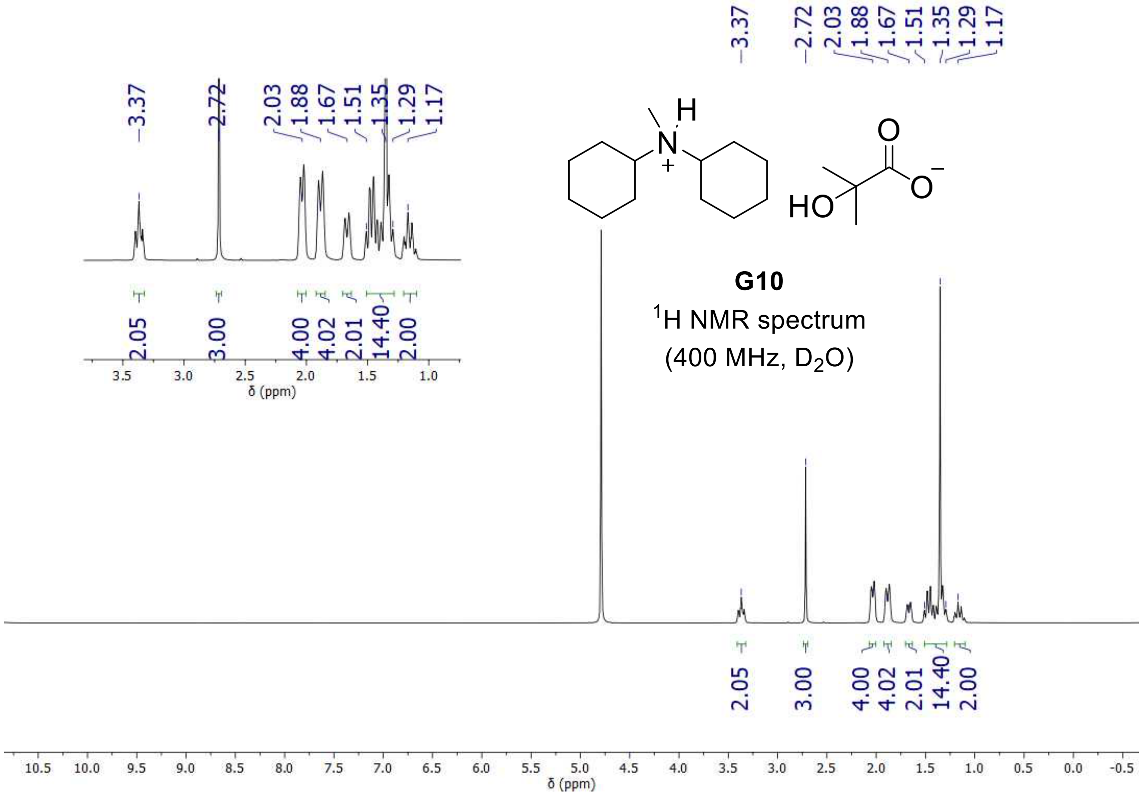

Figure S10. G10 ^{1}H-NMR (D_2O) spectrum.

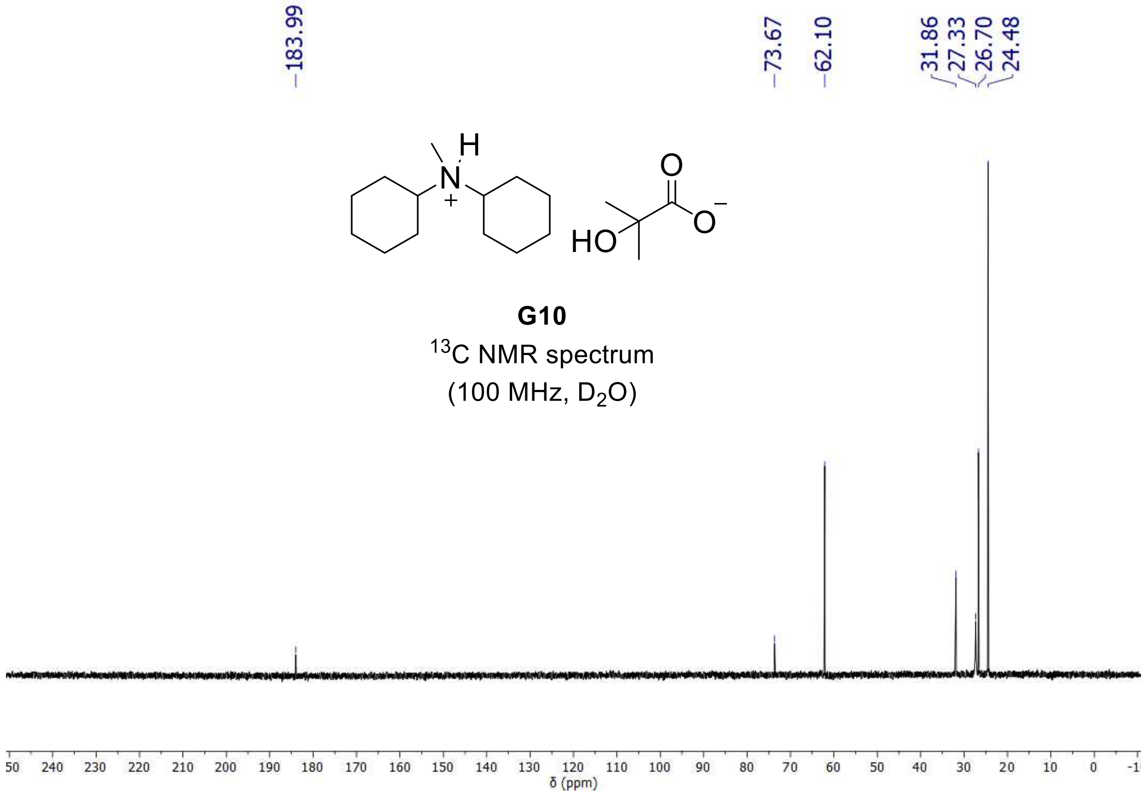

Figure S11. G10 ^{13}C-NMR (D_2O) spectrum.

Synthesis of L-**G17**

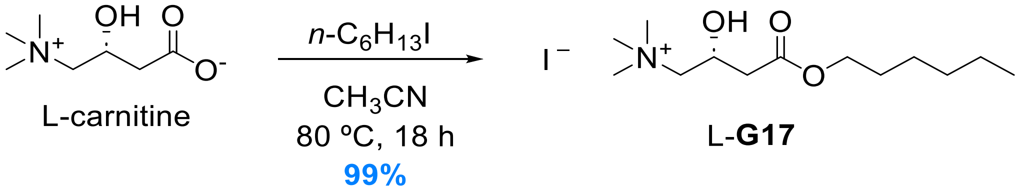

L-carnitine (200 mg, 1.24 mmol, 1 eq) was added into a 50 mL Schleck tube under N_2 atmosphere. Then, dry acetonitrile (15 mL) and 1-iodohexane (200 µL, 1.36 mmol, 1.1 eq) were added and the suspension was stirred at 80 °C for 18 h. The solvent was removed under reduced pressure. The resulting residue was washed with Et_2O (10 mL x2) and dried under reduced pressure. L-**G17** was obtained as a colorless oil in 99% yield (457 mg) and was stored under N_2 atmosphere.

G17: ^{1}**H-NMR** (400 MHz, $CDCl_3$): δ 4.74 (m, 1H, -CH-), 4.07 (td, J = 6.9, 1.9 Hz, 2H, -CH_2-), 3.84 (d, J = 13.0 Hz, 1H, -CH_2-), 3.69 (dd, J = 13.0, 10.0 Hz, 1H, -CH_2-), 3.48 (s, 9H, -CH_3), 2.70 (qd, J = 16.8, 6.4 Hz, 2H, -CH_2-), 1.61 (m, 2H, -CH_2-), 1.37 – 1.20 (m, 6H, -CH_2-), 0.92 – 0.80 (m, 3H, -CH_3). ^{13}**C-NMR** (100 MHz, $CDCl_3$): δ 171.3 (C), 69.4 (-CH_2-), 65.6 (-CH_2-), 62.9 (-CH-), 55.5 (-CH_3), 39.5 (-CH_2-), 31.5 (-CH_2-), 28.5 (-CH_2-), 25.6 (-CH_2-), 22.6 (-CH_2-), 14.1 (-CH_3). **HRMS-ESI**: m/z calcd. for $C_{13}H_{28}NO_3^+$ 246.2064; found 246.2068 $[M]^+$.

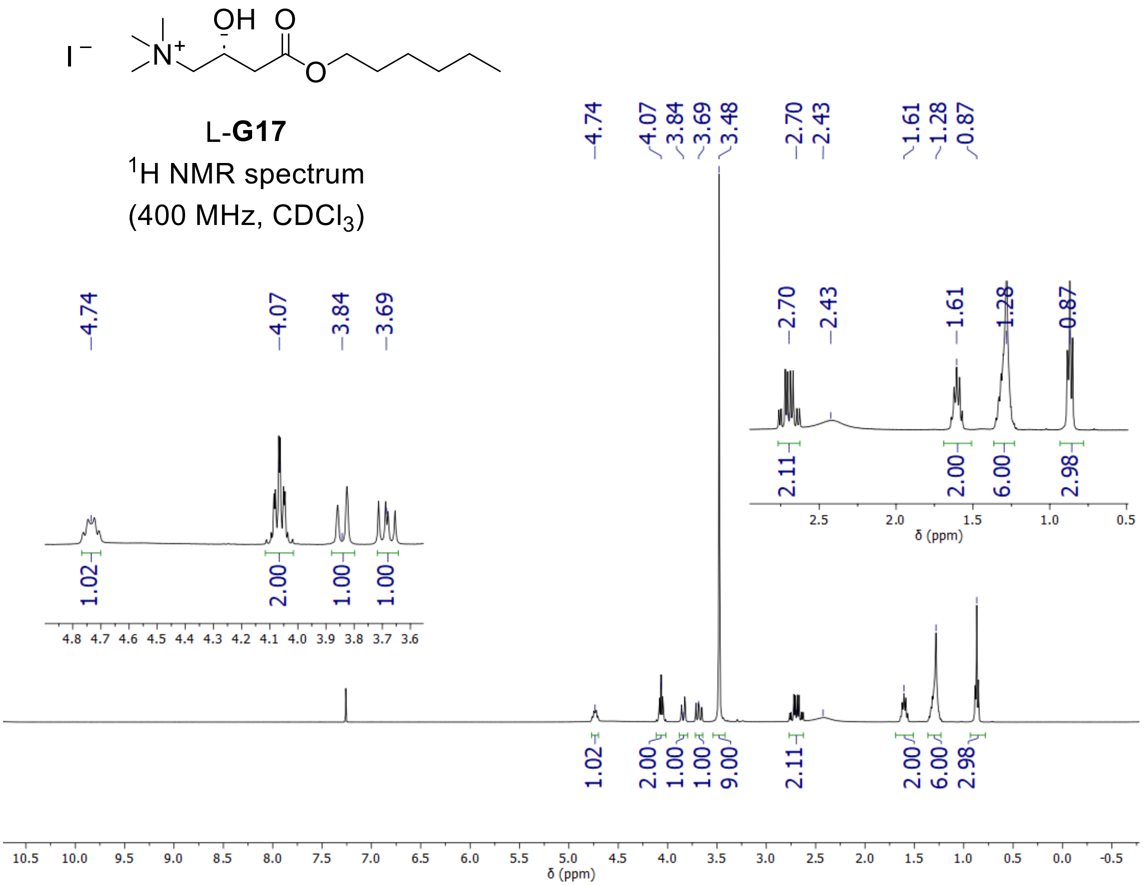

Figure S12. G17 ^{1}H-NMR ($CDCl_3$) spectrum.

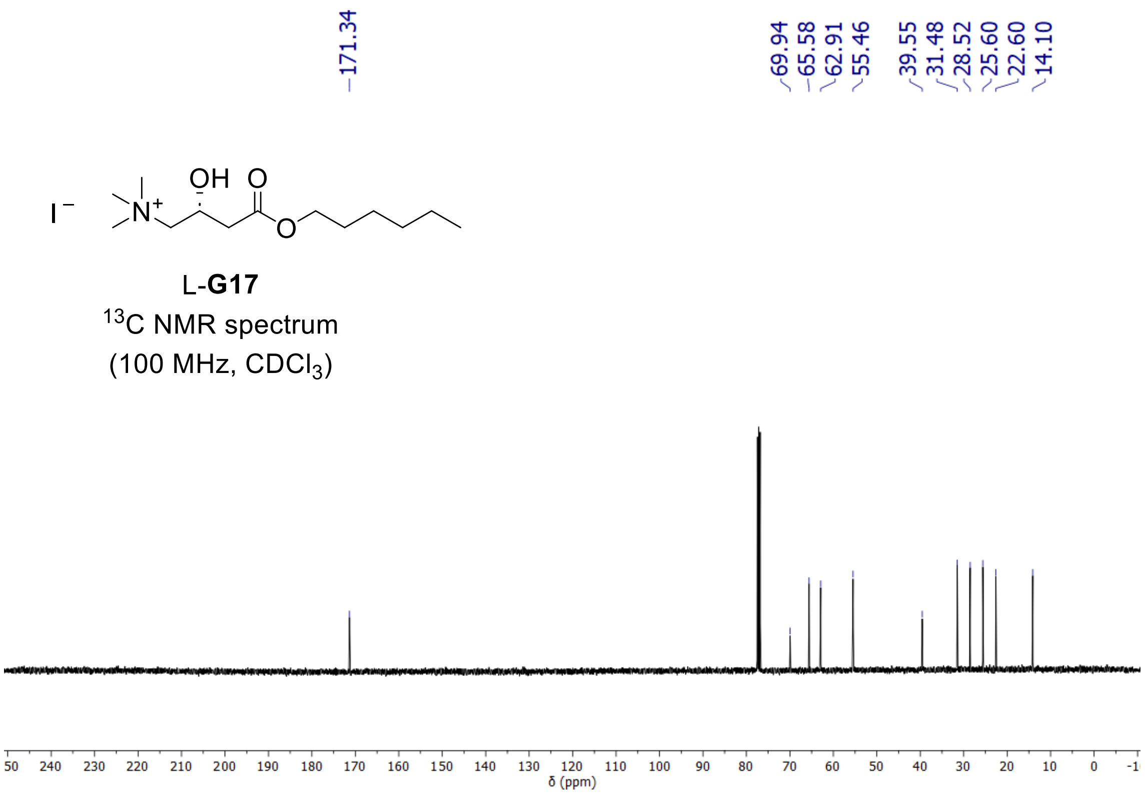

Figure S13. G17 ^{13}C-NMR ($CDCl_3$) spectrum.

3. Crystallographic data

Homochiral racemate allenophane **3**

A solution of the stereoisomers of **3** was prepared in MTBE and allowed to slowly concentrate over a week at room temperature, leading to the growth of crystals of the racemic mixture (P_3)/(M_3)-**3.**

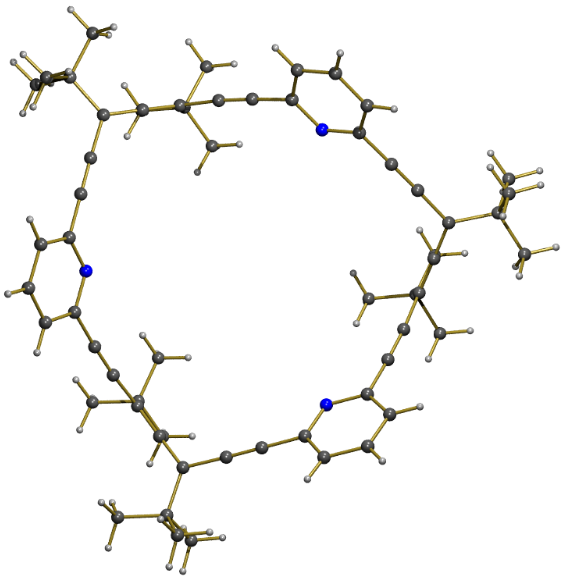

Figure S14. Crystal structure of allenophane **3**.

Table S2. Crystal data and structure refinement for (P_3)/(M_3)-**3**

Empirical formula	$C_{60}H_{63}N_3$
Formula weight	826.13
Temperature	100.00 K
Wavelength	0.71073 Å
Crystal system	Trigonal
Space group	R -3
Unit cell dimensions	a = 17.3327(4) Å, α= 90°
	b = 17.3327(4) Å, β= 90°
	c = 35.5914(13) Å, γ = 120°
Volume	9259.9(5) Å^3
Z	6
Density (calculated)	0.889 Mg/m^3
Absorption coefficient	0.051 mm^{-1}
F(000)	2664
Crystal size	0.234 x 0.152 x 0.138 mm^3
Theta range for data collection	2.350 to 25.366°.
Index ranges	-20<=h<=20, -20<=k<=20, -42<=l<=42
Reflections collected	41581
Independent reflections	3766 [R(int) = 0.0423]
Completeness to theta = 25.242°	99.9 %
Absorption correction	None
Max. and min. transmission	0.6895 and 0.6354
Refinement method	Full-matrix least-squares on F^2

Data / restraints / parameters	3766 / 0 / 197
Goodness-of-fit on F^2	1.063
Final R indices [I>2sigma(I)]	R1 = 0.0762, wR2 = 0.2335
R indices (all data)	R1 = 0.0848, wR2 = 0.2456

Non-classical hydrogen bonds (**Table S3**), involving a –CH of a methyl group as donor and the N atom of the pyridine ring of neighboring macrocycle as acceptor, generate a discrete dimer.

Table S3. Self-assembled racemic dimmers trough non-classical hydrogen bonds (blue dashed lines). Bond parameters. D = donor, A = acceptor.

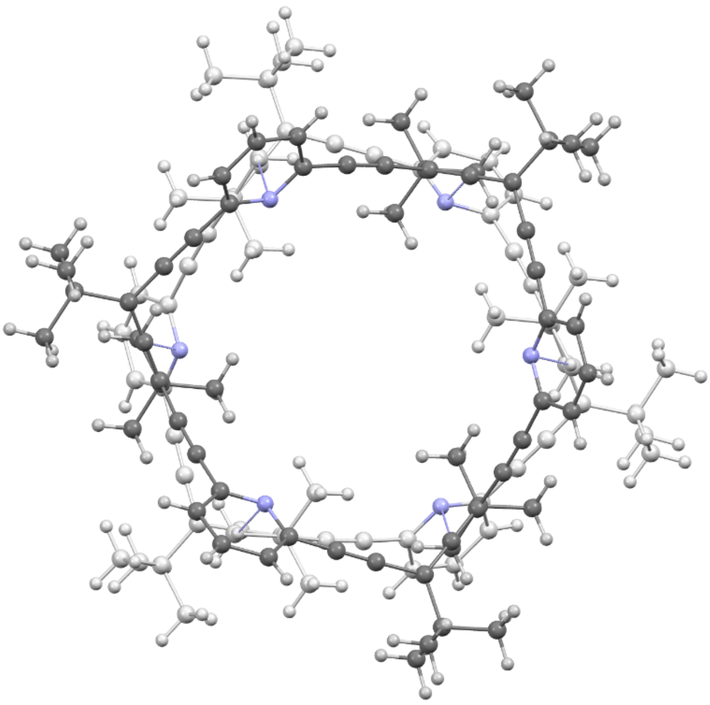

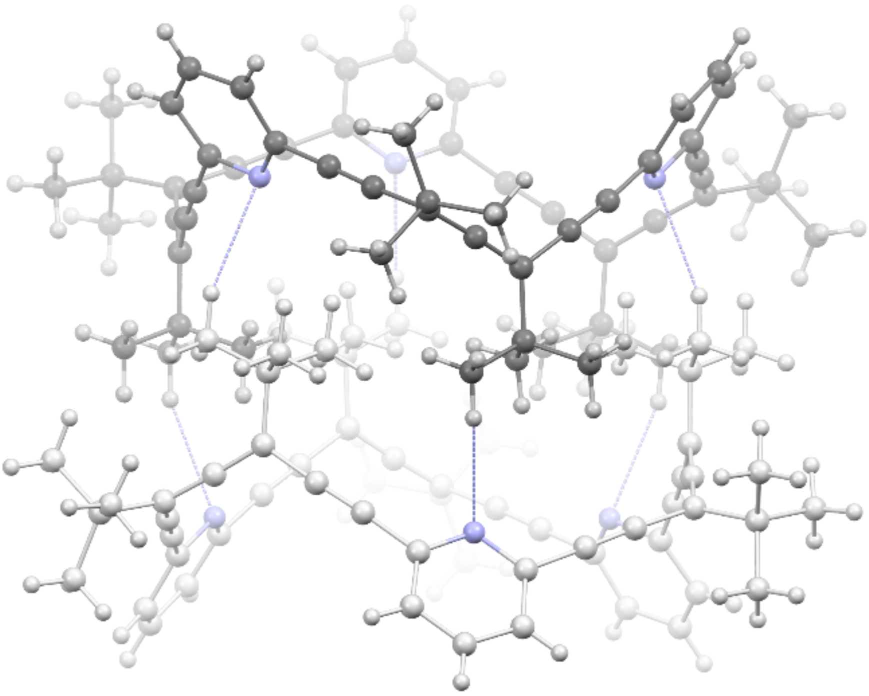

D-H···A	d(D-H)	d(H···A)	d(D···A)	∠(DHA)
CH···N	0.98 Å	2.66 Å	3.592(3) Å	158.2°

The dimers are connected by C-H···π interactions (**Table S4**), resulting in a 3D network. The C-H···π interactions found are of two kinds: the first one involves a –CH methyl as donor and the pyridine ring of a neighbouring dimer as acceptor, whereas in the second, the donor is a -CH group of the pyridine ring and the acceptor is an alkyne of a neighbouring dimer.

Table S4. View and parameters of CH···π (pyridine) and C-H···π (alkyne) interactions. D = donor, A = acceptor.

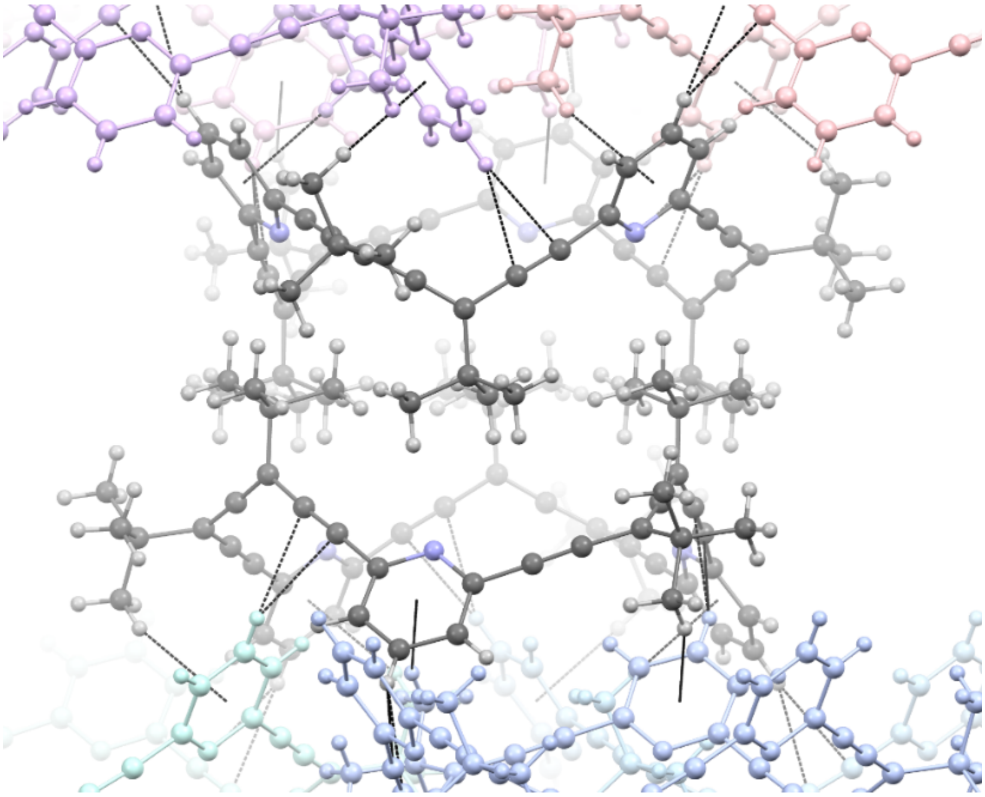

C-H···π	d(D-H)	∠ (CHA)
C-H···(py)	2.79 Å[a]	159°
C-H···(-C_1≡C_2-)	2.77 Å[b]	153.7°
	2.83 Å[c]	156.4°

[a]*Distance between the hydrogen atom and the center of a pyridine ring.* [b]*Distance between the hydrogen atom and one of the carbons in the alkyne C_1.* [c]*Distance between the hydrogen atom and the other carbon of the alkyne C_2.*

Crystallographic data of [(*rac*)-**2·G1**] complex

The mixture of stereoisomers of [7_2]-pyrido-allenophane **2** was dissolved in a mixture of heptane, MTBE and methanol (1.6:0.4:1). Catechol **G1** (1 eq) was added. Then, the solution was allowed to concentrate at room temperature for 3 days, leading to the growth of crystals of the [(P_2)/(M_2)-**2·G1**l] complex.

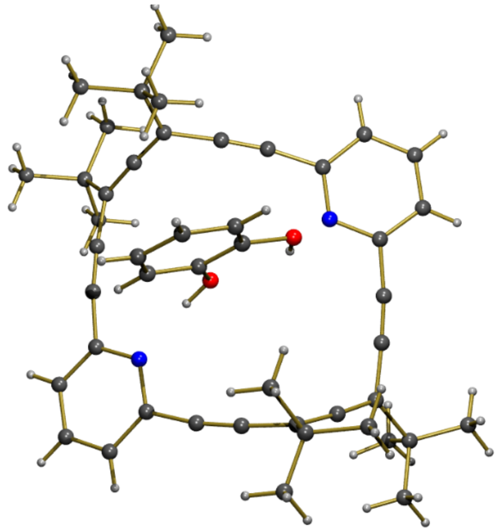

Figure S15. Crystal structure of [**2·G1**] obtained by X-ray diffraction.

Table S5. Crystal data and structure refinement for [**2·G1**]

Empirical formula	$C_{46}H_{48}N_2O_2$
Formula weight	660.86
Temperature	294.00 K
Wavelength	0.71073 Å
Crystal system	Orthorhombic
Space group	Pbca
Unit cell dimensions	a = 24.3901(10) Å, α= 90°
	b = 11.2361(4) Å, β= 90°
	c = 31.1281(13) Å, γ = 90°
Volume	8530.6(6) Å^3
Z	8
Density (calculated)	1.029 Mg/m^3
Absorption coefficient	0.062 mm^{-1}
F(000)	2832
Crystal size	0.208 x 0.057 x 0.055 mm^3
Theta range for data collection	2.100 to 25.350°.
Index ranges	-29<=h<=29, -13<=k<=12, -37<=l<=37
Reflections collected	83667
Independent reflections	7815 [R(int) = 0.0985]
Completeness to theta = 25.242°	99.9 %
Absorption correction	Semi-empirical from equivalents
Max. and min. transmission	0.7378 and 0.6690

Refinement method	Full-matrix least-squares on F^2
Data / restraints / parameters	7815 / 74 / 532
Goodness-of-fit on F^2	1.014
Final R indices [I>2sigma(I)]	R1 = 0.0607, wR2 = 0.1395
R indices (all data)	R1 = 0.1361, wR2 = 0.1806

Crystallographic data of [(*rac*)-**2**·**G5**] complex

The mixture of stereoisomers of [7_2]-pyrido-allenophane **2** was dissolved in a mixture of heptane, MTBE and methanol (1.6:0.4:1). Salicylic acid **G5** (1 eq) was added. Then, the solution was allowed to concentrate at room temperature for 2 days, leading to the growth of crystals of the [(P_2)/(M_2)-**2·G5**] complex.

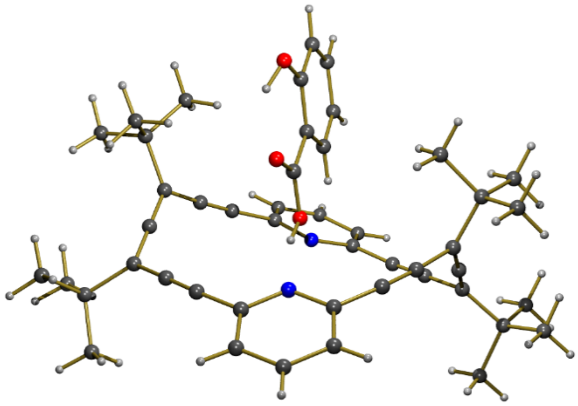

Figure S16. Crystal structure of [**2**·**G5**] obtained by X-ray diffraction.

Table S6. Crystal data and structure refinement for [**2**·**G3**]

Empirical formula	$C_{47}H_{48}N_2O_3$
Formula weight	688.87
Temperature	100.00 K
Wavelength	0.71073 Å
Crystal system	Triclinic
Space group	P-1
Unit cell dimensions	a = 10.9733(6) Å, α= 92.448(2)°
	b = 12.8296(6) Å, β= 106.534(2)°
	c = 16.2318(8) Å, γ = 95.862(2)°
Volume	2173.04(19) $Å^3$
Z	2
Density (calculated)	1.053 Mg/m^3
Absorption coefficient	0.065 mm^{-1}
F(000)	736
Crystal size	0.188 x 0.164 x 0.082 mm^3
Theta range for data collection	1.950 to 28.350°.
Index ranges	-14<=h<=14, -17<=k<=17, -21<=l<=21
Reflections collected	82090
Independent reflections	10846 [R(int) = 0.0384]
Completeness to theta = 25.242°	99.9 %

Absorption correction	Semi-empirical from equivalents
Max. and min. transmission	0.9685 and 0.9329
Refinement method	Full-matrix least-squares on F^2
Data / restraints / parameters	10846 / 2 / 549
Goodness-of-fit on F^2	1.033
Final R indices [I>2sigma(I)]	R1 = 0.0589, wR2 = 0.1680
R indices (all data)	R1 = 0.0751, wR2 = 0.1838

Crystallographic data of [(*rac*)-**2**]

The mixture of stereoisomers of [7_2]-pyrido-allenophane **2** was dissolved in a mixture of heptane and MTBE (8:2). The solution was then left to slowly concentrate at room temperature over 5 days, resulting in the formation of crystals identified as [(P_2)/(M_2)-**2**].

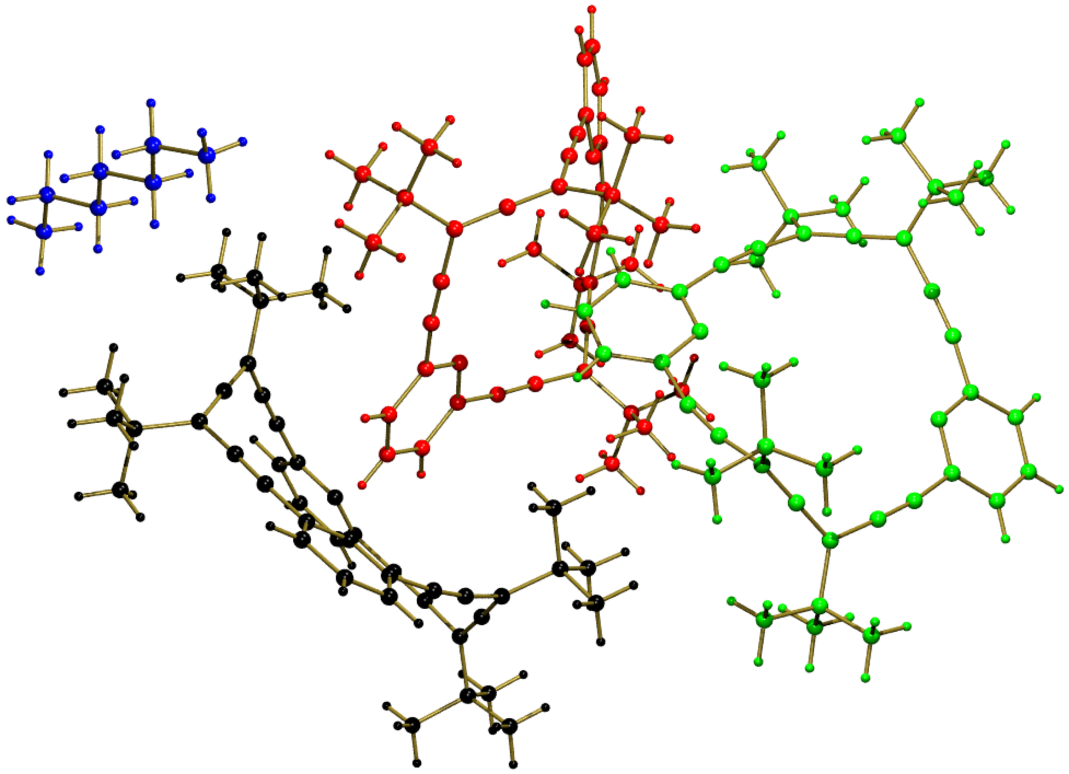

Figure S16B. The asymmetric unit contains three molecules of allenophane **2** and one molecule of heptane (solvent).

Table S6B. Crystal data and structure refinement for [(rac)-**2**].

Empirical formula	$C_{127}H_{142}N_6$
Formula weight	1752.44
Temperature	100.00 K
Wavelength	0.71073 Å
Crystal system	Monoclinic
Space group	P 1 21/n 1
Unit cell dimensions	a = 15.7484(6) Å, α= 90°
	b = 36.0872(11) Å, β= 95.5390(10)°
	c = 20.9893(7) Å, γ = 90°
Volume	11872.8(7) Å3
Z	4
Density (calculated)	0.980 Mg/m3
Absorption coefficient	0.056 mm-1
F(000)	3784
Crystal size	0.152 x 0.133 x 0.083 mm3
Theta range for data collection	1.915 to 26.372°.
Index ranges	-19<=h<=19, -45<=k<=45, -26<=l<=26
Reflections collected	231459
Independent reflections	24281 [R(int) = 0.0571]
Completeness to theta = 25.242°	100.0 %
Absorption correction	Semi-empirical from equivalents
Max. and min. transmission	0.7457 and 0.6488
Refinement method	Full-matrix least-squares on F2
Data / restraints / parameters	24281 / 0 / 1236
Goodness-of-fit on F2	1.025
Final R indices [I>2sigma(I)]	R1 = 0.0436, wR2 = 0.1122
R indices (all data)	R1 = 0.0558, wR2 = 0.1203

4. Titrations experiments

4.1. Allenophane 2

Catechol (**G1)**

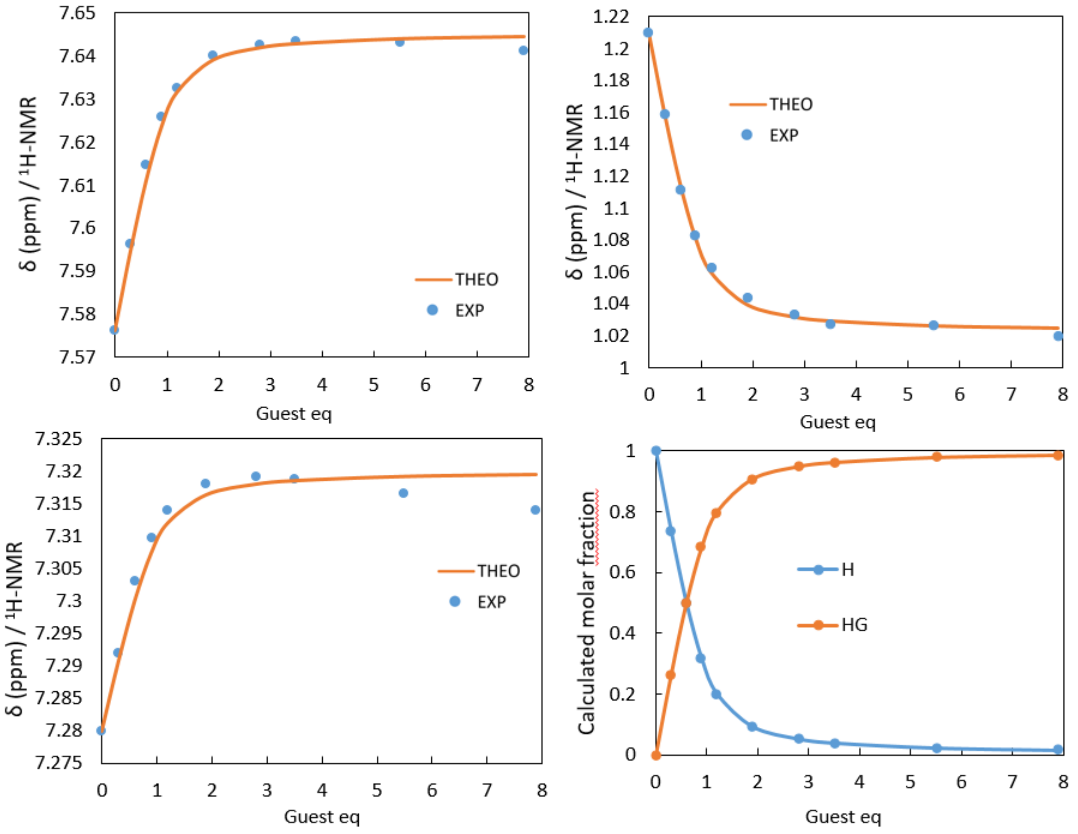

Figure S17. Mathematical fitting (THEO) of experimental data for the complexation of catechol (**G1)** by allenophane (*P*$_2$)-**2** to a 1:1 stoichiometry (H:G), along with the corresponding species balance.

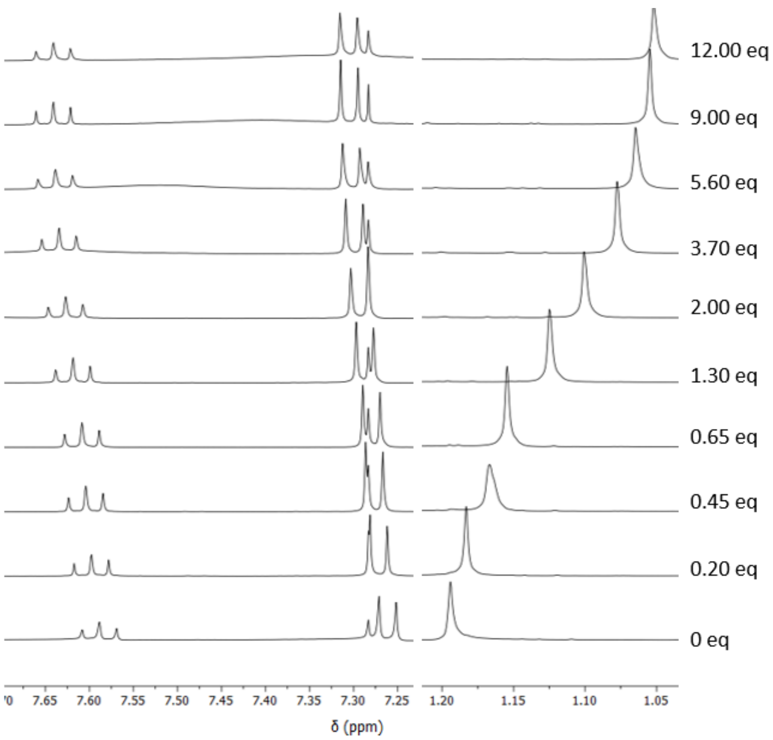

Figure S18. Segment of the ^{1}H-NMR titration of (*P*$_2$)-**2** (0.018 M) in $CDCl_3$:MeOH 5% with catechol (**G1**) (ranging from 0 to 0.72 M).

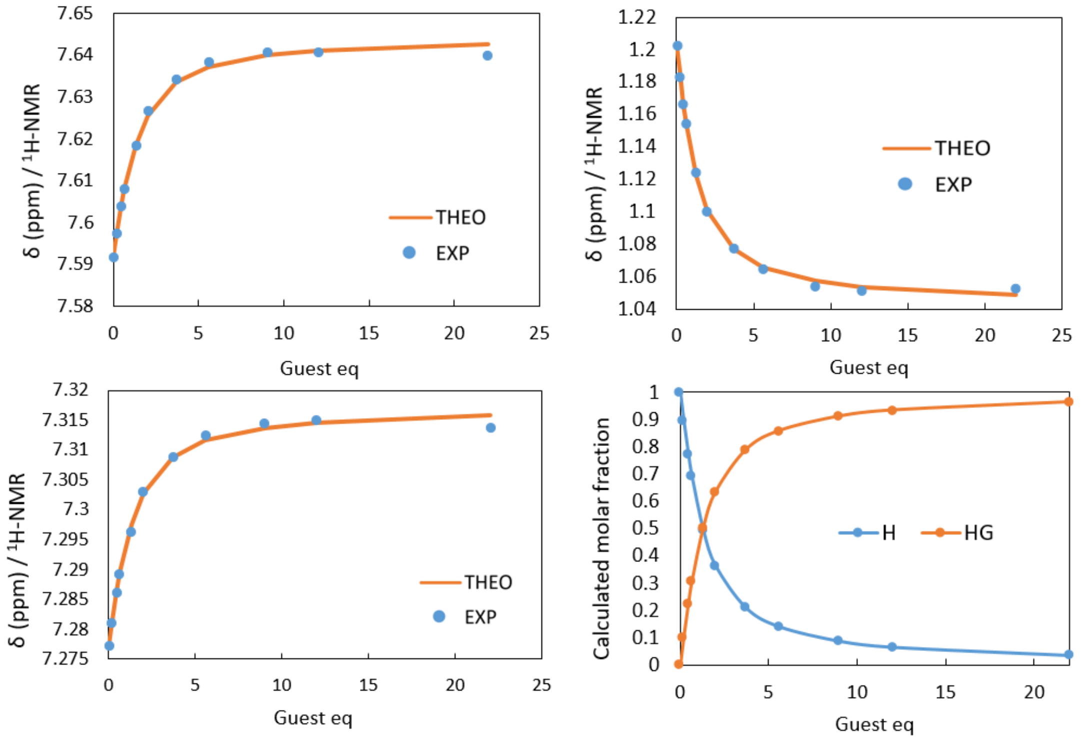

Figure S19. Mathematical fitting (THEO) of experimental data presented in **Figure S18** for the complexation of catechol (**G1**) by allenophane (*P*$_2$)-**2** to a 1:1 stoichiometry (H:G), along with the corresponding species balance.

Caffeic acid phenethyl ester CAPE (**G2**)

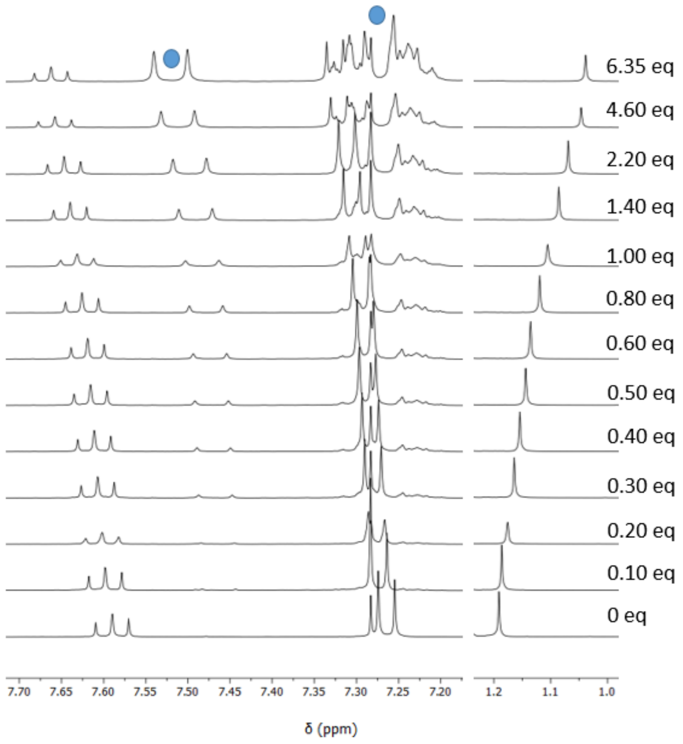

Figure S20. Segment of the ^{1}H-NMR titration of (P_2)-**2** (0.018 M) in $CDCl_3$:MeOH 5% with CAPE **G2**(ranging from 0 to 0.11 M). Blue dots indicate signals coming from (**G2**).

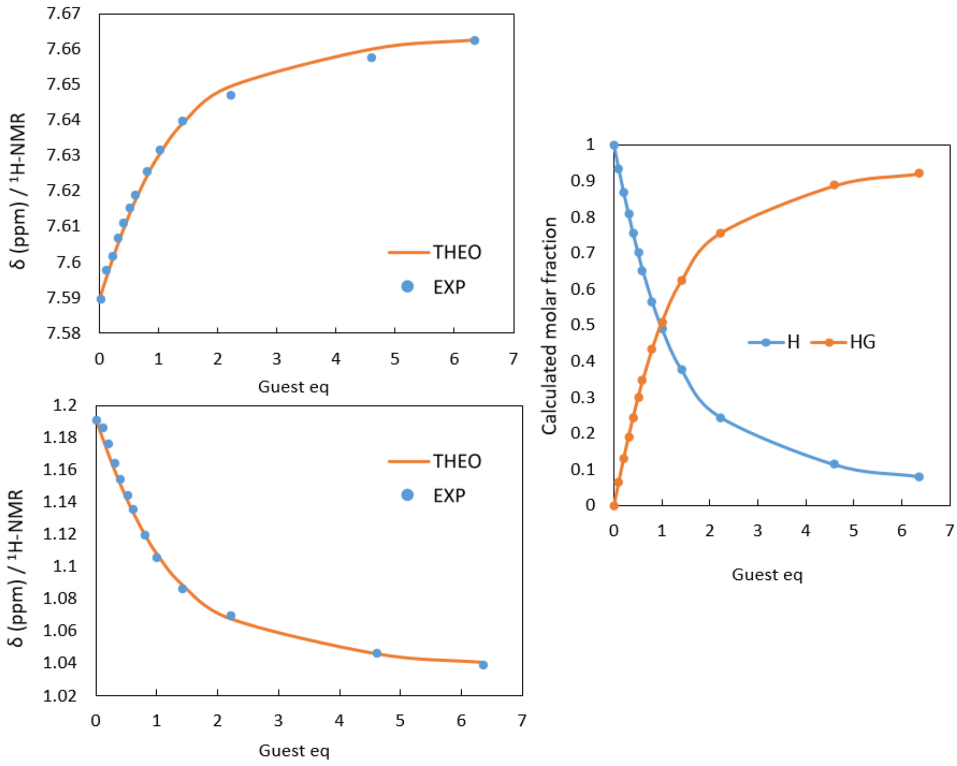

Figure S21. Mathematical fitting (THEO) of experimental data presented in **Figure S20** for the complexation of CAPE (**G2**) by allenophane (P_2)-**2** to a 1:1 stoichiometry (H:G), along with the corresponding species balance.

Resorcinol (**G3)**

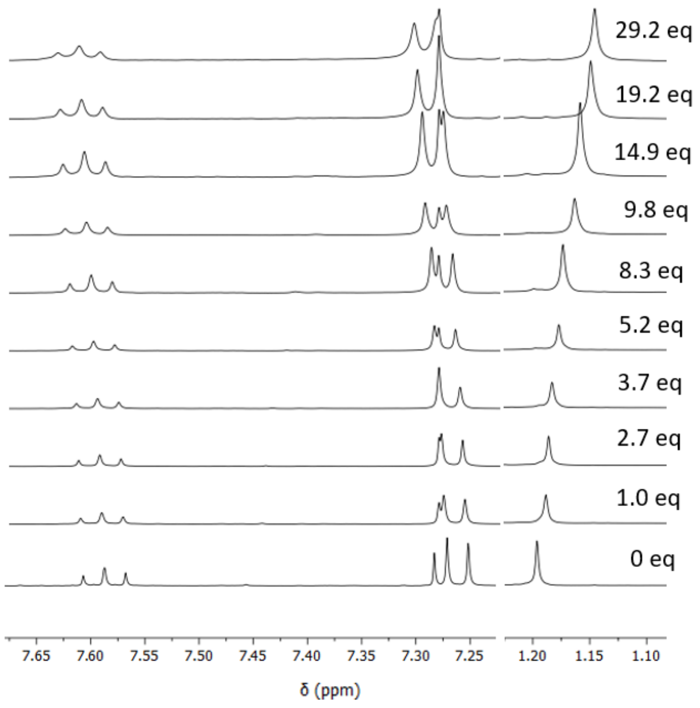

Figure S22. Segment of the ^{1}H-NMR titration of (P_2)-**2** (0.018 M) in $CDCl_3$:MeOH 5% with resorcinol (**G3**) (ranging from 0 to 0.61M).

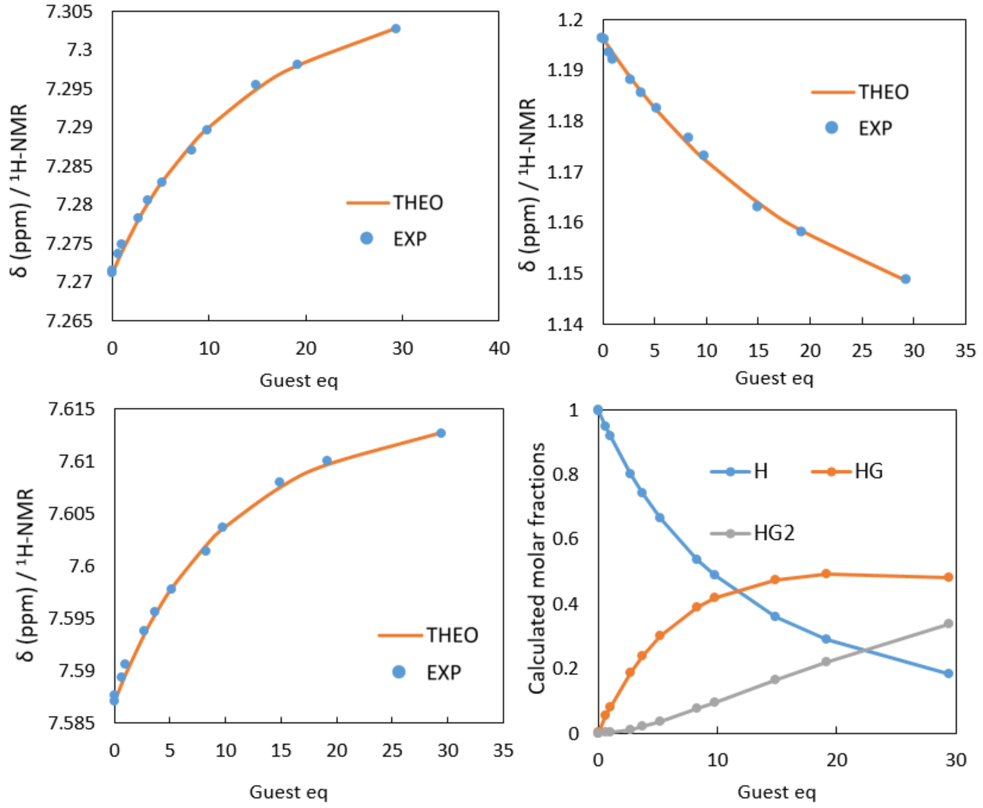

Figure S23. Mathematical fitting (THEO) of experimental data presented in **Figure S22** for the complexation of resorcinol (**G3**) by allenophane (P_2)-**2** to a 1:2 stoichiometry (H:G), along with the corresponding species balance.

Salicylic acid (**G5**)

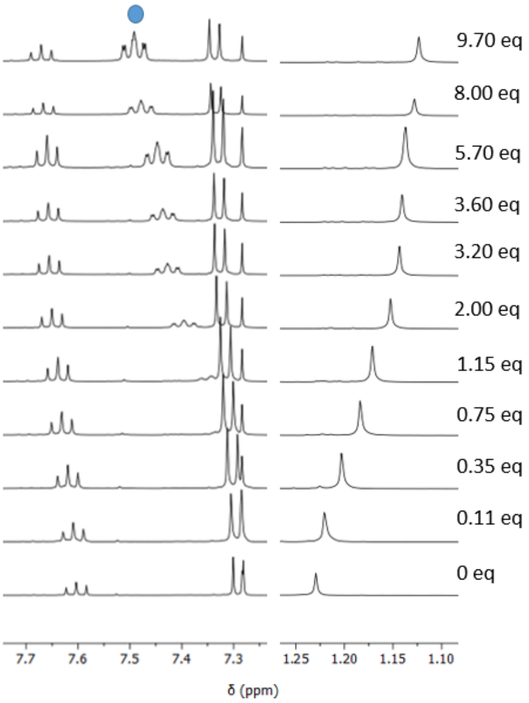

Figure S24. Segment of the ^{1}H-NMR titration of (P_2)-**2** (0.018 M) in $CDCl_3$ with salicylic acid (**G5**) (ranging from 0 to 0.29 M). Blue dot indicates a signal coming from (**G5**).

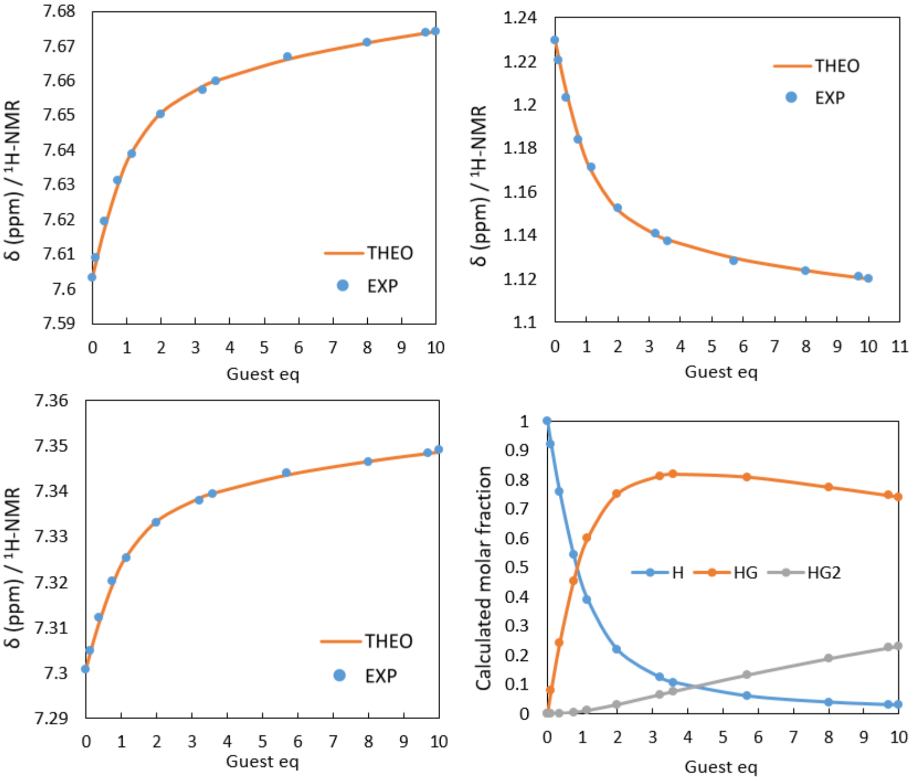

Figure S25. Mathematical fitting (THEO) of experimental data presented in **Figure S24** for the complexation of salicylic acid (**G5**) by allenophane (P_2)-**2** to a 1:2 stoichiometry (H:G), along with the corresponding species balance.

2-Hydroxyisobutyric acid (**G6**)

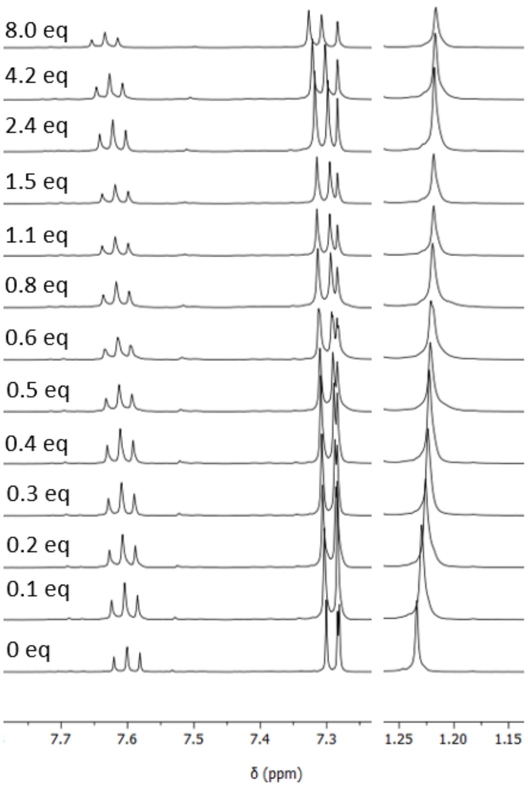

Figure S26. Segment of the ^{1}H-NMR titration of (P_2)-**2** (0.018 M) in $CDCl_3$ with 2-hydroxyisobutyric acid (**G6**) (ranging from 0 to 0.12 M).

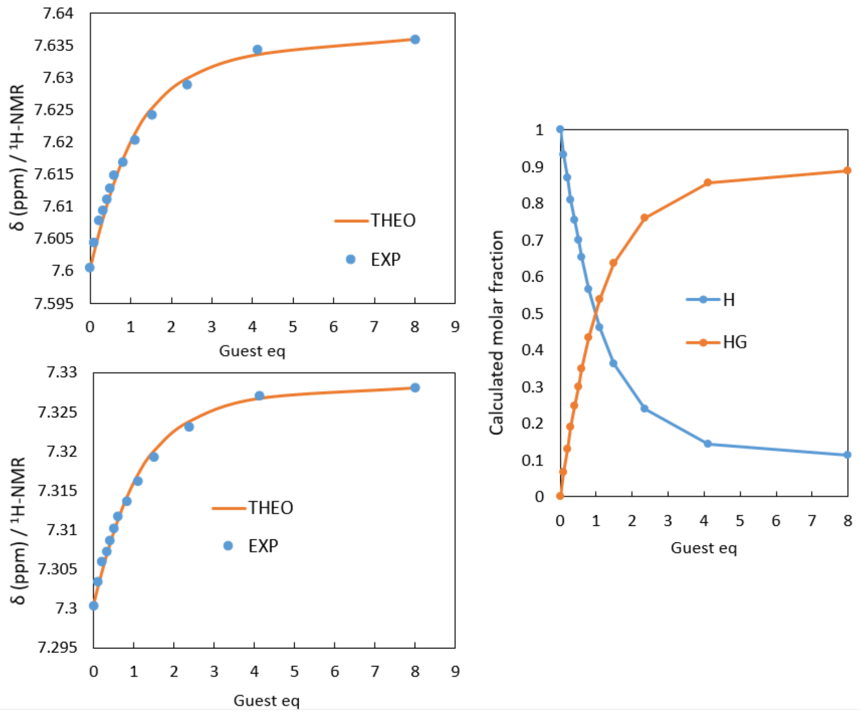

Figure S27. Mathematical fitting (THEO) of experimental data presented in **Figure S26** for the complexation of 2-hydroxyisobutyric acid (**G6**) by allenophane (P_2)-**2** to a 1:1 stoichiometry (H:G), along with the corresponding species balance.

(*S*)-2-Hydroxy-3-methylbutyric acid (**G7**)

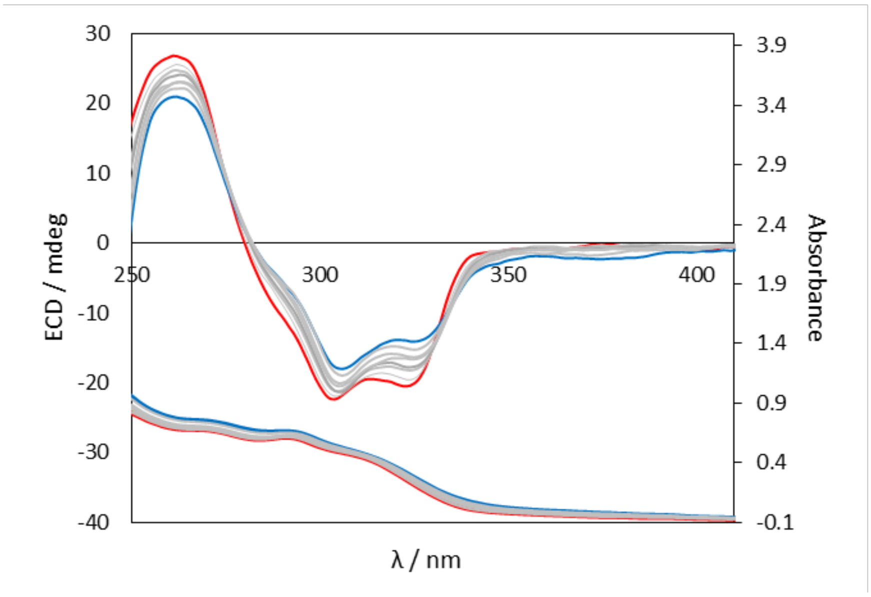

Figure S28. Change of the ECD (top) and UV/Vis spectra (bottom) of (P_2)-**2** (1.5 x 10^{-5}M, $CHCl_3$) upon addition of (S)-2-Hydroxy-3-methylbutyric acid (**G7**) (acid concentration in the cuvette ranging from 0 to 0.13 M). Red line: Original signal before adding (*S*)-**G7**. Blue line: ECD signal at the final saturation value of (*S*)-**G7**. Grey lines: Changes with increasing amounts of (*S*)-**G7**.

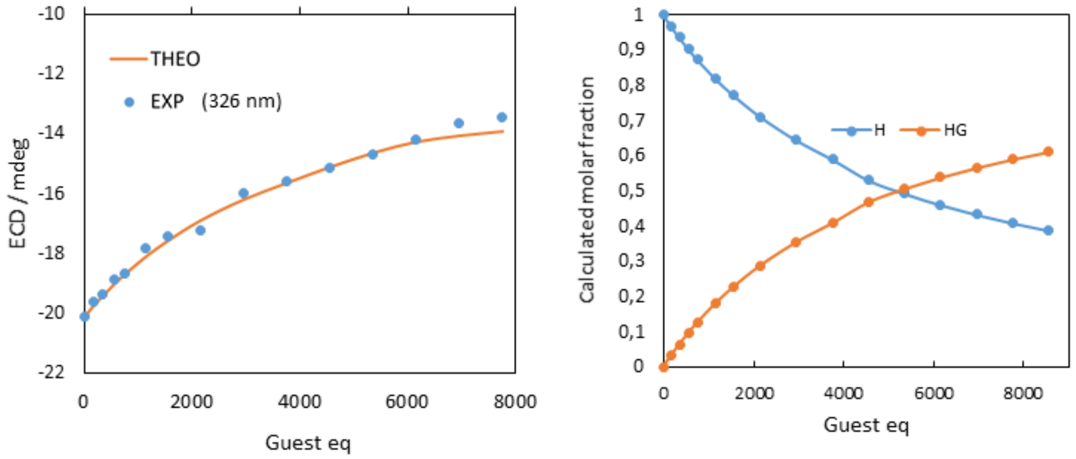

Figure S29. Mathematical fitting (THEO) of experimental data presented in **Figure S27** for the complexation of (*S*)-2-Hydroxy-3-methylbutyric acid (**G7**) by allenophane (P_2)-**2** to a 1:1 stoichiometry (H:G), along with the corresponding species balance.

(*R*)-2-Hydroxy-3-methylbutyric acid (**G7**)

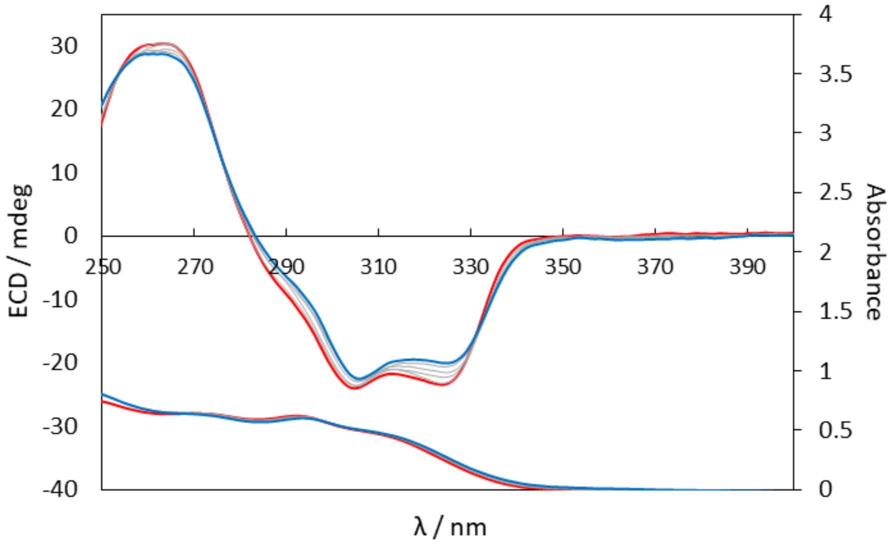

Figure S30. Change of the ECD (top) and UV/Vis spectra (bottom) of (P_2)-**2** (1.5 x 10^{-5}M, $CHCl_3$) upon addition of (*R*)-2-Hydroxy-3-methylbutyric acid (**G7**) (acid concentration in the cuvette ranging from 0 to 0.04 M). Red line: Original signal before adding (*R*)-**G7**. Blue line: ECD signal at the final saturation value of (*R*)-**G7**. Grey lines: Changes with increasing amounts of (*R*)-**G7**.

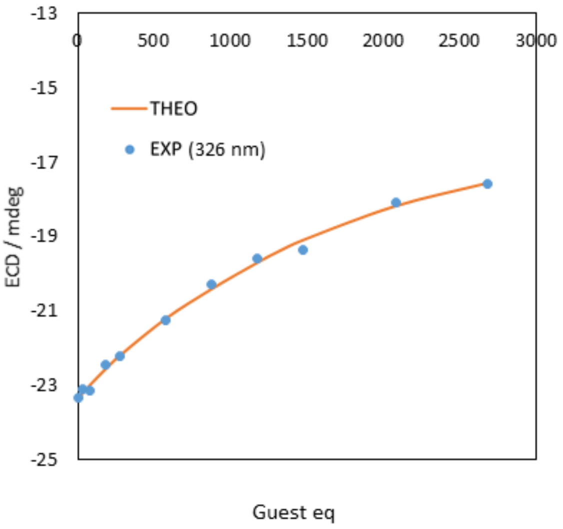

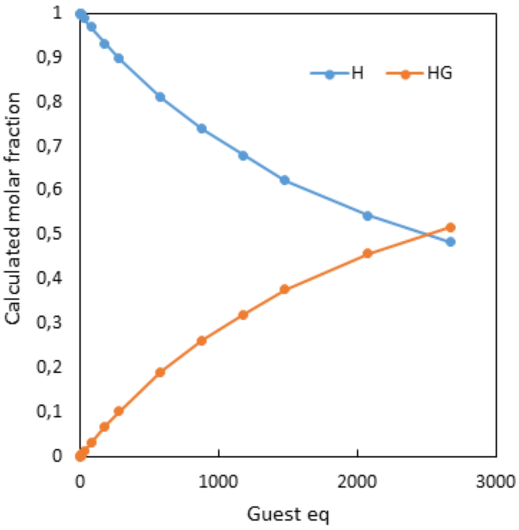

Figure S31. Mathematical fitting (THEO) of experimental data presented in **Figure S30** for the complexation of (*R*)-2-Hydroxy-3-methylbutyric acid **(G7)** by allenophane (P_2)-**2** to a 1:1 stoichiometry (H:G), along with the corresponding species balance.

4.2. Allenophane 3

Tetramethylammonium chloride (**G13**)

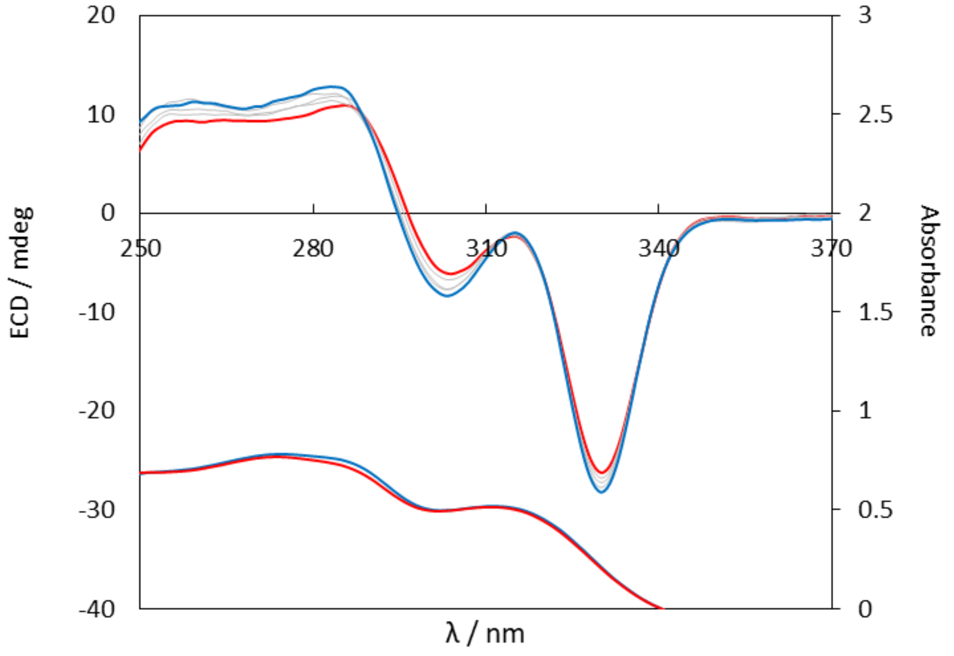

Figure S32. Titration course for (P_3)-**3** (1.5×10^{-5} M) with tetramethylammonium chloride (**G13**) (ranging from 0 to 5×10^{-3} M) in $CHCl_3$:EtOH 5%. Top ECD and bottom UV/Vis spectrum. The red lines represent the initial spectral bands. The blue lines represent the end of the titration.

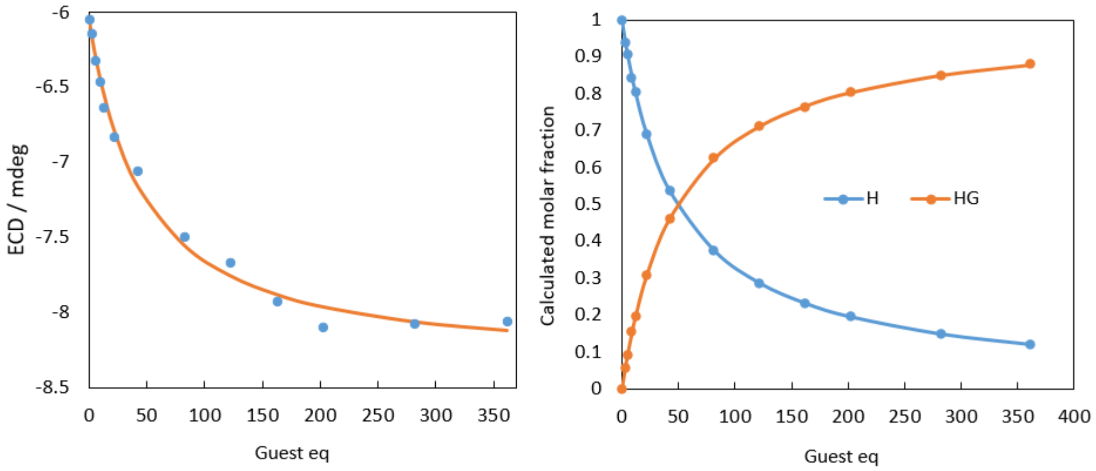

Figure S33. Mathematical fitting (THEO) of experimental data presented in Figure **S32** for the complexation of tetramethylammonium chloride (**G13**) by allenophane (P_3)-**3** to a 1:1 stoichiometry (H:G), along with the corresponding species balance.

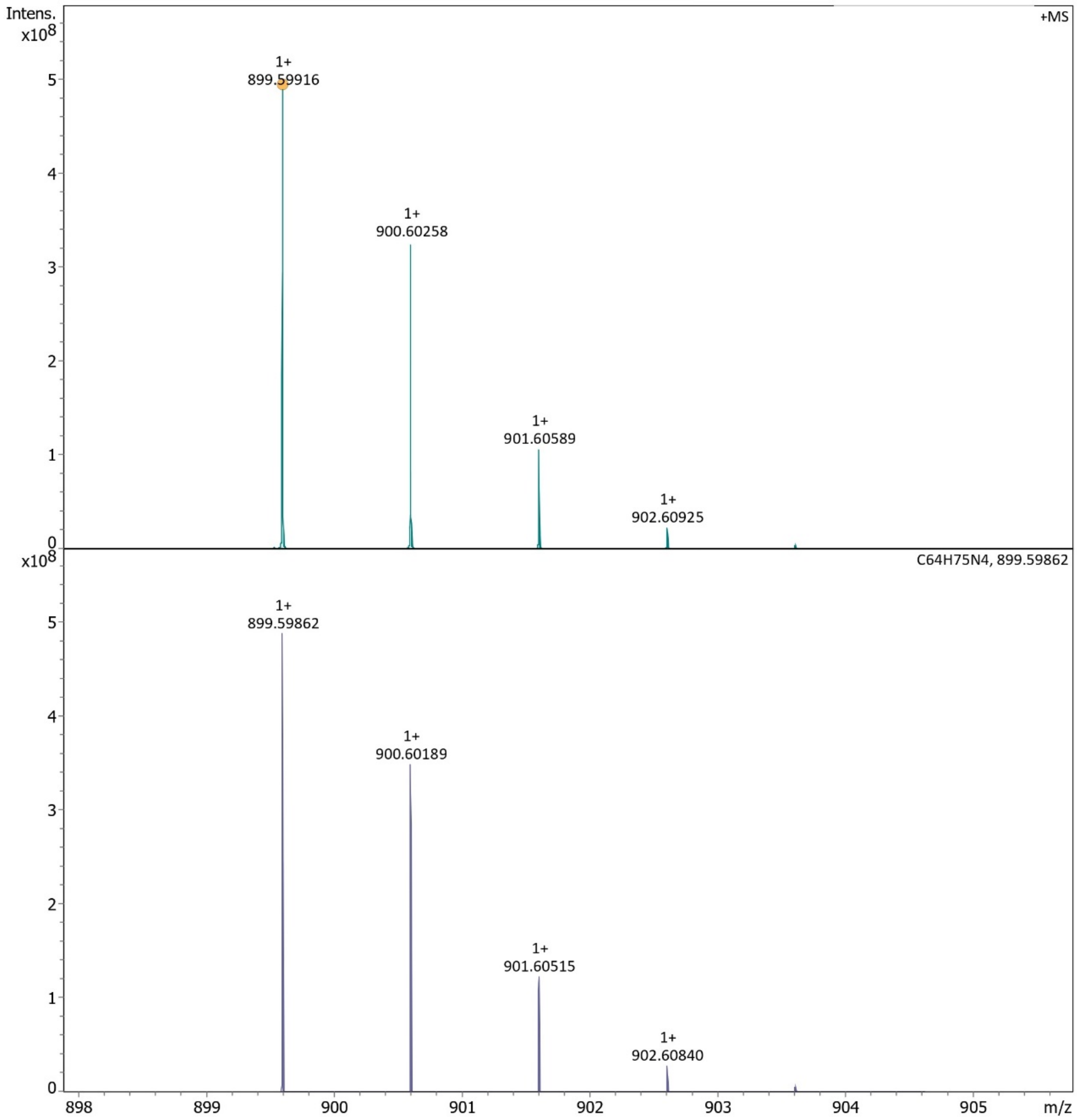

Figure S34. HRMS-ESI m/z and isotopic pattern calcd for $C_{64}H_{75}N_4^+$ 899.5986 (grey); found 899.5992 (green) [**3**·(**G13**)]$^+$.

Cetrimonium chloride (**G14**)

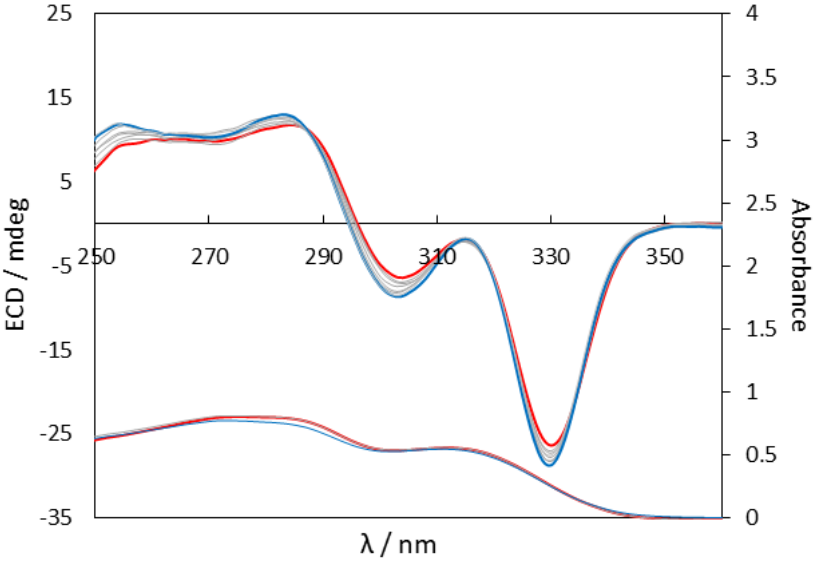

Figure S35. Titration course for (P_3)-**3** (1.5×10^{-5} M) with cetrimonium chloride (**G14**) (ranging from 0 to 6×10^{-3} M) in $CHCl_3$:EtOH 5%. Top ECD and bottom UV/Vis spectrum. The red lines represent the initial spectral bands. The blue lines represent the end of the titration.

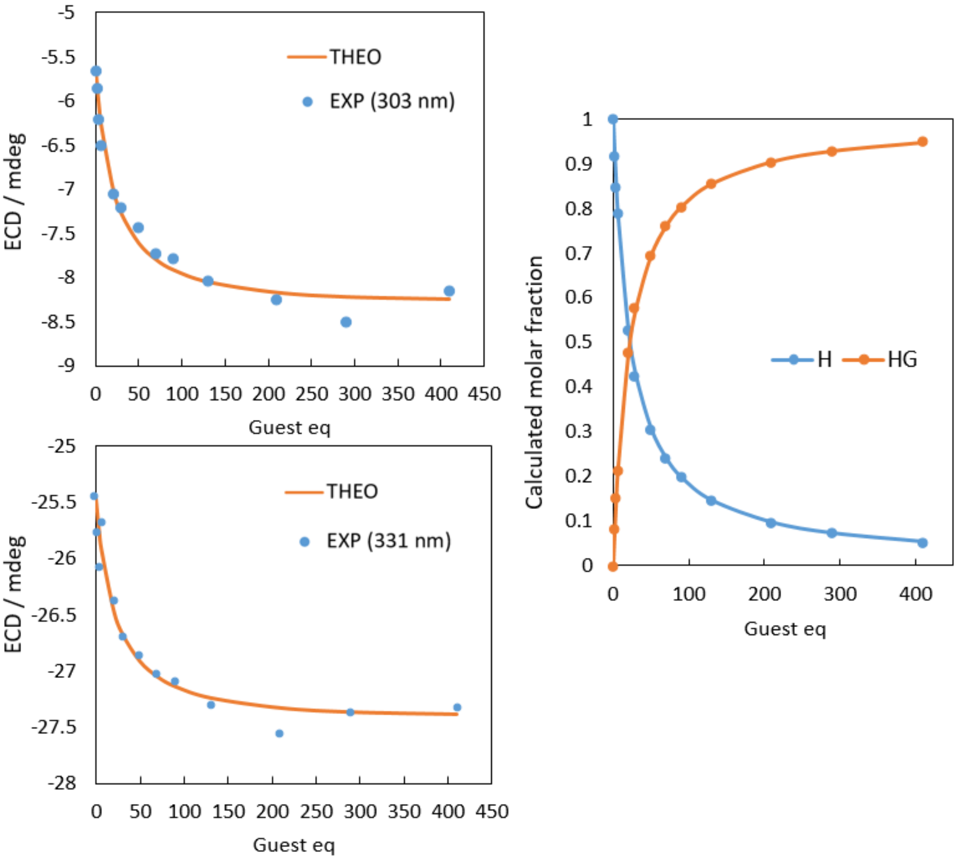

Figure S36. Mathematical fitting (THEO) of experimental data presented in **Figure S35** for the complexation of cetrimonium chloride (**G14**) by allenophane (P_3)-**3** to a 1:1 stoichiometry (H:G), along with the corresponding species balance.

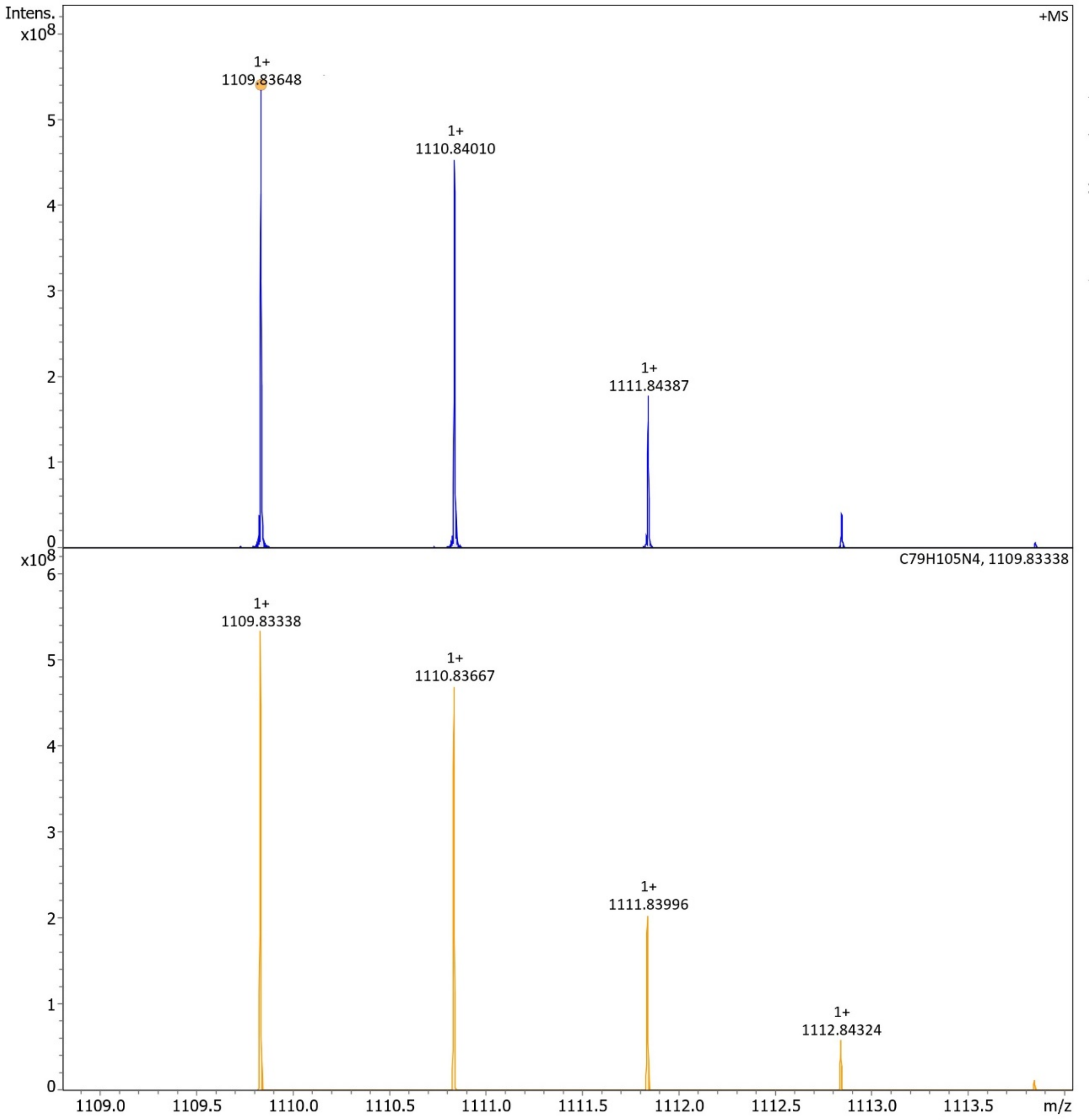

Figure S37. HRMS-ESI m/z and isotopic pattern calcd for $C_{79}H_{105}N_4^+$ 1109.8334 (yellow); found 1109.8365 (blue) [**3**·(**G14**)]$^+$.

Didecyldimethylammonium chloride (**G15**)

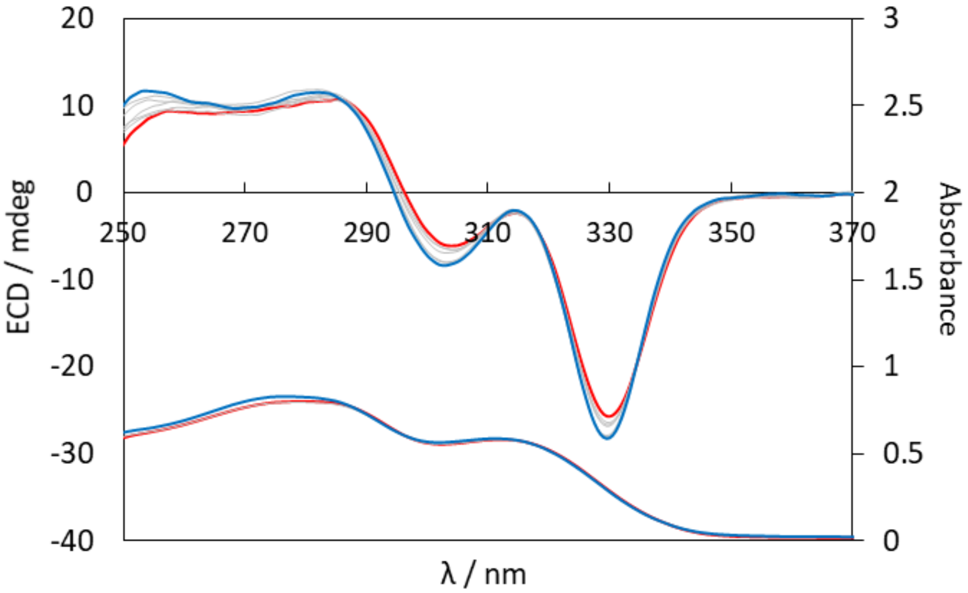

Figure S38. Titration course for (P_3)-**3** (1.5×10^{-5} M) with didecyldimethylammonium chloride (**G15**) (ranging from 0 to 3.0×10^{-3} M) in $CHCl_3$:EtOH 5%. Top ECD and bottom UV/Vis spectrum. The red lines represent the initial spectral bands. The blue lines represent the end of the titration.

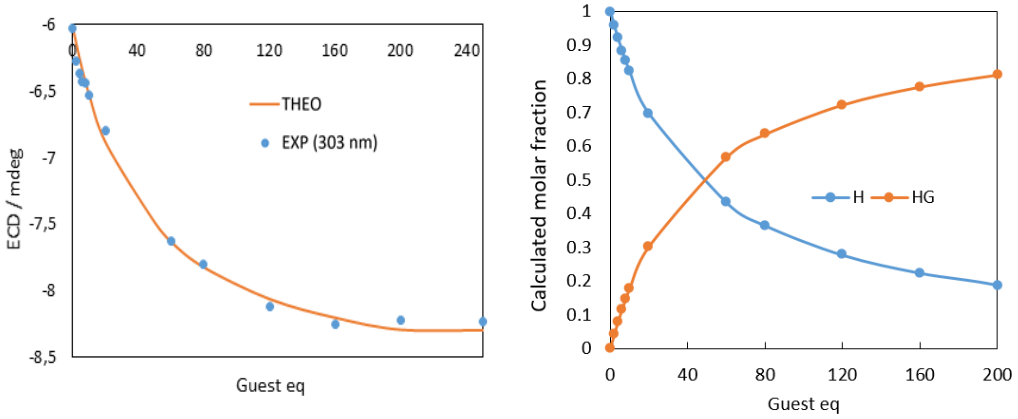

Figure S39. Mathematical fitting (THEO) of experimental data presented in **Figure S38** for the complexation of didecyldimethylammonium chloride (**G15**) by allenophane (P_3)-**3** to a 1:1 stoichiometry (H:G), along with the corresponding species balance.

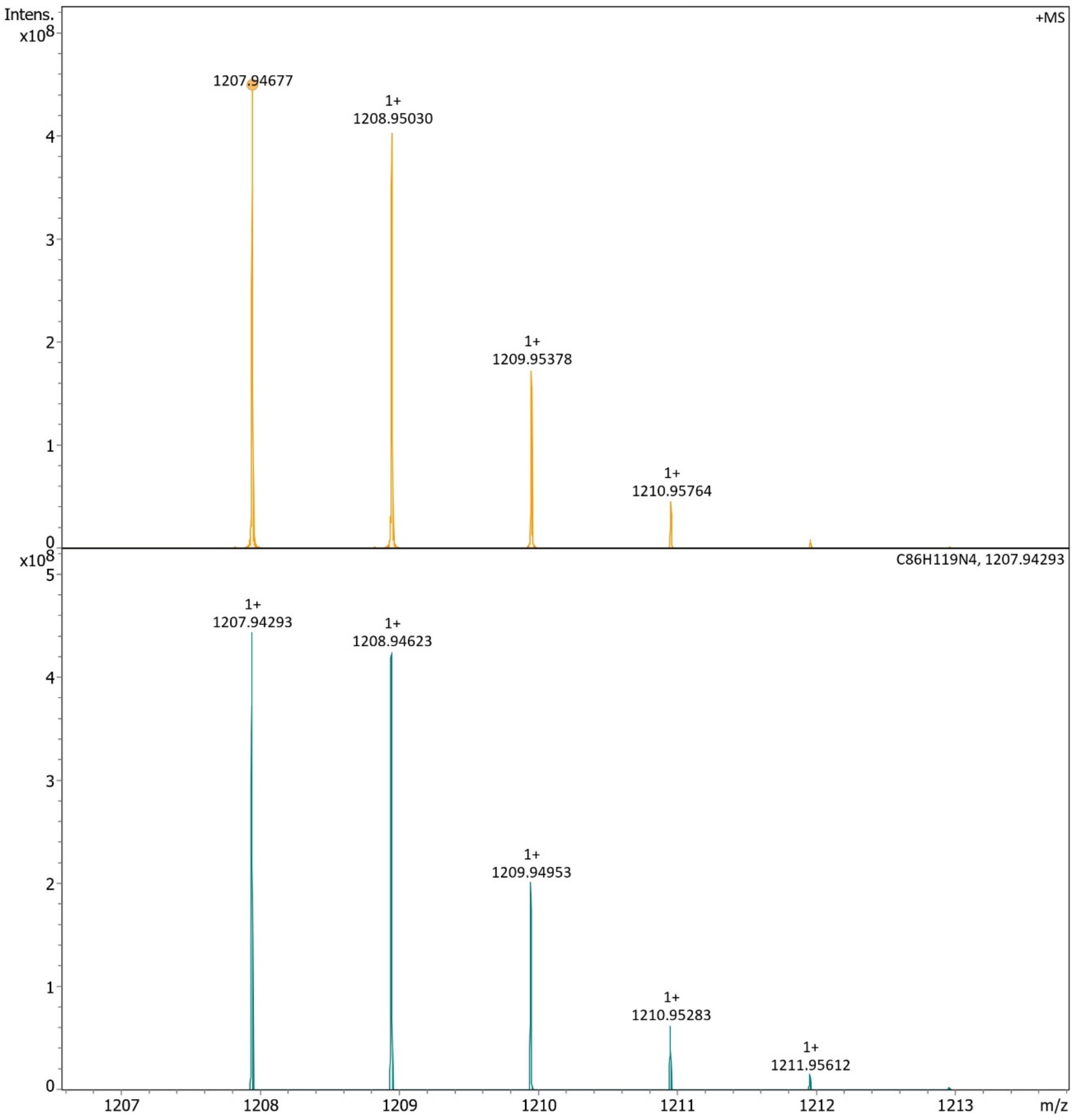

Figure S40. HRMS-ESI m/z and isotopic pattern calcd for $C_{86}H_{119}N_4^+$ 1207.9429 (green); found 1207.9468 (yellow) [**3**·(**G15**)]$^+$.

Choline 2-hydroxyisobutyrate (**G16**)

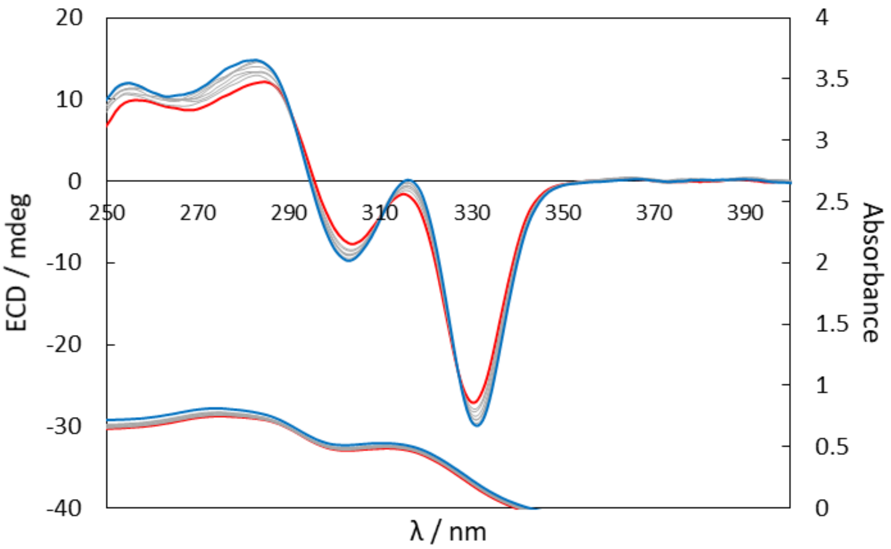

Figure S41. Titration course for (P_3)-**3** (1.5×10^{-5} M) with choline 2-hydroxyisobutyrate (**G16**) (ranging from 0 to 4×10^{-3} M) in $CHCl_3$:EtOH 5%. Top ECD and bottom UV/Vis spectrum. The red lines represent the initial spectral bands. The blue lines represent the end of the titration.

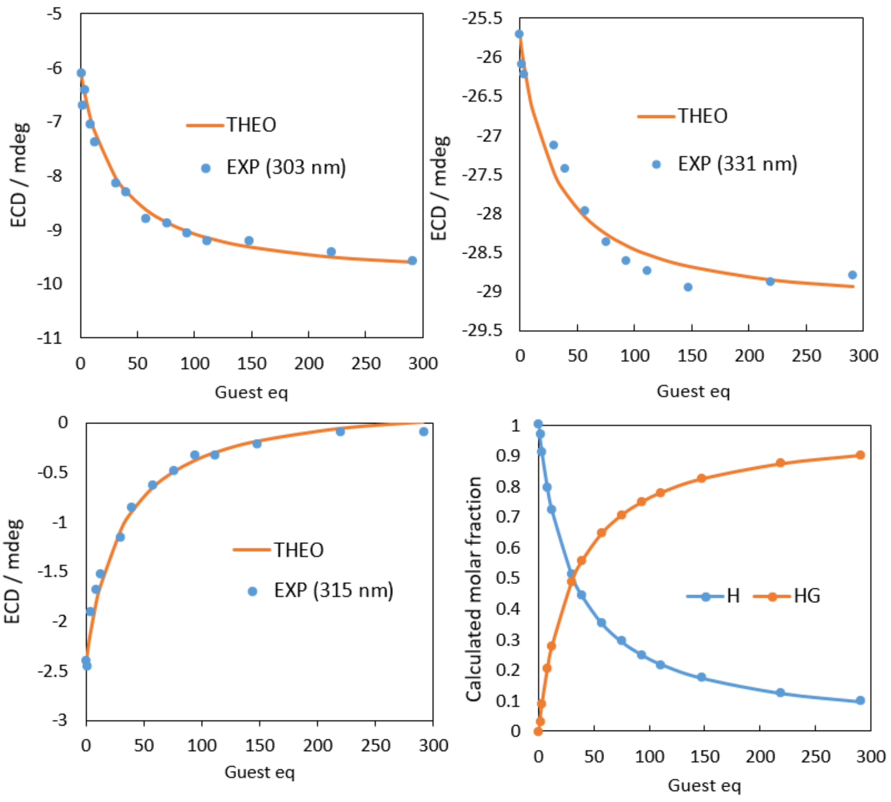

Figure S42. Mathematical fitting (THEO) of experimental data presented in **Figure S41** for the complexation of choline 2-hydroxyisobutyrate (**G16**) by allenophane (P_3)-**3** to a 1:1 stoichiometry (H:G), along with the corresponding species balance.

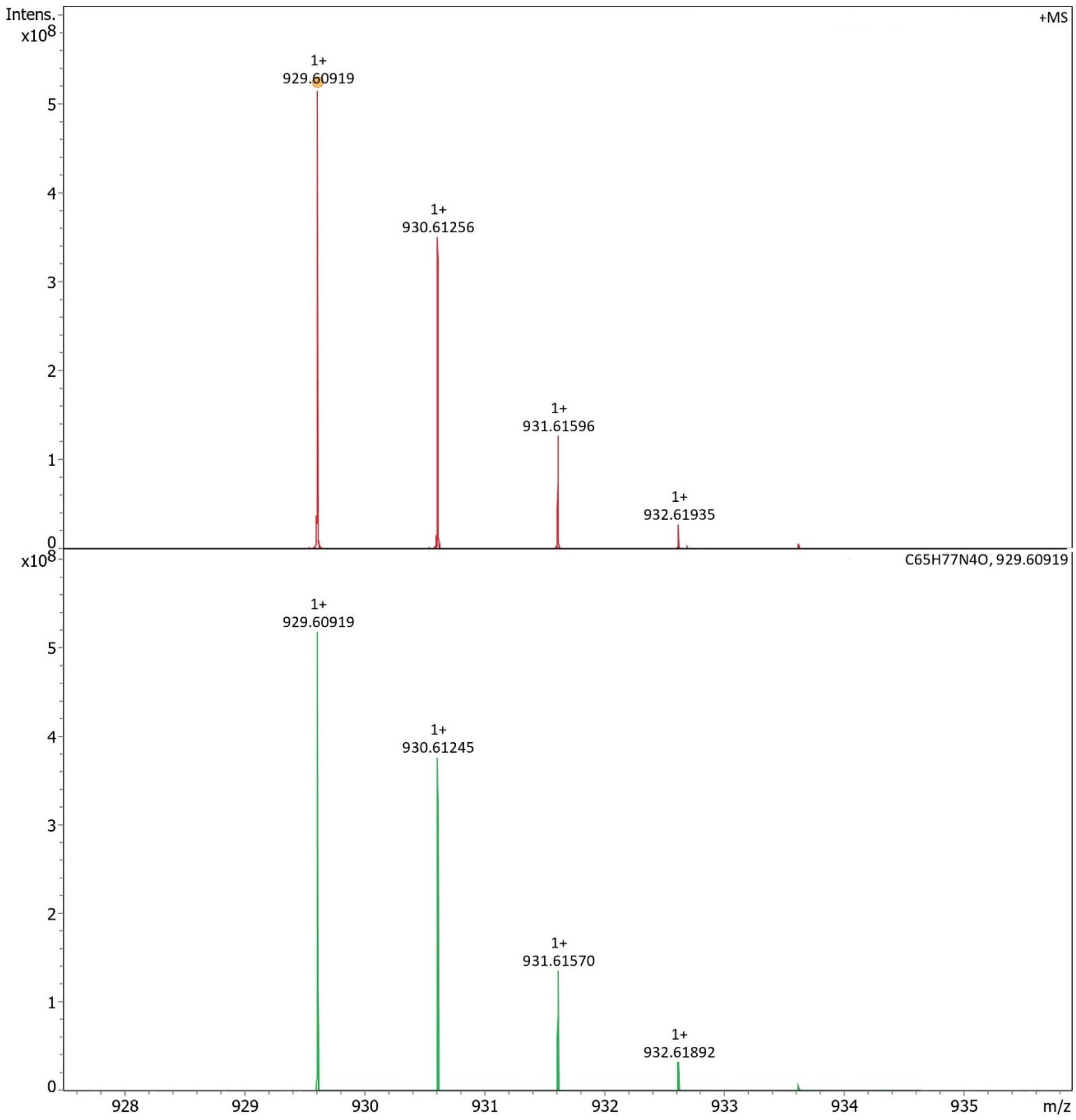

Figure S43. HRMS-ESI m/z and isotopic pattern calcd for $C_{65}H_{77}N_4O^+$ 929.6092 (green); found 929.6092 (red) [**3**·(**G16**)]$^+$.

(*S*)-4-(hexyloxy)-2-hydroxy-*N*,*N*,*N*-trimethyl-4-oxobutan-1-aminium iodide (L-**G17**)

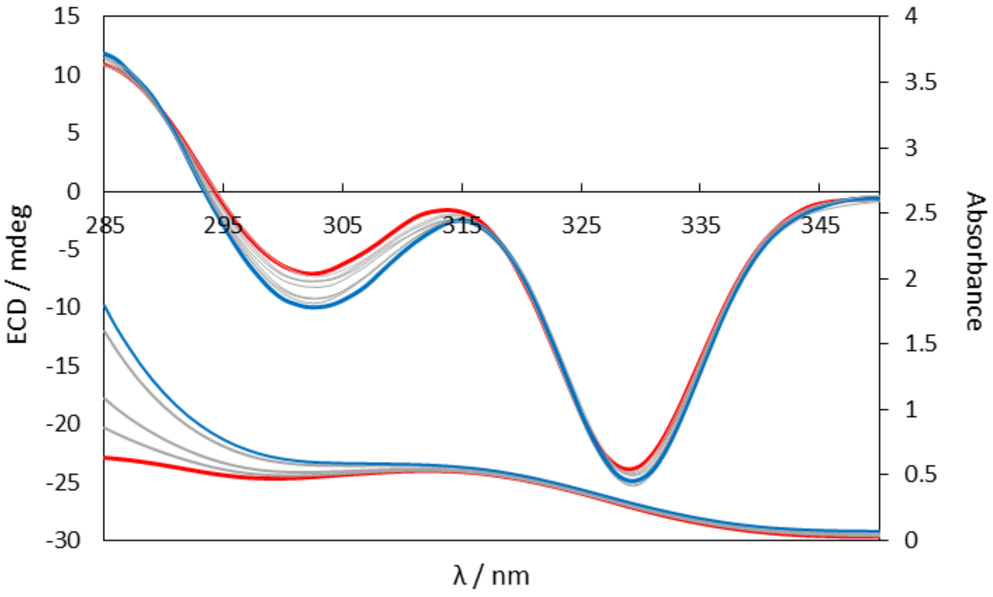

Figure S44. Titration course for (P_3)-**3** (1.5×10^{-5} M) with L-**G17** (ranging from 0 to 2×10^{-2} M) in $CHCl_3$:EtOH 5%. Top ECD and bottom UV/Vis spectrum. The red lines represent the initial spectral bands. The blue lines represent the end of the titration.

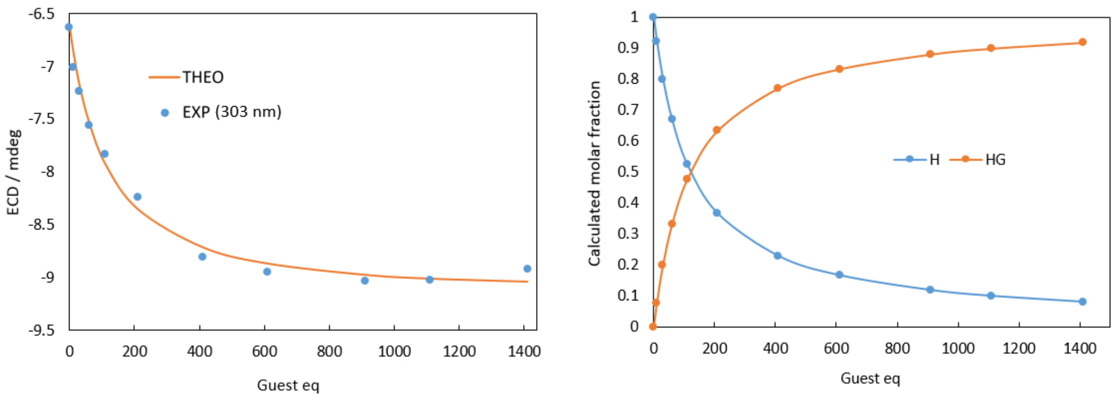

Figure S45. Mathematical fitting (THEO) of experimental data presented in Figure S44 for the complexation of L-**G17** by allenophane (P_3)-**3** to a 1:1 stoichiometry (H:G), along with the corresponding species balance.

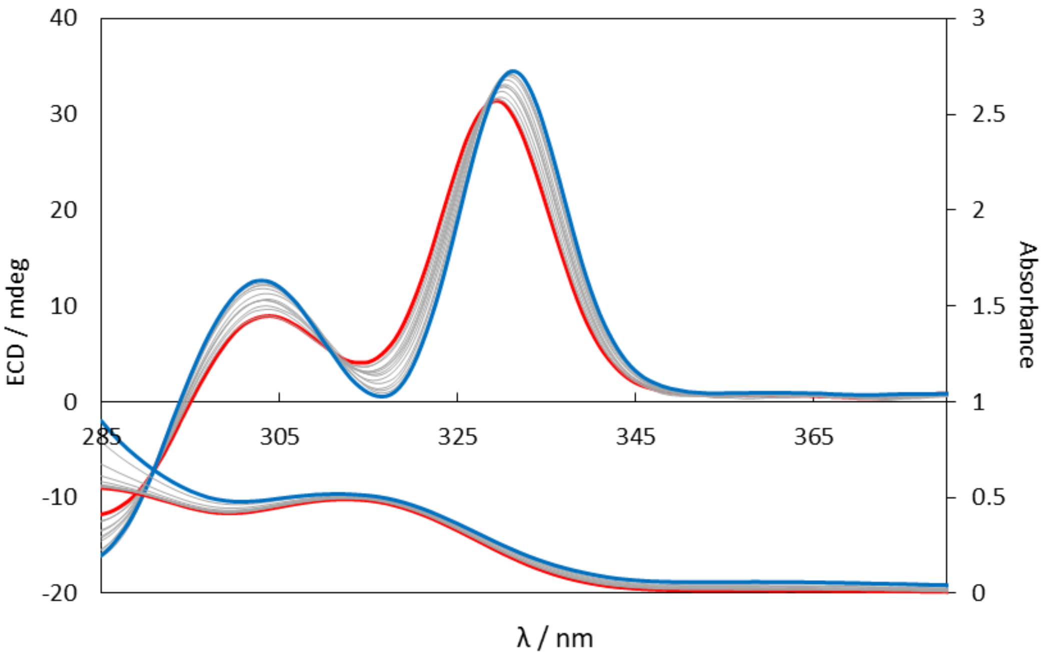

Figure S46. Titration course for (M_3)-**3** (1.5×10^{-5} M) with L-**G17** (ranging from 0 to 6×10^{-3} M) in $CHCl_3$:EtOH 5%. Top ECD and bottom UV/Vis spectrum. The red lines represent the initial spectral bands. The blue lines represent the end of the titration.

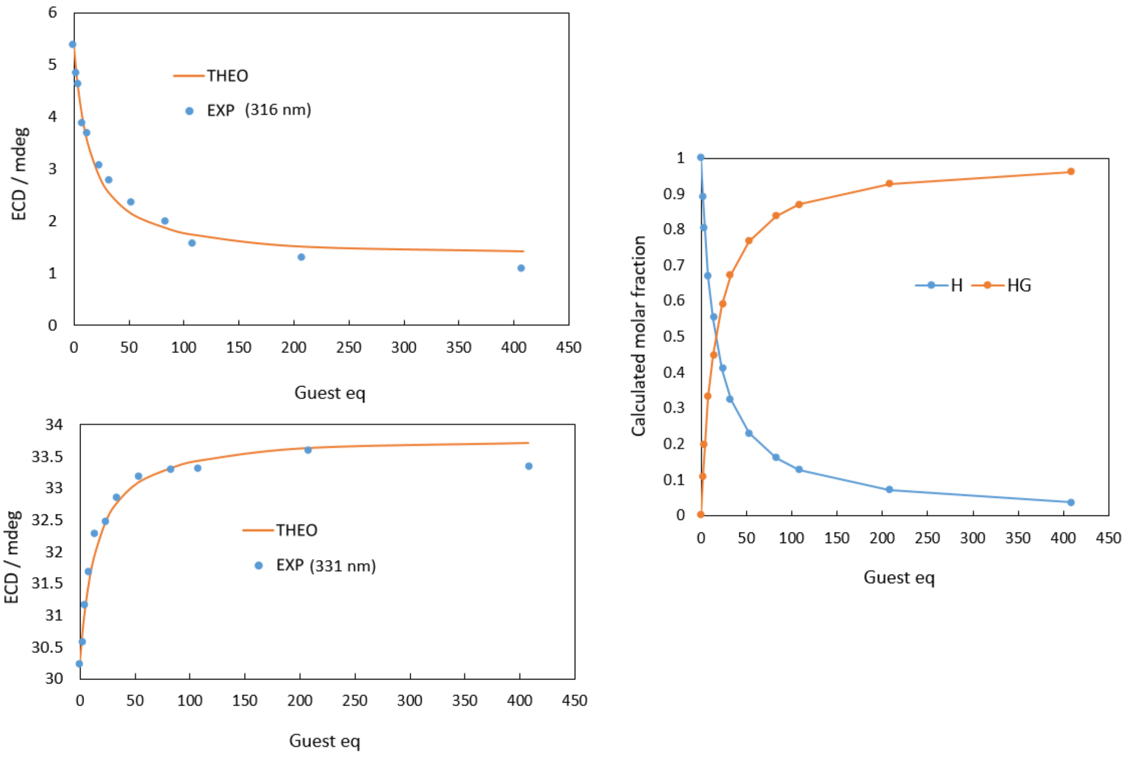

Figure S47. Mathematical fitting (THEO) of experimental data presented in Figure S46 for the complexation of L-**G17** by allenophane (M_3)-**3** to a 1:1 stoichiometry (H:G), along with the corresponding species balance.

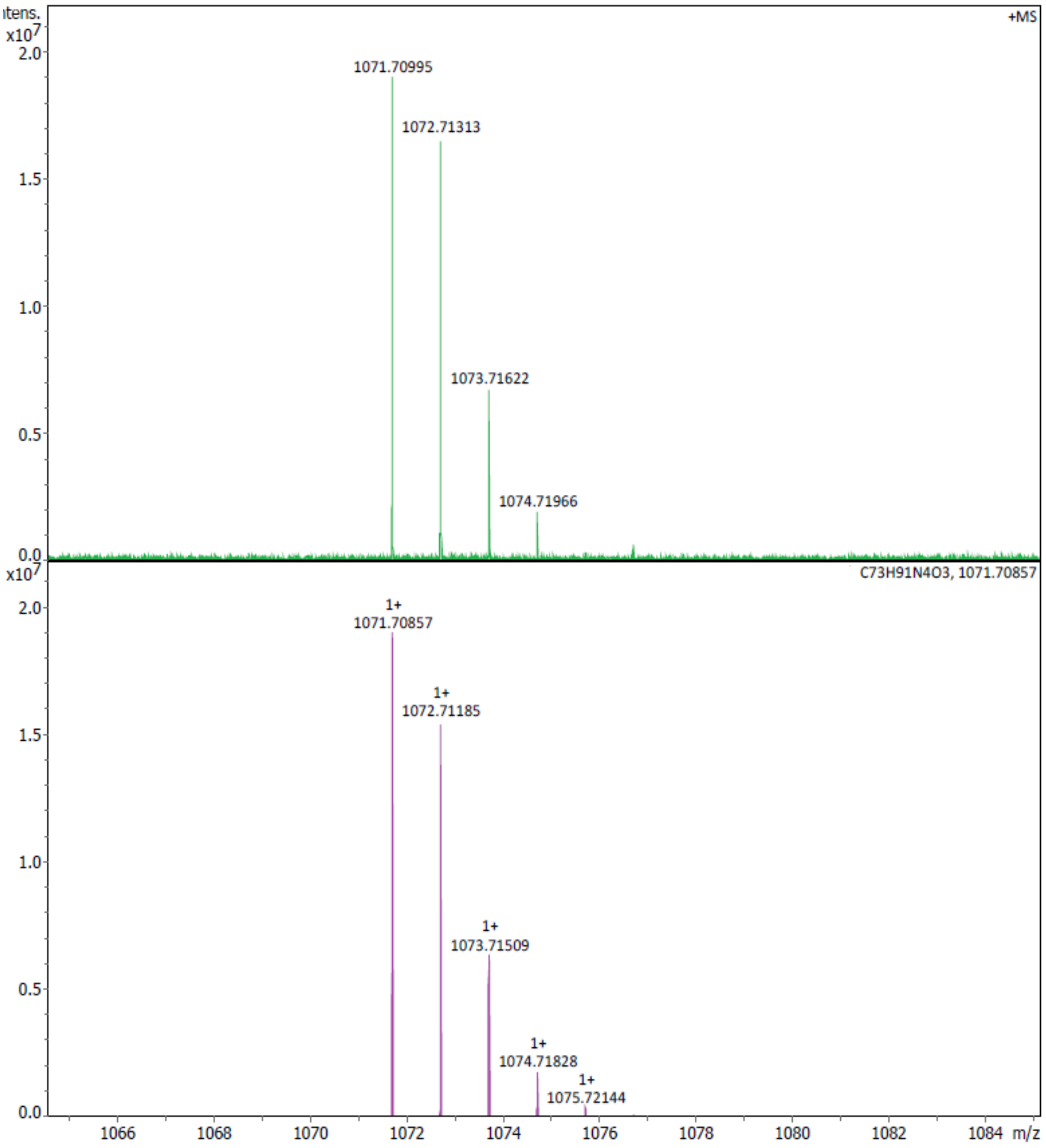

Figure S48. HRMS-ESI m/z and isotopic pattern calcd for $C_{73}H_{91}N_4O_3^+$ 1071.7086 (purple); found 1071.7099 (green) [**3**·**G17**]$^+$.

5. Theoretical Calculations

5.1. Conformers of (P_3)-**3** and (P_4)-**4**

(P_3)-**3**

Through DFT computational calculations, two conformers with nearly identical energy were identified for the allenophane (P_3)-**3**: one adopting a crown-shaped conformation **A** with C_3 symmetry (**Figure S49**), and the other adopting a helical conformation **B** with C_2 symmetry (**Figure S50**).

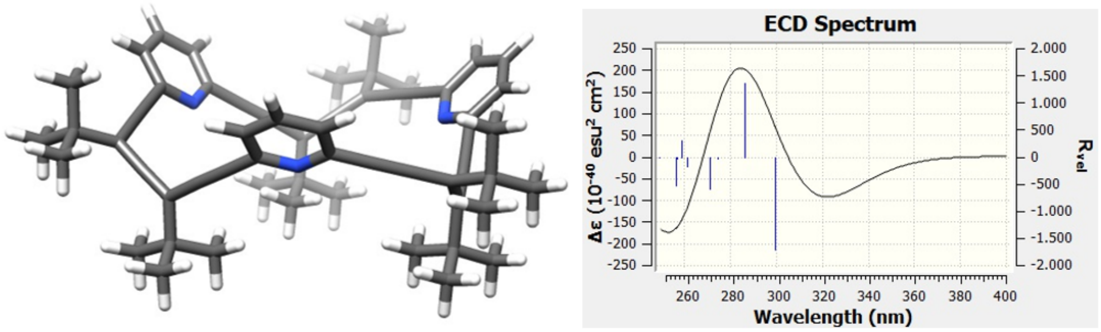

(P_3)-**3** Conformer **A** (C_3, 0.0)

Figure S49. Optimized structure of (P_3)-**3** conformer A and ECD calculated spectrum. In parentheses, the point group symmetry and the relative energy in kcal/mol for this conformer are indicated.

C	-3.17026400	-5.99354600	-2.62713900
C	-4.09261100	-5.01341400	-2.29842100
C	-3.76040400	-4.09148100	-1.29873500
N	-2.59506200	-4.11924500	-0.64153800
C	-1.71096800	-5.06725800	-0.97168300
C	-1.95591500	-6.02707300	-1.96023300
C	-0.46291000	-5.06569600	-0.26217600
C	0.59740400	-5.05466300	0.31549800
C	1.83256500	-5.00956900	1.04360000
C	4.08113600	-4.92868200	-0.30878000
C	4.57580100	-3.62940600	-0.65917100
C	4.97879300	-2.52759800	-0.94686700
C	6.19847400	1.31991100	-1.96063100
C	6.77651500	0.25150700	-2.62765100
C	6.38864800	-1.03733400	-2.29916400
C	5.42397200	-1.21061000	-1.29963300
N	4.86543700	-0.18752900	-0.64233000

C	5.24463800	1.05211600	-0.97222400
C	4.61941700	2.13215400	-0.26252400
C	4.07980300	3.04479500	0.31540100
C	3.40947100	3.99463100	2.58015700
C	2.89120700	7.31113300	-0.75453600
C	3.42313800	4.09166300	1.04385500
C	2.22786900	5.99807800	-0.30833600
C	0.85542300	5.77650000	-0.65880700
C	-0.30014100	5.57441900	-0.94687700
C	-4.24151800	4.70635800	-1.96211100
C	-3.60496100	5.74082400	-2.62934500
C	-2.29503600	6.04955300	-2.30036400
C	-1.66311500	5.30128800	-1.30012900
N	-2.27016300	4.30636000	-0.64263100
C	-3.53316100	4.01473100	-0.97300200
C	-4.15620400	2.93361900	-0.26303000
C	-4.67712300	2.01026400	0.31502700
C	-5.25596100	0.91852000	1.04360800
C	-6.30953400	-1.06989300	-0.30832000
C	-5.43137700	-2.14781600	-0.65828100
C	-4.67845800	-3.04757100	-0.94586100
C	2.96103800	-4.99599300	0.37430000
C	2.84653200	5.06196000	0.37480000
C	-5.80816300	-0.06596800	0.37469400
H	-3.39442000	-6.72302500	-3.39840200
H	-5.05176300	-4.94493100	-2.79734000
H	-1.20256700	-6.77191100	-2.18609100
H	6.46703000	2.34474600	-2.18628600
H	7.52045700	0.42212100	-3.39880400
H	6.80888100	-1.90220400	-2.79815400
H	-5.26321300	4.42638400	-2.18812400
H	-4.12431500	6.29936900	-3.40105400
H	-1.75590900	6.84566500	-2.79949600
C	-5.16533100	0.95550300	2.57991000
C	-7.77827600	-1.15194300	-0.75467700

C	1.75578700	-4.94852900	2.57987900
C	4.88635300	-6.15990300	-0.75475000
C	-7.81890800	-1.25063600	-2.28661100
H	-7.25714100	-2.11837200	-2.64104200
H	-8.85264500	-1.34892700	-2.63178000
H	-7.38790500	-0.35756300	-2.74748200
C	-8.41082300	-2.40788200	-0.13821800
H	-7.86596500	-3.30862300	-0.43169300
H	-8.40744100	-2.35182500	0.95399900
H	-9.44795400	-2.51218500	-0.47128200
C	-8.54852800	0.08892700	-0.30150000
H	-8.54809400	0.18380300	0.78774700
H	-8.11463200	1.00149600	-0.71859100
H	-9.58817900	0.02288100	-0.63438400
C	-3.68201300	1.00514000	2.97550100
H	-3.18334200	1.87316400	2.53807200
H	-3.58694100	1.06768400	4.06389500
H	-3.15789700	0.10730600	2.63726600
C	-5.88103500	2.21589500	3.08600800
H	-5.79930000	2.28347100	4.17502500
H	-5.44172200	3.11901900	2.65548100
H	-6.94332600	2.19582500	2.82522000
C	-5.81653900	-0.28770700	3.18714600
H	-6.87834100	-0.34927200	2.93195300
H	-5.33089500	-1.20272400	2.83857400
H	-5.73365700	-0.25263800	4.27701100
C	2.82691100	7.39532000	-2.28653200
H	3.25869600	8.33970800	-2.63166000
H	3.38524000	6.57552200	-2.74690200
H	1.79475500	7.34243000	-2.64153600
C	4.35068900	7.35808500	-0.30054100
H	4.43198800	7.31059800	0.78876400
H	4.92443100	6.52600900	-0.71705400
H	4.81337000	8.29144800	-0.63342000
C	2.11929700	8.48694900	-0.13879600

H	1.06697600	8.46525400	-0.43286000
H	2.16552900	8.45627500	0.95345400
H	2.54761000	9.43724700	-0.47185200
C	2.65687800	5.17936000	3.18714400
H	3.13338700	6.13025400	2.93205800
H	1.62168600	5.21503800	2.83826500
H	2.64560900	5.09006400	4.27700900
C	4.85872500	3.98587300	3.08665200
H	4.87623600	3.88110600	4.17565300
H	5.42226200	3.15457800	2.65610800
H	5.37146800	4.91651900	2.82615100
C	2.71210900	2.68447600	2.97556100
H	1.67262600	2.67833500	2.63695600
H	3.21558000	1.81911200	2.53837000
H	2.71845200	2.57096600	4.06396500
C	3.15827800	-4.89017800	3.18650900
H	3.74259800	-5.77899600	2.93172700
H	3.70755500	-4.01221300	2.83710100
H	3.08687600	-4.83516500	4.27636500
C	1.02253100	-6.19829600	3.08712800
H	0.92348600	-6.16050800	4.17614900
H	0.02062600	-6.26992800	2.65696800
H	1.57119000	-7.10828600	2.82681400
C	0.97111500	-3.68855300	2.97485900
H	1.48632600	-2.78591900	2.63571300
H	-0.03015000	-3.69116900	2.53788800
H	0.86990100	-3.63666500	4.06325800
C	4.99181500	-6.14637200	-2.28672200
H	5.46252700	-5.22623300	-2.64160200
H	5.59354800	-6.99272800	-2.63170600
H	4.00276300	-6.21961600	-2.74732500
C	6.29044400	-6.07970300	-0.13863400
H	6.89913200	-6.92594800	-0.47152600
H	6.79816100	-5.15766100	-0.43256100
H	6.24045200	-6.10437400	0.95360200

C 4.19673000 -7.44709700 -0.30091500

H 4.11454300 -7.49367000 0.78836500

H 3.18938000 -7.52760400 -0.71778600

H 4.77353200 -8.31469200 -0.63351900

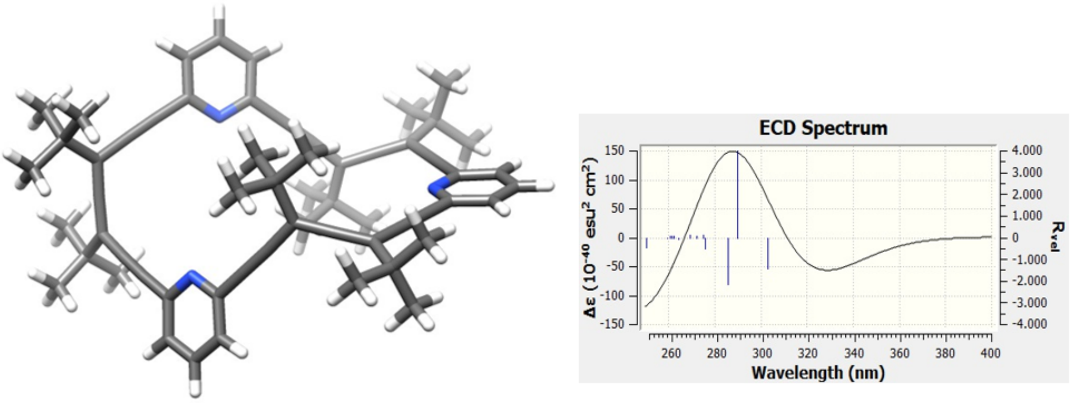

(P_3)-**3** Conformer **B** (C_2, 0.1)

Figure S50. Optimized structure of (P_3)-**3** conformer B and ECD calculated spectrum. In parentheses, the point group symmetry and the relative energy in kcal/mol for this conformer are indicated.

C -2.35483000 4.58438200 3.93343500

C -1.32944900 4.78526300 3.02302000

C -1.39005200 4.11949700 1.79354400

N -2.39217000 3.29941000 1.45793600

C -3.36964800 3.10339500 2.35028400

C -3.39461400 3.73065800 3.60131600

C -4.40278100 2.18039500 1.97883500

C -5.23158400 1.36398800 1.65410400

C -6.17227500 0.35513200 1.26389000

C -6.17245700 -0.35462900 -1.26384200

C -5.23195400 -1.36363200 -1.65413000

C -4.40325400 -2.18016600 -1.97880500

C -1.33023700 -4.78541000 -3.02297500

C -2.35567700 -4.58453100 -3.93332500

C -3.39535300 -3.73067800 -3.60120900

C -3.37022600 -3.10328800 -2.35024400

N -2.39268700 -3.29929400 -1.45795900

C -1.39067100 -4.11950600 -1.79356600

C	-0.34626200	-4.28640800	-0.82414700
C	0.54060400	-4.39720500	-0.01220800
C	1.06676200	-4.48648800	2.47251800
C	4.83184200	-5.94042200	0.04645000
C	1.54251000	-4.50182200	1.00741100
C	4.07400300	-4.63034200	0.32314600
C	4.79937700	-3.39804000	0.21814600
C	5.41874700	-2.36371600	0.14025500
C	7.57140100	1.19175100	-0.06435300
C	8.27396700	-0.00046100	-0.00006400
C	7.57122300	-1.19257100	0.06419900
C	6.17174600	-1.14497600	0.06376200
N	5.48180800	-0.00025600	-0.00011300
C	6.17191600	1.14436100	-0.06396800
C	5.41909800	2.36321000	-0.14051400
C	4.79980000	3.39758500	-0.21829900
C	4.07454500	4.62995900	-0.32327600
C	1.54303800	4.50171200	-1.00754500
C	0.54110100	4.39716400	0.01205000
C	-0.34571700	4.28640600	0.82404800
C	-6.20370000	0.00027100	0.00002100
C	2.80557600	-4.58348200	0.66100700
C	2.80610900	4.58321800	-0.66111800
H	-2.34142200	5.08562000	4.89563000
H	-0.49266500	5.43720600	3.24235100
H	-4.21273500	3.53813900	4.28484900
H	-0.49353300	-5.43745500	-3.24230800
H	-2.34239800	-5.08587400	-4.89546700
H	-4.21351700	-3.53815900	-4.28469100
H	8.07771900	2.14800500	-0.11683400
H	9.35898300	-0.00054100	-0.00004500
H	8.07739900	-2.14889900	0.11669600
C	4.83244100	5.93994800	-0.04630700
C	1.06729000	4.48678600	-2.47265600
C	-7.05856000	-0.27476000	2.35091100

C	-7.05876600	0.27536200	-2.35078500
C	0.31181600	3.17302500	-2.72260700
H	-0.53609700	3.06621600	-2.04222800
H	-0.06746400	3.14954200	-3.74882400
H	0.96974500	2.31128100	-2.58177100
C	0.12568100	5.67847400	-2.69714600
H	-0.72940600	5.63909600	-2.01818000
H	0.64659700	6.62688700	-2.53546000
H	-0.25391900	5.66950900	-3.72334600
C	2.25830900	4.58305000	-3.42694100
H	2.81374400	5.51426600	-3.28318200
H	2.95157200	3.75004400	-3.28482900
H	1.90457000	4.55833900	-4.46141800
C	5.38639600	5.89028800	1.38510200
H	6.04164100	5.02711800	1.52540500
H	5.96290700	6.79550100	1.59886200
H	4.57558800	5.82118800	2.11554100
C	5.99143600	6.06100900	-1.04602300
H	6.57101500	6.96736200	-0.84592800
H	6.66485100	5.20326400	-0.97513800
H	5.61878400	6.11432200	-2.07288600
C	3.89987500	7.14314500	-0.19193300
H	3.49667400	7.21658900	-1.20554000
H	3.05690500	7.08003800	0.50113500
H	4.44798300	8.06521000	0.02121700
C	5.38583900	-5.89106100	-1.38495300
H	6.04113200	-5.02795200	-1.52540200
H	5.96230900	-6.79634500	-1.59852400
H	4.57505500	-5.82206000	-2.11542700
C	3.89921800	-7.14355100	0.19228100
H	3.49598700	-7.21678200	1.20589200
H	3.05626900	-7.08053900	-0.50082000
H	4.44729100	-8.06568100	-0.02067600
C	5.99080500	-6.06134000	1.04622000
H	6.66425500	-5.20363200	0.97519200

H	5.61812300	-6.11444600	2.07308200
H	6.57035500	-6.96775200	0.84630900
C	2.25776000	-4.58270500	3.42683200
H	2.81305800	-5.51403700	3.28329400
H	2.95114900	-3.74983600	3.28452600
H	1.90402100	-4.55769800	4.46130100
C	0.12497600	-5.67798700	2.69727500
H	-0.25463100	-5.66872800	3.72346900
H	-0.73009800	-5.63864200	2.01829300
H	0.64575600	-6.62651300	2.53581400
C	0.31147600	-3.17255900	2.72215900
H	0.96953300	-2.31094400	2.58113300
H	-0.53641300	-3.06578300	2.04174400
H	-0.06781300	-3.14878400	3.74836600
C	-7.97151300	-1.34371700	1.74895000
H	-8.64051200	-0.91994100	0.99479800
H	-7.39264700	-2.14160400	1.27672500
H	-8.58816700	-1.78971200	2.53433000
C	-7.90826400	0.83124000	2.99278700
H	-8.52068000	0.41767200	3.79979500
H	-7.27769000	1.61886100	3.41295500
H	-8.57668300	1.28870200	2.25774800
C	-6.15059400	-0.91355900	3.41196800
H	-5.54440900	-1.71270800	2.97705900
H	-5.47328800	-0.17555200	3.84844700
H	-6.75539200	-1.34127900	4.21743900
C	-6.15081800	0.91398000	-3.41196600
H	-5.47367400	0.17585100	-3.84849200
H	-6.75563700	1.34176400	-4.21738600
H	-5.54446000	1.71305000	-2.97715200
C	-7.90871200	-0.83053100	-2.99252700
H	-8.52115200	-0.41689900	-3.79948400
H	-7.27830600	-1.61826600	-3.41273400
H	-8.57712100	-1.28786300	-2.25739800
C	-7.97148700	1.34448100	-1.74876300

H	-8.64046900	0.92083700	-0.99452200
H	-7.39244600	2.14229600	-1.27663100
H	-8.58815800	1.79054300	-2.53409300

(P_4)-**4**

Through DFT computational calculations, four conformers were identified for the allenophane (P_4)-**4**. Among these, one exhibited a crown-shaped conformation denoted as **A**, characterized by C_4 symmetry (**Figure S51**). Additionally, conformers—**B**, **C** and **D**—adopted different helical conformations, with D_2, C_2 and C_1 symmetry, respectively (**Figure S52-S54).**

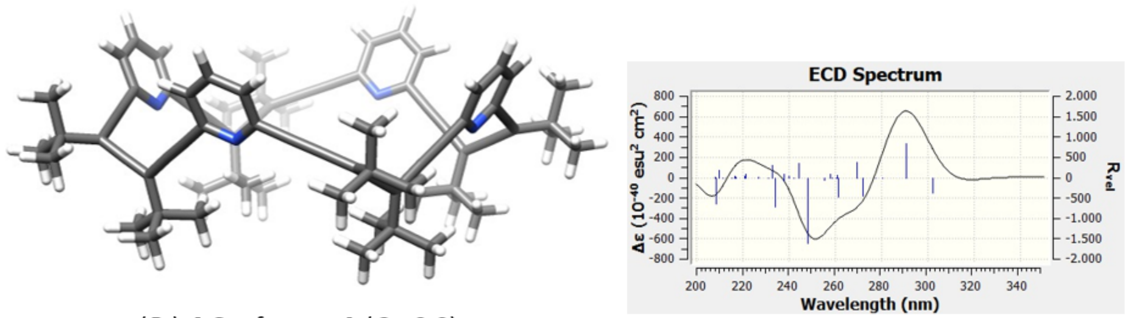

(P_4)-**4** Conformer **A** (C_4, 0.0)

Figure S51. Optimized structure of (P_4)-**4** conformer A and ECD calculated spectrum. In parentheses, the point group symmetry and the relative energy in kcal/mol for this conformer are indicated.

C	6.14283300	4.00526000	-1.02996300
C	2.07929500	6.35927000	-0.46676300
C	6.92082500	3.18117100	-0.61246700
C	1.09194900	6.58853800	0.18996800
C	6.59047700	-1.09204700	0.18974700
C	6.36101400	-2.07947700	-0.46678800
C	-3.18133800	6.92006700	-0.61160800
C	-4.00570800	6.14244400	-1.02923300
C	4.96861400	-5.21018800	-1.54325800
C	6.10523300	-3.23714300	-1.27335000
C	6.77316700	-3.39937500	-2.49316400
C	6.50778100	-4.53115200	-3.24713700
C	5.59238100	-5.45690600	-2.77182300
C	3.18133700	-6.91998300	-0.61161300
C	-1.09198300	-6.58871600	0.19002300
C	-2.07934700	-6.35945800	-0.46668200

C	-6.14283100	-4.00533100	-1.02982100
C	-6.92080900	-3.18121400	-0.61235600
C	-6.59038600	1.09205600	0.18964100
C	-6.10522600	3.23722300	-1.27337900
N	-5.22009900	4.12417100	-0.80347100
C	-6.77318400	3.39948500	-2.49317500
C	-4.96865100	5.21030000	-1.54323500
H	7.00806900	-4.68945800	-4.19731600
H	7.47904800	-2.64682200	-2.82437700
H	-7.00814700	4.68962900	-4.19727300
C	-6.50783800	4.53129700	-3.24711100
C	-7.84264300	-2.20745100	-0.10233300
C	-6.83724900	-0.05402500	1.01378800
C	-2.20733000	7.84148700	-0.10130800
C	-0.05400400	6.83531600	1.01431200
C	7.84265800	2.20745200	-0.10235900
C	6.83734400	0.05404600	1.01387600
C	2.20736600	-7.84145300	-0.10133000
C	0.05400500	-6.83541800	1.01434400
C	-7.35571500	-1.12838700	0.46534500
C	-1.12835900	7.35409700	0.46614800
C	7.35572900	1.12843400	0.46540600
C	1.12838400	-7.35412300	0.46615600
N	5.22009500	-4.12409000	-0.80346000
C	-5.59244700	5.45705100	-2.77178000
H	-5.34971600	6.35460000	-3.32853700
H	5.34962300	-6.35442900	-3.32861000
H	-7.47905100	2.64692700	-2.82440500
C	4.00566700	-6.14233400	-1.02926700
C	-6.36095600	2.07951200	-0.46686600
C	-2.48700400	9.34688600	-0.24654100
C	0.04319300	6.46437100	2.50495400
C	-6.46672500	0.04304100	2.50453900
C	9.34793500	2.48742000	-0.24828700
C	6.46673800	-0.04297100	2.50461200

C	-0.04316800	-6.46443400	2.50497700
C	2.48707000	-9.34683800	-0.24664900
C	-9.34792100	-2.48740000	-0.24830200
C	3.79173600	-9.67935500	0.49178400
H	4.03389500	-10.74094500	0.37340800
H	4.62820000	-9.09270100	0.10144000
H	3.70076200	-9.46758900	1.56185700
C	2.63932000	-9.67453400	-1.73931300
H	3.44316300	-9.08769500	-2.19287600
H	2.87578800	-10.73575200	-1.87112300
H	1.71401200	-9.46061600	-2.28351600
C	1.33852200	-10.16765300	0.34171700
H	1.20901000	-9.96674800	1.40958900
H	0.39130800	-9.94666300	-0.15931400
H	1.54617200	-11.23573600	0.22199000
C	-1.18531800	-7.27040900	3.14032300
H	-1.29576900	-6.99870500	4.19547800
H	-2.13623500	-7.07381100	2.63731900
H	-0.98562200	-8.34564100	3.08503800
C	-0.34605300	-4.96238900	2.61269200
H	-1.27430300	-4.70615200	2.09468100
H	-0.45170800	-4.67643000	3.66463700
H	0.46306100	-4.36799000	2.17718700
C	1.27080700	-6.77497200	3.22349400
H	1.51190900	-7.84177600	3.17649100
H	2.10632100	-6.21958800	2.78729700
H	1.18991700	-6.49450300	4.27840600
C	-9.68053100	-3.79211800	0.49000000
H	-10.74201800	-4.03448600	0.37113300
H	-9.09353100	-4.62847400	0.09994600
H	-9.46928300	-3.70108300	1.56017000
C	-10.16917900	-1.33898600	0.33970300
H	-9.96877900	-1.20945100	1.40766800
H	-9.94811000	-0.39173500	-0.16122200
H	-11.23717300	-1.54680700	0.21948800

C	-9.67494700	-2.63972000	-1.74110400
H	-9.46100800	-1.71436300	-2.28521700
H	-9.08773100	-3.44342900	-2.19441600
H	-10.73605500	-2.87642900	-1.87336800
C	-7.27296400	1.18507400	3.13975000
H	-7.00156600	1.29544900	4.19499200
H	-7.07629900	2.13605000	2.63688500
H	-8.34816500	0.98529700	3.08414100
C	-4.96472900	0.34601400	2.61272900
H	-4.70840100	1.27436200	2.09493900
H	-4.67907500	0.45152300	3.66477200
H	-4.37015400	-0.46299400	2.17726600
C	-6.77739400	-1.27102400	3.22284000
H	-7.84417000	-1.51218300	3.17550400
H	-6.22183400	-2.10646400	2.78672300
H	-6.49723700	-1.19022600	4.27784200
C	1.18540300	7.27030700	3.14024000
H	1.29586600	6.99863600	4.19540300
H	2.13629600	7.07363800	2.63721900
H	0.98576400	8.34554800	3.08492100
C	0.34600300	4.96231400	2.61270900
H	0.45166500	4.67638300	3.66466100
H	-0.46315100	4.36794300	2.17723900
H	1.27422900	4.70601200	2.09468600
C	-1.27074600	6.77499900	3.22349900
H	-1.51180900	7.84180900	3.17644800
H	-2.10629600	6.21962300	2.78736000
H	-1.18983200	6.49458300	4.27842300
C	9.68048100	3.79221100	0.48991400
H	10.74197100	4.03458400	0.37108900
H	9.09349200	4.62852200	0.09974500
H	9.46916900	3.70127500	1.56008000
C	10.16918800	1.33907600	0.33986100
H	9.96873100	1.20962800	1.40782500
H	9.94817000	0.39177700	-0.16099500

H	11.23718400	1.54691200	0.21968800
C	9.67503100	2.63961300	-1.74108800
H	9.08781300	3.44326500	-2.19450000
H	10.73613800	2.87634000	-1.87332300
H	9.46114300	1.71420100	-2.28512600
C	6.77727000	1.27115100	3.22286900
H	7.84403500	1.51237400	3.17560200
H	6.22169100	2.10653400	2.78666800
H	6.49703900	1.19038800	4.27785400
C	7.27302100	-1.18491400	3.13992800
H	7.00155900	-1.29526200	4.19515500
H	7.07646300	-2.13592800	2.63709300
H	8.34821000	-0.98505700	3.08438300
C	4.96475800	-0.34604700	2.61273000
H	4.37014900	0.46290900	2.17721700
H	4.70852200	-1.27442500	2.09494700
H	4.67905700	-0.45155200	3.66476100
C	-2.63929500	9.67466600	-1.73918100
H	-3.44316300	9.08786500	-2.19275100
H	-2.87575000	10.73589400	-1.87092500
H	-1.71400800	9.46076300	-2.28342600
C	-1.33841700	10.16764300	0.34182900
H	-1.20888400	9.96669300	1.40969000
H	-0.39122200	9.94664500	-0.15923500
H	-1.54603800	11.23573700	0.22214900
C	-3.79163700	9.67939400	0.49195400
H	-4.03377800	10.74099500	0.37363600
H	-4.62812700	9.09277600	0.10161200
H	-3.70063000	9.46757700	1.56201400
C	-5.21028400	-4.96796600	-1.54367200
C	-5.45659800	-5.59178300	-2.77229300
C	-4.53048100	-6.50688700	-3.24746800
C	-3.39876100	-6.77195200	-2.49329800
C	-3.23694900	-6.10399900	-1.27343700
N	-4.12423900	-5.21912900	-0.80368900

H	-4.68846400	-7.00719600	-4.19768800
H	-6.35409900	-5.34928700	-3.32922900
H	-2.64593100	-7.47758900	-2.82439800
C	3.23688100	6.10383500	-1.27354700
C	3.39862700	6.77174900	-2.49343700
C	4.53032500	6.50668600	-3.24764200
C	5.45648500	5.59162300	-2.77247100
C	5.21023700	4.96784500	-1.54381800
N	4.12421600	5.21900800	-0.80380000
H	4.68825700	7.00696500	-4.19788700
H	2.64576300	7.47735100	-2.82453600
H	6.35397100	5.34913300	-3.32943400

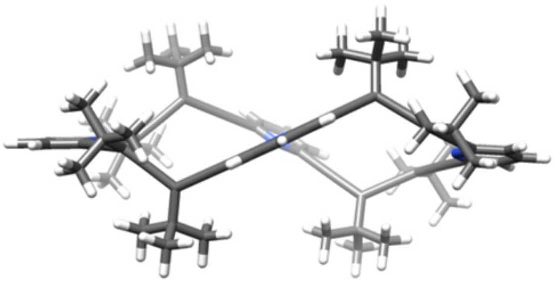

(P_4)-**4** Conformer **B** (D_2, 3.0)

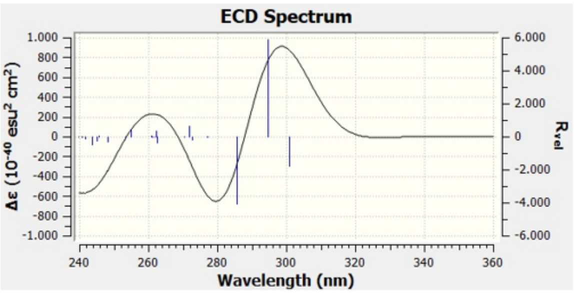

Figure S52. Optimized structure of (P_4)-**4** conformer B and ECD calculated spectrum. In parentheses, the point group symmetry and the relative energy in kcal/mol for this conformer are indicated.

C	1.69008200	-1.65204600	-3.87734800
C	-1.69018300	1.65331800	-3.87679700
C	2.42212100	-2.37875300	-3.24943600
C	-2.42219900	2.37985900	-3.24866700
C	6.65323900	-2.52156800	-2.20386100
C	7.34030500	-1.76570500	-1.55903600
C	-6.65324700	2.52231200	-2.20288200
C	-7.34028100	1.76628600	-1.55821400
C	8.11292100	0.85714400	0.76261600
C	8.11291500	-0.85698200	-0.76299700
C	9.51199300	-0.89155300	-0.79644700
C	10.21376800	0.00022100	-0.00035600

C	9.51199900	0.89190100	0.79584700
C	6.65327200	2.52155900	2.20370700
C	2.42221800	2.37875300	3.24946200
C	1.69023100	1.65196900	3.87734200
C	-1.68998000	-1.65346000	3.87670700
C	-2.42206200	-2.37993200	3.24857300
C	-6.65321400	-2.52223200	2.20307800
C	-8.11288700	-0.85723300	0.76268800
N	-7.42489900	0.00006200	0.00013800
C	-9.51196400	-0.89188000	0.79604900
C	-8.11288800	0.85737500	-0.76238500
H	11.29924800	0.00029400	-0.00044100
H	10.01974000	-1.60602000	-1.43390600
H	-11.29921700	0.00012600	0.00021800
C	-10.21373700	0.00010800	0.00019500
C	-3.23649800	-3.25308300	2.45435500
C	-5.81170900	-3.42275400	2.93540800
C	-5.81179800	3.42279000	-2.93532700
C	-3.23655500	3.25309800	-2.45446400
C	3.23648300	-3.25218600	-2.45545900
C	5.81170600	-3.42185000	-2.93645800
C	5.81179400	3.42178600	2.93642900
C	3.23654400	3.25226600	2.45552700
C	-4.51719900	-3.35656000	2.72013800
C	-4.51727100	3.35659300	-2.72016700
C	4.51719400	-3.35562200	-2.72121400
C	4.51727200	3.35564700	2.72122500
N	7.42492900	0.00003200	-0.00013200
C	-9.51196700	0.89207100	-0.79568800
H	-10.01971900	1.60677800	-1.43287500
H	10.01975200	1.60643800	1.43322400
H	-10.01971500	-1.60657000	1.43325500
C	7.34031600	1.76576400	1.55878000
C	-7.34027800	-1.76617500	1.55847800
C	-6.47050700	4.41558300	-3.90536900
C	-2.55303500	4.00705800	-1.29798600
C	-6.47034600	-4.41560800	3.90543500
C	2.55299600	-4.00638500	-1.29911900

C	6.47031100	-4.41446500	-3.90674700
C	2.55299000	4.00664300	1.29934800
C	6.47047600	4.41428600	3.90679200
C	-2.55307200	-4.00700100	1.29779500
C	7.43487000	5.31083500	3.11670800
H	7.94767900	6.00407100	3.79204400
H	8.19268200	4.71596300	2.59874400
H	6.89693700	5.89986100	2.36707500
C	7.25182700	3.61938800	4.96339400
H	8.00312300	2.97507900	4.49773200
H	7.76501700	4.30285800	5.64833700
H	6.58087300	2.98569900	5.55176300
C	5.41183700	5.27728400	4.59492900
H	4.84300600	5.86720300	3.86971800
H	4.70291000	4.66420300	5.15944900
H	5.89284100	5.97092400	5.29192200
C	1.41470700	4.86471500	1.86925100
H	0.89507200	5.38549000	1.05753400
H	0.68406500	4.24987500	2.40246200
H	1.79871600	5.61835600	2.56488400
C	1.98413000	2.96939800	0.31929900
H	1.27208800	2.30248300	0.81303600
H	1.46412700	3.47407500	-0.50198600
H	2.78286300	2.35612100	-0.10853400
C	3.55718400	4.90143600	0.57113900
H	3.97838800	5.65983100	1.23911200
H	4.38401500	4.31824600	0.15552500
H	3.05838000	5.41874600	-0.25446400
C	-1.41470900	-4.86528600	1.86721800
H	-0.89518200	-5.38574400	1.05522900
H	-0.68399800	-4.25065100	2.40057000
H	-1.79862100	-5.61919900	2.56260900
C	-3.55733900	-4.90152200	0.56935100
H	-3.97846100	-5.66017800	1.23708000
H	-4.38422200	-4.31818000	0.15405100
H	-3.05862100	-5.41850800	-0.25650600
C	-1.98434200	-2.96935700	0.31809400
H	-2.78313500	-2.35593100	-0.10941200

H	-1.27226400	-2.30262000	0.81202000
H	-1.46441100	-3.47369500	-0.50344500
C	-7.43480800	-5.31184000	3.11507700
H	-7.94757500	-6.00532900	3.79018500
H	-8.19265100	-4.71676100	2.59739800
H	-6.89693800	-5.90058500	2.36517800
C	-7.25161700	-3.62109100	4.96238500
H	-8.00294100	-2.97660500	4.49701300
H	-7.76476300	-4.30480900	5.64711300
H	-6.58061500	-2.98762300	5.55093900
C	-5.41168300	-5.27888400	4.59318400
H	-4.84290700	-5.86854100	3.86771800
H	-4.70271100	-4.66602900	5.15789300
H	-5.89265900	-5.97277800	5.28994500
C	-1.41470600	4.86530300	-1.86753400
H	-0.89511000	5.38579400	-1.05561000
H	-0.68404300	4.25063200	-2.40091100
H	-1.79866200	5.61918700	-2.56293300
C	-1.98424800	2.96945600	-0.31827500
H	-1.46426300	3.47383100	0.50320600
H	-2.78301700	2.35605500	0.10931300
H	-1.27220500	2.30269200	-0.81221500
C	-3.55724200	4.90162300	-0.56951100
H	-3.97840800	5.66025100	-1.23724400
H	-4.38409900	4.31830700	-0.15412300
H	-3.05845900	5.41864500	0.25628500
C	1.41466400	-4.86452800	-1.86881800
H	0.89508500	-5.38517900	-1.05698600
H	0.68398900	-4.24976000	-2.40206600
H	1.79861700	-5.61827500	-2.56436600
C	3.55722500	-4.90108500	-0.57084600
H	3.97837300	-5.65958400	-1.23873700
H	4.38409300	-4.31784700	-0.15537000
H	3.05846900	-5.41826800	0.25486600
C	1.98421400	-2.96898700	-0.31918700
H	1.27215400	-2.30213100	-0.81297900
H	1.46425000	-3.47353200	0.50220400
H	2.78298300	-2.35566100	0.10850800

C	5.41161100	-5.27748100	-4.59476500
H	4.84278100	-5.86730700	-3.86947800
H	4.70269000	-4.66442100	-5.15931800
H	5.89256000	-5.97120800	-5.29171100
C	7.43469900	-5.31098100	-3.11661700
H	7.94745000	-6.00430200	-3.79190900
H	8.19255700	-4.71609200	-2.59873900
H	6.89677100	-5.89991300	-2.36690600
C	7.25165800	-3.61969500	-4.96345000
H	6.58070700	-2.98603400	-5.55185500
H	8.00299400	-2.97537300	-4.49787400
H	7.76479600	-4.30324900	-5.64834800
C	-7.25188000	3.62100200	-4.96219500
H	-8.00317000	2.97655700	-4.49671300
H	-7.76507900	4.30467900	-5.64692600
H	-6.58093800	2.98748600	-5.55076500
C	-5.41189400	5.27880100	-4.59326900
H	-4.84305200	5.86850600	-3.86789300
H	-4.70297300	4.66589900	-5.15799300
H	-5.89292200	5.97264800	-5.29004100
C	-7.43488900	5.31188100	-3.11498800
H	-7.94771000	6.00532700	-3.79009900
H	-8.19269300	4.71684400	-2.59719900
H	-6.89694700	5.90067300	-2.36517800
C	0.82499300	-0.79557100	-4.63800100
C	0.86195800	-0.82597900	-6.03686700
C	-0.00017800	0.00098000	-6.73947100
C	-0.86225100	0.82777200	-6.03659000
C	-0.82515300	0.79703200	-4.63773700
N	-0.00004800	0.00065000	-3.94845800
H	-0.00022800	0.00111000	-7.82492900
H	1.55291700	-1.48988600	-6.54301800
H	-1.55325400	1.49180000	-6.54252000
C	0.82523500	0.79537100	4.63796700
C	0.86237100	0.82557000	6.03683400
C	0.00033700	-0.00151100	6.73941900
C	-0.86180200	-0.82821600	6.03651900
C	-0.82487900	-0.79726200	4.63766400

N	0.00012400	-0.00075800	3.94840500
H	0.00041800	-0.00180200	7.82487800
H	1.55337600	1.48941800	6.54299900
H	-1.55272800	-1.49233800	6.54243200

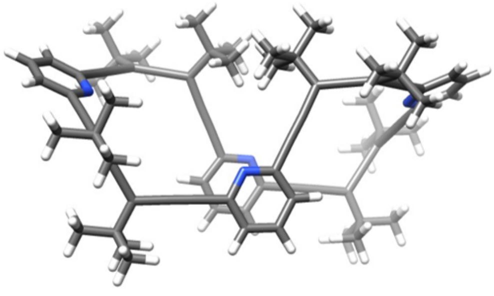

(P_4)-**4** Conformer **C** (C_2, 1.8)

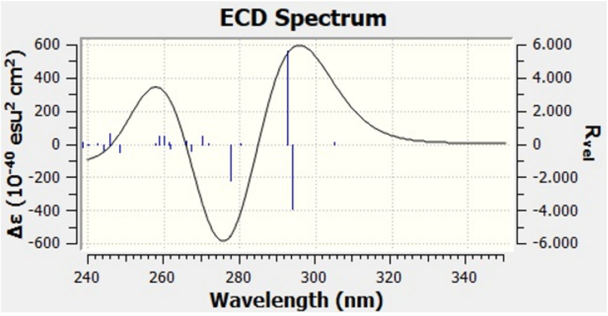

Figure S53. Optimized structure of (P_4)-**4** conformer C and ECD calculated spectrum. In parentheses, the point group symmetry and the relative energy in kcal/mol for this conformer are indicated.

C	5.66132300	-3.95587800	2.08306400
C	7.85762700	-0.08519900	0.46751500
C	4.64263900	-4.59489400	2.19787100
C	7.81210300	0.95512600	-0.14433800
C	0.54606700	-4.09850600	0.72312100
C	0.00388700	-4.04087600	-0.35436100
C	4.99647000	2.75432500	-2.99224400
C	3.84121600	3.10384100	-2.94509800
C	-2.46696800	-3.51332400	-2.90556400
C	-0.60359400	-3.97007900	-1.65093600
C	0.14900200	-4.27761100	-2.79083200
C	-0.46387900	-4.18988200	-4.03042600
C	-1.79238300	-3.80138400	-4.09823200
C	-4.99652200	-2.75445800	-2.99224900
C	-7.81203200	-0.95506500	-0.14443700
C	-7.85761200	0.08537600	0.46721300
C	-5.66138600	3.95606900	2.08283100
C	-4.64266900	4.59500200	2.19781900
C	-0.54602200	4.09873100	0.72316200
C	0.60375200	3.97066500	-1.65085700
N	1.88795200	3.59781100	-1.70242700

C	-0.14868300	4.27871800	-2.79071800
C	2.46708900	3.51376600	-2.90548800
H	0.08957200	-4.41952200	-4.93548500
H	1.18709900	-4.57063800	-2.68729900
H	-0.08907400	4.42120800	-4.93532700
C	0.46425600	4.19116700	-4.03029600
C	-3.43938400	5.36111200	2.34120200
C	-1.13784100	4.13620900	2.02713300
C	6.36616600	2.34017100	-3.06922000
C	7.77327600	2.19921900	-0.85697300
C	3.43938100	-5.36109300	2.34105500
C	1.13780000	-4.13622700	2.02713000
C	-6.36630600	-2.34058800	-3.06918000
C	-7.77315100	-2.19932800	-0.85678400
C	-2.28516800	4.75384800	2.18686900
C	7.07130400	2.27228600	-1.96357600
C	2.28514500	-4.75384800	2.18680200
C	-7.07132800	-2.27255500	-1.96347000
N	-1.88789000	-3.59756100	-1.70248300
C	1.79266000	3.80233500	-4.09812300
H	2.31264200	3.71739300	-5.04523900
H	-2.31232400	-3.71631900	-5.04535900
H	-1.18670700	4.57200300	-2.68717300
C	-3.84119200	-3.10373100	-2.94514600
C	-0.00378500	4.04127100	-0.35430100
C	6.93745400	1.99752300	-4.45675500
C	8.56729100	3.38477500	-0.27979300
C	-0.38299800	3.43872500	3.17341700
C	3.57197200	-6.85683500	2.67762100
C	0.38283400	-3.43904500	3.17351100
C	-8.56689900	-3.38485500	-0.27919100
C	-6.93781600	-1.99839900	-4.45673500
C	-3.57191600	6.85680400	2.67802100
C	-6.83433000	-3.24031600	-5.35373200
H	-7.19921300	-3.01143900	-6.36073300
H	-5.79917000	-3.58369400	-5.43601500
H	-7.43335100	-4.06456200	-4.95333200
C	-6.10774300	-0.85406900	-5.05704100

H	-5.05171500	-1.12814900	-5.13236900
H	-6.47072500	-0.61135200	-6.06155200
H	-6.18002400	0.04674900	-4.43990500
C	-8.40014700	-1.56360800	-4.34754600
H	-9.02611900	-2.36121900	-3.93549400
H	-8.50833800	-0.68419400	-3.70589200
H	-8.78641700	-1.31058400	-5.34003300
C	-10.05076300	-2.99754000	-0.20404900
H	-10.63235300	-3.81289000	0.23913000
H	-10.19557400	-2.10378100	0.40957400
H	-10.45448300	-2.79387700	-1.20115900
C	-8.03645400	-3.68448900	1.13072300
H	-8.12442300	-2.80990900	1.78139300
H	-8.60574100	-4.50460900	1.58126600
H	-6.98255700	-3.97702200	1.09767100
C	-8.40295300	-4.62466600	-1.15969600
H	-8.78479400	-4.45220600	-2.17085000
H	-7.35292900	-4.92040200	-1.24112400
H	-8.95984000	-5.46197800	-0.72726600
C	-4.30691800	6.99776800	4.01881500
H	-4.44741600	8.05585500	4.26435400
H	-5.29153700	6.52279600	3.98199500
H	-3.73668700	6.53246000	4.82915900
C	-2.19362300	7.51252300	2.77621000
H	-1.59011200	7.06068200	3.56935800
H	-1.63906900	7.41989900	1.83774600
H	-2.30511600	8.57779900	3.00214900
C	-4.38519700	7.53815200	1.56759100
H	-3.86961400	7.46555200	0.60489700
H	-5.37120100	7.07750500	1.45893500
H	-4.52719900	8.59906500	1.79976800
C	1.00342600	4.08355700	3.31451400
H	1.57298500	3.58034800	4.10303100
H	1.57199800	4.00850200	2.38357100
H	0.91882300	5.14287000	3.57881300
C	-0.22919800	1.95170300	2.81989400
H	0.31111100	1.82120000	1.87838600
H	0.33075100	1.43522600	3.60687400

H	-1.20641200	1.46856300	2.72191000
C	-1.15358900	3.57509000	4.48747800
H	-1.27109900	4.62415800	4.77739000
H	-2.15054200	3.13027800	4.41471100
H	-0.61063100	3.06341400	5.28837800
C	10.05111400	2.99727100	-0.20481400
H	10.63289500	3.81264100	0.23807700
H	10.19590300	2.10363300	0.40899100
H	10.45463500	2.79332000	-1.20194500
C	8.03712600	3.68481300	1.13013800
H	8.60660200	4.50496000	1.58039400
H	6.98326400	3.97748500	1.09719600
H	8.12508300	2.81037400	1.78099900
C	8.40336700	4.62440100	-1.16056100
H	8.78500600	4.45165100	-2.17174100
H	7.35337000	4.92026700	-1.24187500
H	8.96044700	5.46173600	-0.72842400
C	4.30695900	-6.99799800	4.01840200
H	4.44749400	-8.05612200	4.26376100
H	5.29156100	-6.52298200	3.98167800
H	3.73669700	-6.53285100	4.82881700
C	2.19370400	-7.51262700	2.77567800
H	1.59016300	-7.06094200	3.56889200
H	1.63916200	-7.41987000	1.83722100
H	2.30523800	-8.57793500	3.00144100
C	4.38529900	-7.53795900	1.56708600
H	5.37128500	-7.07725000	1.45852400
H	4.52734400	-8.59890500	1.79908600
H	3.86972700	-7.46521700	0.60439700
C	1.15334200	-3.57565200	4.48759700
H	1.27090400	-4.62477700	4.77728200
H	2.15026900	-3.13075600	4.41499900
H	0.61029200	-3.06419300	5.28857300
C	-1.00355900	-4.08400000	3.31436800
H	-1.57321200	-3.58099600	4.10294700
H	-1.57206400	-4.00878700	2.38339700
H	-0.91890400	-5.14336300	3.57844900
C	0.22896000	-1.95195500	2.82031100

H	1.20614900	-1.46872800	2.72250200
H	-0.31129000	-1.82127400	1.87879500
H	-0.33107700	-1.43569400	3.60737000
C	6.10708200	0.85321300	-5.05668800
H	5.05110300	1.12748900	-5.13197000
H	6.46990800	0.61017500	-6.06117800
H	6.17924900	-0.04746600	-4.43933700
C	8.39970800	1.56245900	-4.34761700
H	9.02588900	2.36004200	-3.93582900
H	8.50778700	0.68318100	-3.70575800
H	8.78581900	1.30911100	-5.34008300
C	6.83412600	3.23923900	-5.35404600
H	7.19885700	3.01004200	-6.36103000
H	5.79902700	3.58280700	-5.43630800
H	7.43335400	4.06346300	-4.95391300
C	-6.87493000	3.19985700	1.96856100
C	-8.05210800	3.68068300	2.55513700
C	-9.20691400	2.92399600	2.43865300
C	-9.15714200	1.72358000	1.74798100
C	-7.93693900	1.32275500	1.19055400
N	-6.81551400	2.04430400	1.29654700
H	-10.13662200	3.26603900	2.88236200
H	-8.04197700	4.62441800	3.08792600
H	-10.03445200	1.09823900	1.63042900
C	7.93686300	-1.32246200	1.19106600
C	9.15694800	-1.72308200	1.74890100
C	9.20664400	-2.92339100	2.43976200
C	8.05188000	-3.68017800	2.55602500
C	6.87482100	-3.19955800	1.96904500
N	6.81548000	-2.04410500	1.29684800
H	10.13625800	-3.26527500	2.88379000
H	10.03422700	-1.09766600	1.63151200
H	8.04169300	-4.62383900	3.08894400

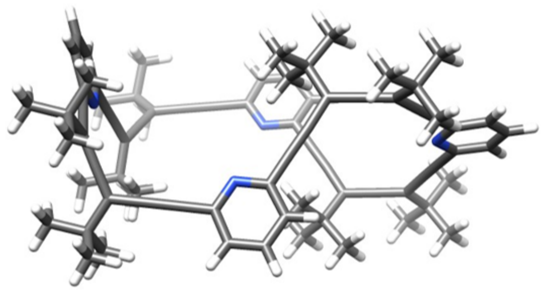

(P_4)-**4** Conformer **D** (C_1, 0.7)

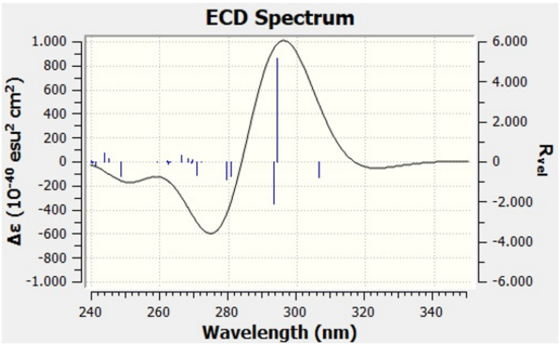

Figure S54. Optimized structure of (P_4)-**4** conformer D and ECD calculated spectrum. In parentheses, the point group symmetry and the relative energy in kcal/mol for this conformer are indicated.

C	-6.91225700	-3.31501600	-1.34495600
C	-7.81960700	0.92518300	0.55006600
C	-6.10411300	-4.16235900	-1.64206300
C	-7.40778200	1.92327500	1.09186300
C	-1.87967000	-4.80968300	-0.67905400
C	-1.22460300	-4.98816700	0.31970100
C	-3.67586300	4.23185600	0.90486700
C	-3.07002800	4.87369200	0.08045600
C	1.56302400	-5.12824500	2.57759000
C	-0.47234400	-5.18964900	1.52337800
C	-1.12186800	-5.59778000	2.69458100
C	-0.36585500	-5.77099400	3.84294600
C	0.99863500	-5.53355800	3.79311100
C	4.15427500	-4.63360600	2.42666100
C	6.67773400	-2.93236100	-0.68883100
C	6.69181000	-1.86574700	-1.25554000
C	5.74704500	2.76936700	-1.18214000
C	5.33253000	3.73869500	-0.59302700
C	2.23298800	6.69663700	-1.34426600
C	-0.38174700	6.48975000	-1.66645700
N	-1.05038100	5.77240900	-0.75615400
C	-1.00481900	7.10726100	-2.75746800
C	-2.37367100	5.64300500	-0.90868200
H	-0.83626200	-6.08565300	4.76917000
H	-2.19243500	-5.76543800	2.68613700

H	-2.89056000	7.42856100	-3.73995000
C	-2.37565400	6.96532700	-2.90420100
C	4.84097500	4.86112500	0.15050000
C	3.65157800	6.79974600	-1.16146000
C	-4.33623500	3.47221100	1.92416200
C	-6.93044800	3.09969600	1.75717900
C	-5.15354500	-5.17380300	-2.00008700
C	-2.59962800	-4.57146800	-1.89434600
C	5.55724900	-4.34920100	2.35205900
C	6.65528200	-4.20412100	-0.02871700
C	4.26270900	5.84399000	-0.49979100
C	-5.63411100	3.29146100	1.84493600
C	-3.87440100	-4.87989200	-1.95080000
C	6.11885400	-4.28140400	1.16725800
N	0.84485600	-4.96159900	1.46106100
C	-3.07946200	6.22200100	-1.97024400
H	-4.15137200	6.08127800	-2.04443400
H	1.62890200	-5.65302200	4.66665100
H	-0.41315200	7.67696700	-3.46451300
C	2.97064700	-4.86489000	2.49375000
C	1.03693800	6.60870400	-1.48828200
C	-3.47143000	2.90872000	3.06682200
C	-7.97142300	4.06906600	2.34451400
C	4.37258900	8.02394700	-1.74987000
C	-5.69127500	-6.55031200	-2.42907300
C	-1.82406900	-3.95495800	-3.07301600
C	7.25231100	-5.41363600	-0.76895800
C	6.32375300	-4.13215300	3.66800500
C	5.00724300	4.83643400	1.68020400
C	6.21194700	-5.40467600	4.51991100
H	6.71745300	-5.26219900	5.48097800
H	5.16637200	-5.65677700	4.71901300
H	6.67558800	-6.25777200	4.01410900
C	5.68647700	-2.95030700	4.41368300
H	4.62645400	-3.13220000	4.61208500

H	6.19170000	-2.79306500	5.37267900
H	5.76801100	-2.02920000	3.82862500
C	7.79631300	-3.82568200	3.39052800
H	8.28707200	-4.65385800	2.86969700
H	7.90817300	-2.92697300	2.77693000
H	8.32478100	-3.66096800	4.33487200
C	8.73229400	-5.13024000	-1.06390100
H	9.17214000	-5.96136500	-1.62544200
H	8.85033200	-4.21821600	-1.65603600
H	9.30060800	-5.00711700	-0.13635300
C	6.48750400	-5.60498100	-2.08701600
H	6.54174800	-4.70774900	-2.71005500
H	6.91412100	-6.44008400	-2.65289900
H	5.43181100	-5.82279800	-1.89854900
C	7.12977100	-6.68044500	0.07932200
H	7.67420600	-6.58449100	1.02394300
H	6.08495700	-6.90514600	0.31298600
H	7.54767900	-7.53295500	-0.46546200
C	4.41074100	6.09770200	2.30674600
H	4.52636300	6.05991600	3.39454200
H	4.91272200	7.00125400	1.94609200
H	3.34423200	6.19005600	2.08134300
C	4.28205100	3.59759300	2.22690000
H	3.20990900	3.64628800	2.01308200
H	4.67580300	2.67867900	1.78402100
H	4.41166500	3.53681700	3.31278600
C	6.50392500	4.75010400	2.01216500
H	6.95770000	3.86100700	1.56546800
H	7.04016300	5.62990700	1.64141900
H	6.64642000	4.69679300	3.09672500
C	5.87349800	7.95432100	-1.46431900
H	6.37478800	8.82571300	-1.89749300
H	6.07642500	7.94814300	-0.38899900
H	6.32125900	7.05429300	-1.89618200
C	3.78451600	9.29381100	-1.11771900

H	3.95316600	9.30696700	-0.03623500
H	4.25593700	10.18418400	-1.54741000
H	2.70683200	9.36020900	-1.29225900
C	4.13994900	8.04611900	-3.26777300
H	4.56721300	7.15715000	-3.74232400
H	3.07239100	8.07590700	-3.50375900
H	4.61196700	8.93001600	-3.70987600
C	-7.28361800	5.26359600	3.00732700
H	-8.03662500	5.94970800	3.40793200
H	-6.64099200	4.94752900	3.83471100
H	-6.66528300	5.81589500	2.29350200
C	-8.81533800	3.31742300	3.38409500
H	-9.58897700	3.97668200	3.79211100
H	-9.30765700	2.44733900	2.94042800
H	-8.19349900	2.96714000	4.21407100
C	-8.87433600	4.56551200	1.20567100
H	-8.29550000	5.12166800	0.46161600
H	-9.36577700	3.73092700	0.69746900
H	-9.65052500	5.22962000	1.60067700
C	-4.53966800	-7.50080700	-2.76023900
H	-4.93752800	-8.47844700	-3.05039200
H	-3.93410200	-7.12323000	-3.58981600
H	-3.87925900	-7.64388600	-1.89986200
C	-6.52054800	-7.13314000	-1.27525700
H	-5.89975700	-7.28897100	-0.38742600
H	-7.34195600	-6.46516800	-1.00078800
H	-6.94792100	-8.09849700	-1.56682900
C	-6.58205700	-6.36839300	-3.66652800
H	-6.00713900	-5.96842100	-4.50782600
H	-7.00822400	-7.33024700	-3.97115100
H	-7.40670200	-5.67932100	-3.46274500
C	-1.26683500	-2.59405800	-2.62952000
H	-2.07624900	-1.90585700	-2.36689700
H	-0.61130100	-2.69768600	-1.76067600
H	-0.68609900	-2.14428000	-3.44178800

C	-2.74257400	-3.76282300	-4.28076700
H	-2.17966300	-3.31217800	-5.10419700
H	-3.14859000	-4.71658800	-4.63280300
H	-3.58308100	-3.10389800	-4.04340600
C	-0.66623800	-4.89258600	-3.44476100
H	0.00133900	-5.05413300	-2.59395600
H	-1.04041300	-5.86707400	-3.77575300
H	-0.07897700	-4.45835400	-4.26082200
C	-2.78515000	4.07730100	3.78874700
H	-3.52332900	4.74847600	4.24017200
H	-2.13794700	3.69754000	4.58642500
H	-2.16883600	4.66152200	3.09989300
C	-2.41012900	1.97963300	2.45876000
H	-2.87748100	1.13275800	1.94689300
H	-1.78370400	2.51131900	1.73736300
H	-1.76101700	1.58540400	3.24779900
C	-4.33276600	2.12307700	4.05684100
H	-3.70164100	1.71804300	4.85409800
H	-5.09389100	2.75895400	4.52033300
H	-4.84160100	1.28673400	3.56846100
C	6.68384100	-0.61169600	-1.95118400
C	7.09782300	-0.55486300	-3.28770200
C	7.05875400	0.66602600	-3.94172200
C	6.61199400	1.78326700	-3.25433000
C	6.22108200	1.63343100	-1.91841300
N	6.25855300	0.46125300	-1.27415600
H	7.37052500	0.74549200	-4.97839000
H	7.43417600	-1.45652400	-3.78611900
H	6.55875600	2.75805400	-3.72466500
C	-7.88146000	-2.31660300	-0.99731300
C	-9.23716900	-2.53828800	-1.26936400
C	-10.15128400	-1.55684600	-0.92125000
C	-9.69465600	-0.39612900	-0.31765500
C	-8.32091100	-0.26044400	-0.08239900
N	-7.42835800	-1.20004500	-0.41556500

H	-11.20968300	-1.69535900	-1.11829400
H	-9.54765200	-3.46247000	-1.74269900
H	-10.37219600	0.39829500	-0.02714900

5.2. Complexes

(P_2)-**2**·Catechol - [(P_2)-**2**·(**G1**)]

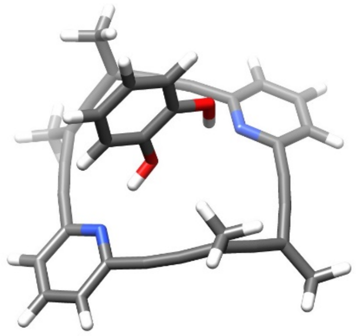

Figure S55. Optimized structure of a complex between (P_2)-**2** and catechol (**G1**). The tert-butyl groups were replaced by methyl groups.

N	-2.07724800	-1.97293300	-0.89507800
C	-3.29087800	-1.61957200	-1.34710900
C	-3.58005900	-0.21518100	-1.30534200
C	-3.67768000	0.99001800	-1.26632300
C	-3.68833700	2.42554700	-1.26761600
C	-2.90006600	3.04037300	-0.41270400
C	-2.04901100	3.57800500	0.43107400
C	-0.66768100	3.64436400	0.03974200
C	0.50177200	3.58998300	-0.26240900
C	1.86166000	3.33370700	-0.65436500
N	2.23418500	2.04697600	-0.60423300
C	3.46733300	1.71582700	-1.01240300
C	3.76111200	0.31036500	-1.00173800
C	3.86284300	-0.89493100	-1.01304700
C	3.87558400	-2.32845700	-1.08646500
C	3.02254200	-2.98588100	-0.33184000
C	2.10771700	-3.56775300	0.41004600
C	0.76278100	-3.61110500	-0.09491500
C	-0.37999500	-3.54249800	-0.48427100
C	-1.71126000	-3.26356900	-0.94677100

C	-2.56103300	-4.25942800	-1.43270300
C	-4.19944400	-2.55558800	-1.84850100
C	-3.82230000	-3.89098300	-1.88249200
C	2.72070900	4.34829200	-1.08401600
C	4.39107500	2.66700800	-1.45660600
C	4.00497800	4.00001400	-1.48184600
H	-2.22914300	-5.29057600	-1.45555400
H	-5.17137300	-2.23141700	-2.20128600
H	-4.50641300	-4.64149100	-2.26463900
H	2.37954700	5.37661700	-1.10552100
H	5.37994700	2.35719300	-1.77394800
H	4.69854000	4.76335000	-1.81899900
C	-1.58544700	-0.98521100	3.85208700
C	-0.58268300	-0.37502800	4.59738100
C	0.45507900	0.29385700	3.94757500
C	0.49539700	0.35095200	2.56028900
C	-0.51390100	-0.26858700	1.80419800
C	-1.55111100	-0.92774600	2.45766800
H	-2.40005900	-1.50527800	4.34648700
H	-0.60213500	-0.41349600	5.68207000
H	1.24937000	0.77801700	4.50763000
H	-2.33289500	-1.39704500	1.86908900
O	1.52097500	1.00193600	1.95633500
O	-0.39914800	-0.16336300	0.45672700
H	-1.01536900	-0.77393000	-0.01980800
H	1.42254300	1.03362600	0.98240000
C	-2.42393000	4.04619600	1.81866200
H	-1.85903600	3.48314800	2.56753800
H	-2.18053200	5.10621600	1.93903800
H	-3.49092900	3.90471700	1.99819000
C	-4.54555300	3.13879600	-2.28842900
H	-4.26597800	2.83004400	-3.30024000
H	-5.60018300	2.88731500	-2.13986600
H	-4.42601200	4.22001200	-2.20269100
C	2.36821300	-4.10975400	1.79719100
H	1.74218000	-3.58697900	2.52641200
H	2.12140900	-5.17493600	1.84129900
H	3.41617800	-3.97711600	2.07050100

C	4.81222900	-2.99179900	-2.07030800
H	4.61296400	-2.63109300	-3.08392800
H	5.85097900	-2.74931200	-1.82609500
H	4.68928900	-4.07585200	-2.04955700

(P_2)-**2**·Resorcinol-[(P_2)-**2**·(**G3**)$_2$]

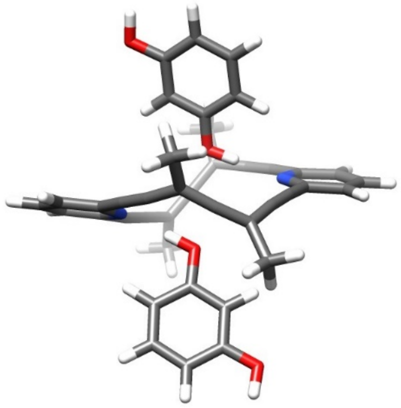

Figure S56. Optimized structure of a complex between (P_2)-**2** and two molecules of resorcinol (**G3**). The tert-butyl groups were replaced by methyl groups.

N	1.29731100	-2.79335600	-0.30673900
C	0.43267400	-3.63973000	0.30234200
C	-0.64444400	-3.10343700	1.09056800
C	-1.59709400	-2.58190500	1.62825700
C	-2.79977900	-1.96694700	2.11732100
C	-2.69275400	-0.81496300	2.73029200
C	-2.46759700	0.32584000	3.32924700
C	-2.22624400	1.46378300	2.49701700
C	-1.86132500	2.36764300	1.79821800
C	-1.30311500	3.28609800	0.84205400
N	-0.70325700	2.70163700	-0.20921400
C	-0.03705200	3.47445700	-1.09200200
C	0.70806200	2.82221500	-2.13412800
C	1.41087000	2.22097600	-2.91456900
C	2.23683900	1.47979500	-3.82582300
C	3.14241400	0.67953700	-3.31037200
C	4.00877300	-0.11241300	-2.71657800
C	3.52220900	-1.38214700	-2.28415000
C	3.00368500	-2.34037000	-1.78912100
C	2.24097400	-3.32204200	-1.09222900

C	2.42417100	-4.69230800	-1.27170100
C	0.53227800	-5.03014300	0.14890600
C	1.54935500	-5.55545700	-0.63294200
C	-1.33911800	4.67091500	1.01424000
C	-0.01226900	4.86979400	-0.97391300
C	-0.68777100	5.46629700	0.08222100
H	3.21314600	-5.05729600	-1.91900000
H	-0.18478800	-5.67319100	0.64677400
H	1.64988100	-6.62860400	-0.75459200
H	-1.85034700	5.09734800	1.86801700
H	0.52930000	5.46075400	-1.70242900
H	-0.68767200	6.54660100	0.19039300
C	3.33028100	-0.93645800	3.63193000
C	3.77666400	0.31332600	4.04643300
C	3.58530300	1.40502700	3.19449100
C	2.95229200	1.25514100	1.96543400
C	2.50477000	-0.00973200	1.56633500
C	2.69872500	-1.11361500	2.40803900
H	3.47519600	-1.79507700	4.28227600
H	4.26576700	0.43961300	5.00595300
H	2.36154800	-2.09707700	2.09940700
O	1.89839600	-0.12577900	0.37105100
H	1.63413000	-1.06522100	0.21978100
C	-2.41239700	0.50147900	4.82974000
H	-1.42503200	0.86672500	5.12747800
H	-3.15623700	1.23713700	5.15126600
H	-2.60737700	-0.44380500	5.33750900
C	-4.12182200	-2.63192200	1.79323100
H	-4.23850700	-2.72588600	0.70856100
H	-4.15762600	-3.63621600	2.22543600
H	-4.95374400	-2.04670000	2.18850800
C	5.44988900	0.23984200	-2.42489300
H	5.64089200	0.18709600	-1.34877700
H	6.11781800	-0.47184000	-2.92004000
H	5.68048500	1.24618700	-2.77715100
C	1.97470800	1.59497300	-5.30939500
H	0.93913200	1.32386700	-5.53345000
H	2.12995600	2.62646000	-5.64008800

H	2.64361400	0.93931700	-5.86960600
H	2.80189300	2.10837700	1.31623100
O	4.00361000	2.66429500	3.53076800
H	4.43759300	2.64704800	4.39561200
C	-5.00621200	1.26979800	-1.89390300
C	-5.63465100	0.06517500	-2.19772200
C	-4.91841500	-1.12164200	-2.00283600
C	-3.62516200	-1.10742900	-1.49754900
C	-3.01016100	0.11064000	-1.19492000
C	-3.70764600	1.31033700	-1.39867500
H	-5.54522200	2.20153100	-2.04091500
H	-6.65687000	0.03341100	-2.57770100
H	-3.23926000	2.26009100	-1.16355900
O	-1.75446900	0.05904500	-0.71143700
H	-1.40505000	0.96413200	-0.48293300
H	-3.08253600	-2.03511300	-1.35326100
O	-5.44879200	-2.34744900	-2.29238200
H	-6.35784500	-2.25110400	-2.60539500

[(P_2)-**2**·(**G6**)]

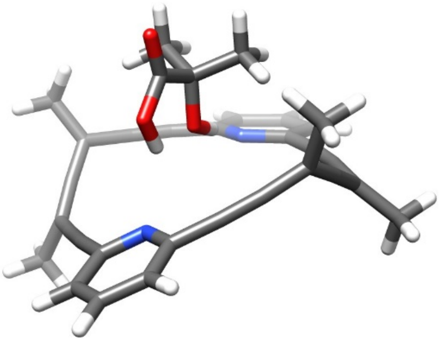

Figure S57. Optimized structure of a complex between (P_2)-**2** and **G6**. The tert-butyl groups were replaced by methyl groups.

N	-1.08356500	2.76221800	-0.65065700
C	-0.28371300	3.80449300	-0.91438100
C	1.12293700	3.51276200	-0.95795600
C	2.27632300	3.15430700	-1.02181100
C	3.60473100	2.63269500	-1.18076900
C	4.00404200	1.67850100	-0.36980700
C	4.32680200	0.67769600	0.41856700

C	3.82972600	-0.62452700	0.06773300
C	3.30668900	-1.68123800	-0.20098100
C	2.53660800	-2.84086500	-0.55633200
N	1.20454300	-2.66925900	-0.56673300
C	0.42572400	-3.68614700	-0.96920300
C	-0.97922800	-3.40552400	-1.05964100
C	-2.12976600	-3.05242900	-1.18204100
C	-3.45237000	-2.54050000	-1.40677400
C	-3.89116600	-1.58156300	-0.62142000
C	-4.24660400	-0.58074300	0.15234700
C	-3.73573900	0.72378200	-0.16702500
C	-3.19981400	1.78509700	-0.38715100
C	-2.40981500	2.95777500	-0.64820300
C	-2.98923400	4.20671600	-0.88747400
C	-0.77733000	5.08931000	-1.16265600
C	-2.15139800	5.28414200	-1.14236000
C	3.13854700	-4.05363900	-0.90005000
C	0.94912100	-4.93220600	-1.32763900
C	2.32424000	-5.11229800	-1.27922200
H	-4.06716500	4.31771300	-0.87213900
H	-0.09254700	5.90430000	-1.36622100
H	-2.56733300	6.26895400	-1.32825300
H	4.21762000	-4.14886400	-0.87360700
H	0.28398700	-5.72828200	-1.64085300
H	2.76052300	-6.06833700	-1.54930700
C	5.11873000	0.82959300	1.69701700
H	4.53546800	0.47186100	2.55059500
H	6.03670600	0.23593500	1.64597200
H	5.38350300	1.87505300	1.86287300
C	4.44295100	3.15819400	-2.32496500
H	3.92410800	3.00130600	-3.27538300
H	4.61088700	4.23307800	-2.20731000
H	5.40909400	2.65219100	-2.36006400
C	-5.08544000	-0.73809900	1.40016800
H	-4.53812700	-0.36997300	2.27299800
H	-6.00522700	-0.15172000	1.31208800
H	-5.34857400	-1.78515700	1.55926200
C	-4.23208400	-3.07987500	-2.58529400

H	-3.67073100	-2.92656900	-3.51184300
H	-4.40069100	-4.15474200	-2.46811100
H	-5.19777000	-2.57886800	-2.66914900
C	-0.04050300	-0.68425000	2.27527400
C	1.31100300	-0.99227500	2.92219300
H	1.19792900	-1.12917100	4.00004700
H	1.72192400	-1.91104700	2.49489300
H	2.01739200	-0.17802300	2.74167700
C	-1.04349900	-1.81193600	2.52070400
H	-1.99916300	-1.58852900	2.03997700
H	-0.65549400	-2.74420500	2.10097200
H	-1.20886300	-1.95433800	3.59104600
C	-0.58482400	0.60679200	2.89647100
O	-0.82890200	0.70737900	4.08190300
O	-0.77290500	1.62938900	2.07099200
H	-0.55387000	1.37046000	1.14824400
O	0.09962900	-0.41235600	0.88979000
H	0.46312800	-1.18147000	0.39789900

[(*P*$_2$)-**2**·(*R*)-**G7**)]

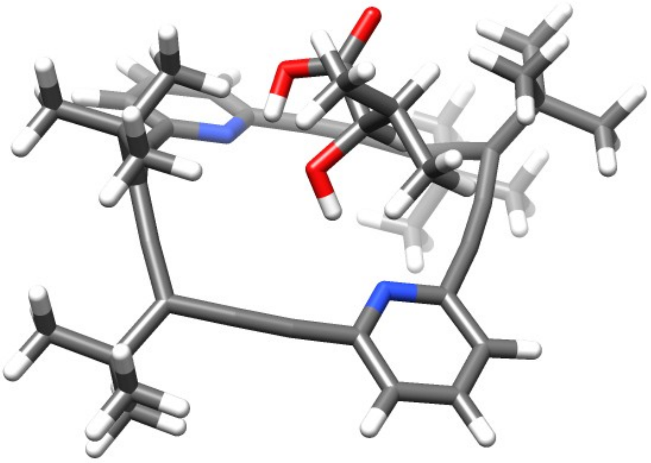

Figure S58. Optimized structure of a complex between (*P*$_2$)-**2** and (*R*)-**G7**.

N	0.13386900	3.14088900	-0.57529400
C	1.30387100	3.76006900	-0.76930600
C	2.44924700	2.89912000	-0.84912600
C	3.33824500	2.08199800	-0.91882900
C	4.31402400	1.04568200	-1.08112700
C	4.18225300	-0.04244700	-0.35751200

C	3.99706400	-1.15311000	0.31938400
C	3.02559300	-2.06196900	-0.22101700
C	2.11349200	-2.73132900	-0.65126700
C	0.93527900	-3.39001000	-1.13522100
N	-0.20681000	-2.69130100	-1.03450300
C	-1.34054500	-3.25187400	-1.48483400
C	-2.51440600	-2.43966600	-1.38128700
C	-3.41659600	-1.63815900	-1.28995500
C	-4.42708700	-0.62542500	-1.21849600
C	-4.21726900	0.35953900	-0.37377000
C	-3.93801800	1.31542100	0.47969800
C	-3.03512100	2.34704900	0.06305300
C	-2.16812800	3.14447500	-0.21216300
C	-0.97101300	3.89128100	-0.48339000
C	-0.96023700	5.28385600	-0.60497400
C	1.41507700	5.14967400	-0.89847200
C	0.25911600	5.91409600	-0.81840100
C	0.98547600	-4.67818800	-1.67560700
C	-1.37892900	-4.53719100	-2.03705900
C	-0.19486600	-5.25435500	-2.12644000
H	-1.88445300	5.84383800	-0.52566500
H	2.38694200	5.60334300	-1.05294600
H	0.30863000	6.99368600	-0.91438400
H	1.93257600	-5.20075300	-1.73305300
H	-2.32082700	-4.94704300	-2.38109100
H	-0.19034900	-6.25424300	-2.54731600
C	4.72579400	-1.50715800	1.62340300
C	5.41689900	1.22725600	-2.13558300
C	-4.44288400	1.33188200	1.93089300
C	-5.64596000	-0.72046100	-2.14568700
C	-0.03442700	-1.86223300	2.93811300
C	0.30073900	-0.80167900	1.87512600
C	-1.29420300	-1.50519300	3.72519100
H	-1.49611700	-2.26138500	4.48998100
H	-1.19148700	-0.53962800	4.22944700
H	-2.15948200	-1.45841300	3.05668600
C	-0.16578400	-3.24334200	2.29791100
H	-0.99328900	-3.25581500	1.58091400

H	0.74825400	-3.52655900	1.76541800
H	-0.36557100	-4.00465200	3.05813600
C	0.66012900	0.53720500	2.52452800
O	0.12354800	1.60653600	1.95099800
H	-0.37947300	1.30483400	1.17017600
O	1.39688800	0.61972400	3.48209000
C	-3.23767300	1.53455600	2.86102800
H	-2.77744300	2.51472400	2.71369900
H	-3.55306400	1.45548800	3.90635100
H	-2.47382900	0.77937500	2.66751200
C	-5.43237800	2.49752500	2.08350300
H	-6.30856700	2.35500300	1.44269700
H	-5.77255400	2.56857500	3.12186800
H	-4.96133300	3.44803300	1.81462800
C	-5.13571100	0.00688700	2.26000800
H	-5.99228500	-0.17244600	1.60172800
H	-4.44400600	-0.83429700	2.15416500
H	-5.49877200	0.02560000	3.29192700
C	3.70480700	-1.88781400	2.70576400
H	3.06656200	-2.71439600	2.37589800
H	4.23093900	-2.21197100	3.60963800
H	3.06879800	-1.03841000	2.97315900
C	5.55365100	-0.31059500	2.09946200
H	6.29884200	-0.01596700	1.35315300
H	4.91245400	0.55142600	2.30473000
H	6.08129300	-0.57092500	3.02185900
C	5.64616300	-2.70323000	1.32689100
H	6.38501500	-2.44842900	0.56026900
H	6.18015900	-3.00089200	2.23511800
H	5.06992000	-3.56460200	0.97412100
C	-6.57557500	0.47463700	-1.91894300
H	-6.93801800	0.50539600	-0.88643200
H	-6.06478200	1.41980200	-2.12563000
H	-7.44284100	0.40161800	-2.58221100
C	-6.39097300	-2.02761800	-1.83157900
H	-7.25502400	-2.13883500	-2.49476200
H	-5.73914800	-2.89539000	-1.97134800
H	-6.74829600	-2.03338900	-0.79708200

C	-5.15927900	-0.73302900	-3.60331100
H	-4.62813800	0.19278100	-3.84349800
H	-4.48017600	-1.57139200	-3.78480600
H	-6.01103000	-0.82902900	-4.28460200
C	6.23925700	2.47035600	-1.76090800
H	7.01955800	2.64570300	-2.50873000
H	5.60501600	3.36045700	-1.71239400
H	6.71948300	2.34052100	-0.78606300
C	6.32605200	-0.00412700	-2.17488400
H	6.81382100	-0.16948000	-1.20883100
H	5.76238400	-0.90700100	-2.42850100
H	7.10594300	0.13546800	-2.92966200
C	4.75675600	1.42960700	-3.50851000
H	4.16825500	0.55111500	-3.78963200
H	4.09038500	2.29679300	-3.50116900
H	5.52279800	1.59142700	-4.27394900
O	-0.74280800	-0.58651100	0.94667800
H	-0.57548800	-1.14417400	0.16560300
C	1.57525030	-1.25347361	1.13817107
H	2.28616771	-1.62166211	1.84805393
H	1.32961253	-2.02975094	0.44394086
H	1.99550738	-0.42291684	0.61047851
C	1.16206270	-1.87129814	3.90761299
H	0.98991284	-1.16553859	4.69321333
H	1.27501444	-2.84977567	4.32561253
H	2.05258776	-1.60487863	3.37762642

[(*P*$_2$)-**2**·(*S*)-**G7**)]

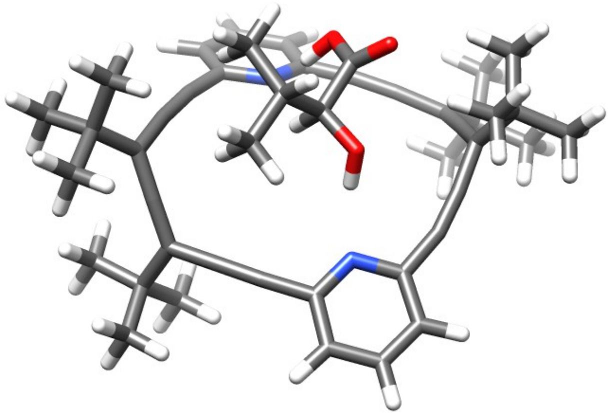

Figure S59. Optimized structure of a complex between (*P*$_2$)-**2** and (S)-**G7**.

N	0.23469600	-2.90023500	-0.48614400
C	-0.81304400	-3.51571800	-1.06076000
C	-2.04737000	-2.79154600	-1.04716700
C	-3.00970000	-2.05723400	-1.04358400
C	-4.11262000	-1.14848500	-1.13035000
C	-4.15137600	-0.11291300	-0.32045800
C	-4.18983900	0.96109800	0.43537500
C	-3.38740200	2.06791400	0.00674800
C	-2.61429200	2.94563500	-0.30318900
C	-1.45563900	3.74187300	-0.58238700
N	-0.29249300	3.07677900	-0.48646500
C	0.84499300	3.73003400	-0.75947100
C	2.04162800	2.93704200	-0.74943500
C	2.98290500	2.18061200	-0.83023600
C	4.01779400	1.22521900	-1.09498800
C	4.20667800	0.22048500	-0.27142500
C	4.35839600	-0.85444200	0.47009100
C	3.41892400	-1.90809700	0.20313400
C	2.54201300	-2.69479800	-0.07667400
C	1.44239500	-3.48131800	-0.56032900
C	1.64048900	-4.73297100	-1.14963400
C	-0.69886200	-4.76715800	-1.67774700
C	0.54378000	-5.38342500	-1.70149900
C	-1.52090600	5.09464700	-0.92954100
C	0.87131400	5.08537200	-1.10482000
C	-0.33446900	5.77021900	-1.18146600
H	2.63328700	-5.16523000	-1.17876700
H	-1.57225400	-5.22918900	-2.12169800
H	0.66205400	-6.35785900	-2.16362100
H	-2.48255700	5.58925500	-0.99368200
H	1.81688400	5.57261900	-1.31045400
H	-0.34941400	6.82244700	-1.44552700
C	-5.02010100	1.08754700	1.72340800
C	-5.16669500	-1.39761500	-2.22429300
C	5.46279000	-1.03270000	1.51766600
C	4.80334400	1.37144700	-2.41292000
C	0.82225700	0.15406400	3.12603500

C	-0.20145400	0.09727300	1.97313700
O	-0.78647600	1.35945300	1.80221900
H	-0.57445700	1.70093700	0.91954900
C	1.36478000	-1.22885500	3.48942100
H	2.11411500	-1.14810000	4.28256900
H	0.57758700	-1.89802800	3.84633900
H	1.84430000	-1.70086400	2.62377200
C	1.94026900	1.13158600	2.77027600
H	2.53366900	0.75547000	1.92876400
H	1.52286500	2.10008500	2.48569900
H	2.61415800	1.28157200	3.62086300
C	-1.28770400	-0.91835900	2.34686900
O	-1.15980000	-2.14247800	1.83091300
H	-0.49810900	-2.16309800	1.11070600
O	-2.16761500	-0.67338900	3.13734700
C	4.85181700	-1.47164400	2.85402200
H	4.26639700	-2.38920600	2.74336700
H	5.64645100	-1.65921700	3.58331600
H	4.19348500	-0.69657000	3.25343000
C	6.41761700	-2.12140200	0.99847200
H	6.86928800	-1.82387900	0.04687700
H	7.22055700	-2.29427500	1.72236700
H	5.88848100	-3.06710400	0.84501800
C	6.22170300	0.28351700	1.70700200
H	6.68701800	0.61643200	0.77380300
H	5.55204200	1.07633400	2.05408700
H	7.01211300	0.15162100	2.45192000
C	-4.21105600	1.83917400	2.79049700
H	-3.99396900	2.86271200	2.47019600
H	-4.79577600	1.88944200	3.71515600
H	-3.26677800	1.33072200	2.99591700
C	-5.39823200	-0.30497600	2.23648300
H	-5.95556300	-0.87162200	1.48220400
H	-4.50297200	-0.86698600	2.51544100
H	-6.03484100	-0.20730800	3.12150900
C	-6.28616400	1.88738800	1.36883700
H	-6.89773300	1.35552000	0.63269500
H	-6.89035500	2.04553300	2.26836100

H	-6.03002700	2.86895700	0.95637200
C	5.83045400	0.24419300	-2.55213300
H	6.55728500	0.26313000	-1.73369800
H	5.34744600	-0.73749700	-2.55456400
H	6.37705900	0.35848700	-3.49306400
C	5.52195600	2.72963100	-2.39799400
H	6.07027400	2.87549600	-3.33430700
H	4.80789200	3.55113500	-2.28869900
H	6.23552400	2.78452000	-1.56985900
C	3.81279700	1.31309600	-3.58658000
H	3.28965200	0.35240900	-3.60529800
H	3.06404000	2.10644400	-3.51274800
H	4.34759700	1.43360100	-4.53431400
C	-5.77876800	-2.78931800	-1.99984800
H	-6.52057000	-3.00441500	-2.77603400
H	-5.01117800	-3.56811700	-2.03499000
H	-6.27405600	-2.84409000	-1.02555000
C	-6.26646900	-0.33488200	-2.15733400
H	-6.78313900	-0.36148700	-1.19280300
H	-5.85912300	0.67108400	-2.29482500
H	-7.00470800	-0.51656700	-2.94448200
C	-4.47575200	-1.34393700	-3.59605500
H	-4.04008500	-0.35585000	-3.77231800
H	-3.67473900	-2.08605100	-3.66320300
H	-5.19971400	-1.54888800	-4.39165300
C	0.51744059	-0.36693818	0.69278605
H	-0.20674457	-0.58046154	-0.06541156
H	1.17346821	0.40649552	0.35171703
H	1.08508964	-1.24938477	0.90245861
C	0.03888845	0.71313487	4.32827187
H	-0.48996787	-0.08230369	4.81044248
H	0.72054740	1.15993705	5.02153148
H	-0.65820251	1.45021645	3.98816168

[(P_3)-**3**·(**G13**)]$^+$

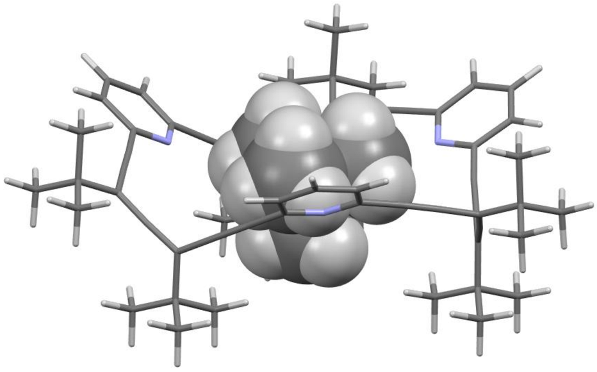

Figure S60. Optimized structure of a complex between (P_3)-**3** and tetramethylammonium (**G13**).

C	2.47123500	-6.67327000	-2.12646500
C	1.09420700	-6.60081200	-1.96153100
C	0.55468900	-5.54705000	-1.21071100
N	1.33031100	-4.59093700	-0.65110200
C	2.67050100	-4.68308600	-0.79516400
C	3.27632500	-5.70944200	-1.53108400
C	3.47428300	-3.69591900	-0.14151900
C	4.20881800	-2.90661700	0.41198100
C	5.06903500	-1.96596100	1.07347500
C	6.41089200	-0.27470500	-0.43031000
C	5.74845100	0.95623100	-0.74753500
C	5.16996300	1.99405400	-0.98520800
C	3.30214300	5.67893600	-1.56476700
C	4.53730100	5.46058200	-2.16334800
C	5.16664900	4.23487200	-1.98922900
C	4.52935800	3.24666100	-1.22605400
N	3.31535600	3.44292600	-0.66339900
C	2.72173300	4.64689300	-0.81639900
C	1.46744200	4.85252100	-0.15893600
C	0.41897800	5.09623700	0.39808900
C	-0.81621200	5.41459900	2.60627600
C	-3.46747800	7.02628200	-1.00221100
C	-0.82229400	5.37470000	1.06444900
C	-2.96561000	5.68726500	-0.42905700
C	-3.70444200	4.49796000	-0.73599600

C	-4.31791000	3.47858300	-0.96569700
C	-6.59167100	0.02589600	-1.52410600
C	-7.02446700	1.20739200	-2.11428500
C	-6.27220300	2.36266500	-1.94676400
C	-5.08832900	2.29976100	-1.19860600
N	-4.64747800	1.14777000	-0.64413300
C	-5.39833600	0.03415800	-0.79068400
C	-4.94454100	-1.15815400	-0.14232600
C	-4.62755700	-2.19083700	0.40724000
C	-4.24368600	-3.40819900	1.06533500
C	-3.44066200	-5.40973200	-0.44172900
C	-2.04147800	-5.45144900	-0.75017900
C	-0.85183300	-5.47107400	-0.97963100
C	5.77556400	-1.13445600	0.33820100
C	-1.89907900	5.56923500	0.33378200
C	-3.87234400	-4.43260200	0.32787400
H	2.91310400	-7.47735500	-2.70224600
H	0.43507000	-7.34228200	-2.39345700
H	4.35415800	-5.74174100	-1.61956300
H	2.78876300	6.62653800	-1.66008900
H	5.00847600	6.24073300	-2.74874700
H	6.13694600	4.03290000	-2.42343300
H	-7.15910500	-0.89083300	-1.61475200
H	-7.94289900	1.22935200	-2.68815700
H	-6.58520100	3.30588400	-2.37474700
C	-4.27957100	-3.43272000	2.60716200
C	-4.34857000	-6.51083700	-1.02213100
C	5.09984200	-1.98205000	2.61543800
C	7.82264600	-0.51134300	-0.99949500
C	-4.25118700	-6.46904500	-2.55976400
H	-3.21972100	-6.61078100	-2.89529100
H	-4.86354700	-7.26516500	-2.99464300
H	-4.61006000	-5.51081000	-2.94802700
C	-3.85924200	-7.87810700	-0.50600800
H	-2.81810600	-8.05891000	-0.78871700
H	-3.93219400	-7.93322900	0.58447100
H	-4.47225800	-8.67925900	-0.93074100
C	-5.80431100	-6.28768500	-0.59266100

H	-5.90829000	-6.32181900	0.49618400
H	-6.17833400	-5.32017500	-0.94107900
H	-6.43868400	-7.07101800	-1.01741600
C	-3.31350900	-2.35366900	3.13639700
H	-3.58335100	-1.36310300	2.75766400
H	-3.34859100	-2.32236200	4.22990600
H	-2.28381600	-2.57953300	2.83611500
C	-5.71333200	-3.12065000	3.07729800
H	-5.75551500	-3.11006200	4.17103700
H	-6.04703100	-2.14485800	2.71293200
H	-6.41430400	-3.87932500	2.71635400
C	-3.84962800	-4.80803300	3.13315100
H	-4.52410900	-5.59511800	2.78302200
H	-2.83545700	-5.06088100	2.80939600
H	-3.86935900	-4.80820700	4.22692900
C	-3.50018900	6.92324900	-2.53967100
H	-4.14723300	6.10471000	-2.86804800
H	-3.88433100	7.85438900	-2.96782300
H	-2.49685300	6.75063500	-2.94118600
C	-2.53662700	8.17164200	-0.58367500
H	-2.49870300	8.27735400	0.50487100
H	-1.51716200	8.00850700	-0.94634100
H	-2.90029600	9.11444600	-1.00235500
C	-4.88861500	7.29068200	-0.46749100
H	-5.57240900	6.48262000	-0.74323000
H	-4.88552800	7.37922100	0.62323200
H	-5.27774400	8.22451400	-0.88531000
C	-2.21770500	5.73447500	3.14172400
H	-2.56015700	6.71451100	2.79630800
H	-2.94901800	4.98666400	2.82045500
H	-2.20097800	5.74867300	4.23544600
C	0.17808600	6.49518000	3.07314200
H	0.21571800	6.52300400	4.16673300
H	1.18694300	6.29384500	2.70168600
H	-0.12724100	7.48378900	2.71731000
C	-0.36708000	4.03555100	3.12987200
H	-1.08402900	3.26058300	2.83506900
H	0.62115600	3.76950800	2.74234500

H	-0.31292200	4.04871400	4.22296200
C	6.07174100	-0.92001000	3.14500400
H	7.09250500	-1.10915100	2.79959900
H	5.78347300	0.08400600	2.81921000
H	6.07693000	-0.93627800	4.23881800
C	5.54597300	-3.37836300	3.09023000
H	5.55183500	-3.41864900	4.18406800
H	4.87112100	-4.15695000	2.72324700
H	6.55597700	-3.60487700	2.73548900
C	3.67909500	-1.68613400	3.13666300
H	3.36011900	-0.68238800	2.83301100
H	2.95935000	-2.41676400	2.75500600
H	3.66377100	-1.73048900	4.23015100
C	7.75068200	-0.44922800	-2.53782900
H	7.36146400	0.51490200	-2.87772900
H	8.74994100	-0.58269100	-2.96395600
H	7.10292600	-1.23898100	-2.93080800
C	8.75788000	0.59636300	-0.47638900
H	9.76209300	0.46503400	-0.89154500
H	8.39705900	1.58825100	-0.76377200
H	8.83189400	0.56219500	0.61487300
C	8.35339100	-1.88355200	-0.56486300
H	8.42670800	-1.95611000	0.52460600
H	7.70463800	-2.69104100	-0.91769600
H	9.35202400	-2.04203500	-0.98203400
C	0.00115100	0.00077500	1.37832400
N	-0.00207200	-0.00159000	-0.12900300
C	-1.39684000	-0.31632000	-0.62655200
C	0.42164000	1.36170700	-0.63305900
C	0.96565000	-1.05237900	-0.63033400
H	1.01035000	0.21646900	1.72786900
H	-0.31621300	-0.97957200	1.73235300
H	-0.68840200	0.76733500	1.73060200
H	-1.69698700	-1.29138800	-0.24391200
H	-2.10519100	0.44480800	-0.29187000
H	-1.37569800	-0.33946000	-1.71587800
H	0.38934900	1.35028400	-1.72229000
H	1.43540100	1.59603300	-0.30072500

H	-0.27245800	2.11046000	-0.25247000
H	1.96191300	-0.82417700	-0.25261600
H	0.66259000	-2.04623600	-0.29333000
H	0.97005200	-1.02368900	-1.71976300

[(P_3)-**3**·(**G14**)]$^+$

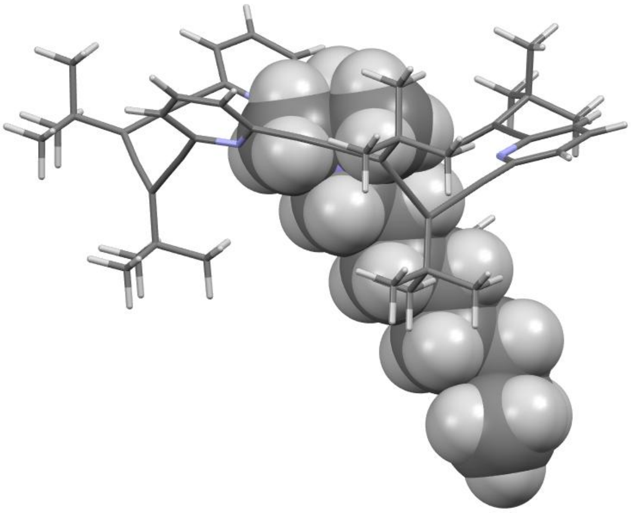

Figure S61. Optimized structure of a complex between (P_3)-**3** and cetrimonium (**G14**). Alkyl chain was shortened to 8 carbon atoms.

C	6.79409700	-0.51662600	-2.56456900
C	6.02019000	-1.66841600	-2.52390700
C	4.75872300	-1.62024900	-1.91362100
N	4.26544000	-0.48325900	-1.37195000
C	5.03158500	0.63038500	-1.40072600
C	6.30121300	0.64945700	-1.99182700
C	4.51360500	1.81781100	-0.79318600
C	4.16053700	2.86696600	-0.29941400
C	3.74662000	4.11528600	0.27870700
C	2.49643000	5.82959200	-1.27782000
C	1.06815200	5.72894800	-1.34270300
C	-0.14020100	5.64023100	-1.36366600
C	-4.30511400	5.50978800	-1.16677900
C	-3.70766000	6.54597100	-1.87429600
C	-2.32222900	6.59687400	-1.96060300

C	-1.56697100	5.59194700	-1.34084300
N	-2.14257000	4.56702500	-0.67279700
C	-3.48965200	4.53671800	-0.57424000
C	-4.07283600	3.47026800	0.17999000
C	-4.62327700	2.60215100	0.82228000
C	-4.90737600	1.42469500	3.06718900
C	-8.38681400	0.14768600	0.05932800
C	-5.26365400	1.55912800	1.57336900
C	-6.87221100	-0.05059400	0.25346900
C	-6.24946200	-1.18825600	-0.35738900
C	-5.68330400	-2.13909700	-0.85048700
C	-3.72070300	-5.51311000	-2.32538800
C	-5.03221500	-5.28357000	-2.72538100
C	-5.69903000	-4.16129700	-2.25080800
C	-5.02255100	-3.28221300	-1.39336400
N	-3.73922900	-3.48616300	-1.02076300
C	-3.10762800	-4.59355900	-1.46505000
C	-1.77624600	-4.81892100	-0.98972900
C	-0.66413100	-5.06476400	-0.57633100
C	0.64159900	-5.36228500	-0.05828800
C	2.69956900	-5.12222100	-1.68088600
C	3.37403000	-3.86036700	-1.76068400
C	3.96753400	-2.80563800	-1.83030600
C	3.14306700	4.99412900	-0.49203900
C	-6.10569700	0.76501800	0.94694100
C	1.68101000	-5.25330700	-0.85729700
H	7.77212100	-0.52861700	-3.02995700
H	6.37345100	-2.59953100	-2.94685200
H	6.87739700	1.56509800	-1.99168300
H	-5.37997300	5.44495200	-1.06159200
H	-4.31463200	7.31065900	-2.34355700
H	-1.81940900	7.39719300	-2.48736500
H	-3.17447900	-6.38629600	-2.65704700
H	-5.53199600	-5.97786800	-3.38991100
H	-6.72612800	-3.95850900	-2.52452100
C	0.73907400	-5.81890000	1.41279800
C	3.22257400	-6.26990200	-2.57119200
C	4.04524900	4.35068900	1.77347100

C	3.18723000	6.91900900	-2.12117500
C	3.14132000	-5.82740500	-4.04542100
H	3.72651000	-4.92006000	-4.22081800
H	3.53403100	-6.61682000	-4.69394200
H	2.10511900	-5.62991200	-4.33706500
C	4.68805600	-6.56112300	-2.19389400
H	5.31456300	-5.67354900	-2.32169600
H	4.76591800	-6.88823400	-1.15253000
H	5.08785700	-7.35518600	-2.83238900
C	2.37841500	-7.53495200	-2.36959900
H	2.42604300	-7.88635400	-1.33434300
H	1.32849300	-7.35784100	-2.62136200
H	2.75350100	-8.33423300	-3.01533100
C	0.11400200	-4.73716500	2.31502200
H	-0.92691800	-4.54432300	2.04032900
H	0.13653600	-5.06411500	3.35932700
H	0.67280500	-3.79786400	2.24191900
C	-0.04492700	-7.13719700	1.56815900
H	-0.01081300	-7.47245700	2.60963900
H	-1.09359300	-7.00996700	1.28477400
H	0.38713200	-7.92366300	0.94187800
C	2.20395500	-6.03880700	1.81188000
H	2.67244600	-6.81455900	1.19883900
H	2.78990200	-5.12066600	1.70239600
H	2.25769200	-6.35612500	2.85735000
C	-8.67825500	0.26327300	-1.44983900
H	-8.34048600	-0.62754800	-1.98728900
H	-9.75455000	0.37415600	-1.61492300
H	-8.17449700	1.13485800	-1.87914700
C	-8.85731700	1.42135000	0.77323200
H	-8.67101300	1.36620600	1.85022000
H	-8.34685800	2.30749200	0.38391700
H	-9.93259900	1.55379500	0.62236000
C	-9.12336000	-1.07535800	0.63974600
H	-8.79620100	-2.00050500	0.15640800
H	-8.94033600	-1.16781500	1.71468400
H	-10.20182500	-0.97146100	0.48419700
C	-5.67542100	0.25717700	3.69962500

H	-6.75642200	0.41418800	3.63786400
H	-5.44080600	-0.69008100	3.20479000
H	-5.40704500	0.16698000	4.75637900
C	-5.27199800	2.73796700	3.78605200
H	-4.99939400	2.67484700	4.84437200
H	-4.74482600	3.59009500	3.34735500
H	-6.34724200	2.93008100	3.72156300
C	-3.39134000	1.17011800	3.18938800
H	-3.12031000	0.22420700	2.70649000
H	-2.81740800	1.97993900	2.72841400
H	-3.10588800	1.10701800	4.24421700
C	3.51186700	5.71831400	2.21863500
H	3.98839700	6.53125600	1.66260000
H	2.43046500	5.79271500	2.06955200
H	3.72143800	5.86802800	3.28179800
C	5.56993000	4.29134900	1.98996700
H	5.80388600	4.42853500	3.05043400
H	5.97872000	3.32748100	1.67282200
H	6.07409400	5.08088300	1.42450900
C	3.36096200	3.23894900	2.59345100
H	2.27366700	3.27686900	2.46559800
H	3.71549800	2.24945300	2.28849700
H	3.58271500	3.36783400	3.65749200
C	2.84072600	6.68696700	-3.60498100
H	1.75922700	6.71034100	-3.76734700
H	3.29511100	7.46908900	-4.22119500
H	3.21909300	5.71893100	-3.94790900
C	2.66314000	8.29733700	-1.67309900
H	3.11906500	9.08609500	-2.27973200
H	1.57727400	8.36455700	-1.78643400
H	2.91000500	8.48696100	-0.62401600
C	4.70867300	6.85944500	-1.93372100
H	4.98898400	7.03010500	-0.88976300
H	5.10963300	5.88841800	-2.24002900
H	5.18604800	7.63255100	-2.54272900
C	0.21061800	0.13678200	0.71719000
N	-0.24907100	0.21273300	-0.74147400
C	-0.66025200	-1.15088500	-1.24686000

C	-1.44230700	1.14231100	-0.81364600
C	0.85755000	0.76660600	-1.60948100
H	0.43504500	1.16761700	1.00542600
H	-0.66268300	-0.19521400	1.28581000
H	0.19543200	-1.82191700	-1.22105300
H	-1.47049700	-1.54873500	-0.63349900
H	-1.00938800	-1.04264900	-2.27361700
H	-1.79486400	1.17255300	-1.84451100
H	-1.16497000	2.15032600	-0.49861100
H	-2.23161900	0.75665900	-0.16927600
H	1.14160400	1.74819100	-1.22976600
H	1.72377400	0.10518300	-1.58874700
H	0.47759400	0.86061100	-2.62674400
C	1.40919300	-0.76652700	0.98081100
H	2.26874300	-0.48254000	0.36413800
H	1.17044800	-1.80833600	0.73895400
C	1.81643200	-0.68610100	2.46091900
H	2.02322200	0.35925200	2.72759200
H	0.98245200	-1.01284600	3.09741700
C	3.05428900	-1.53576500	2.76644900
H	3.88261500	-1.21149400	2.12205200
H	2.85203900	-2.58304500	2.50164500
C	3.48675700	-1.46016500	4.23408900
H	3.69266400	-0.41375000	4.49404200
H	2.65466200	-1.78113900	4.87701400
C	4.71658000	-2.32470400	4.53501200
H	5.55866300	-1.98416900	3.91579600
H	4.50505900	-3.35762400	4.22674200
C	5.14171200	-2.32036200	6.01046300
H	5.93596100	-3.06369000	6.14949800
H	4.29797500	-2.65431700	6.62922000
C	5.63838300	-0.96068300	6.51093800
H	4.85034100	-0.20167300	6.47860300
H	5.98690600	-1.02685000	7.54586100
H	6.47457800	-0.60165800	5.90021100

[(P_3)-**3**·(**G15**)]$^+$

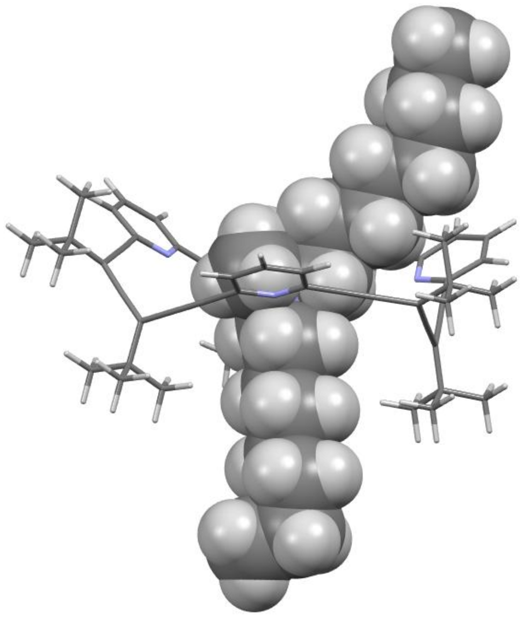

Figure S62. Optimized structure of a complex between (P_3)-**3** and didecyldimethylammonium (**G15**). Alkyl chains were shortened to 8 carbon atoms.

C	-4.07648300	4.88033500	-3.34739300
C	-2.78390900	5.16735300	-2.93770300
C	-2.16194500	4.31152000	-2.01885600
N	-2.76091600	3.21782300	-1.52525700
C	-4.01738700	2.96042300	-1.91678400
C	-4.71294800	3.76242800	-2.82795400
C	-4.66579500	1.80746700	-1.35384400
C	-5.27657500	0.86179000	-0.91275400
C	-6.00613900	-0.25907200	-0.38494500
C	-6.06993900	-2.42432300	-1.87062600
C	-5.00894600	-3.37113100	-1.68474500
C	-4.11913700	-4.17314500	-1.52016500
C	-1.15372800	-7.03491800	-0.92040400
C	-2.20519500	-7.34228300	-1.77182100
C	-3.19679300	-6.39691100	-1.98421700
C	-3.09642800	-5.16080800	-1.33351700
N	-2.07742200	-4.84766200	-0.51986400
C	-1.13140300	-5.77440400	-0.31295400
C	-0.06022400	-5.43531000	0.58310700
C	0.85688200	-5.21394900	1.33926500

C	1.58116500	-4.89169800	3.77095000
C	5.30244500	-5.91883800	1.01310200
C	1.92484700	-4.98416000	2.27350800
C	4.34035800	-4.74588800	1.26910600
C	4.76478300	-3.42067100	0.92083300
C	5.12639400	-2.29959600	0.64773400
C	6.50114000	1.56913000	-0.05740600
C	7.34055700	0.50495600	-0.35727100
C	6.89143400	-0.78851800	-0.13871300
C	5.60044700	-0.97423100	0.37362700
N	4.77423500	0.04604700	0.64518100
C	5.22465400	1.29181800	0.44385100
C	4.33455400	2.37078700	0.77270000
C	3.61125600	3.30492200	1.02838200
C	2.73817400	4.41784500	1.28627700
C	1.54694600	5.47388800	-0.80363200
C	0.25918600	4.98384200	-1.20305900
C	-0.83621800	4.62016800	-1.56536400
C	-6.06067200	-1.34632100	-1.11923500
C	3.14223600	-4.88188600	1.79181400
C	2.12185800	4.94998800	0.25583100
H	-4.58543000	5.52454500	-4.05752800
H	-2.25253000	6.03690500	-3.30720600
H	-5.72745100	3.50347800	-3.10842100
H	-0.36093000	-7.74538600	-0.71623000
H	-2.25421700	-8.31083000	-2.25953100
H	-4.04155800	-6.59732900	-2.63334700
H	6.81357800	2.59751000	-0.19888400
H	8.33673900	0.68286900	-0.75035500
H	7.51730500	-1.64890900	-0.34667200
C	2.58136000	4.90823800	2.73626000
C	2.19488800	6.58082800	-1.65744600
C	-6.67659800	-0.11531300	0.99319900
C	-7.14608000	-2.72222500	-2.92936300
C	2.34510200	6.06585400	-3.09716900
H	1.37864800	5.78463800	-3.52674900
H	2.78121100	6.84573700	-3.73048900
H	3.00293200	5.19098800	-3.13562700

C	1.28765900	7.82034600	-1.63948700
H	0.29212300	7.59603400	-2.03442600
H	1.16952100	8.20897300	-0.62264400
H	1.72397100	8.61278900	-2.25678700
C	3.57304100	6.94880200	-1.10337700
H	3.50692400	7.33694000	-0.08196600
H	4.24767900	6.08671300	-1.09529100
H	4.02592400	7.72567700	-1.72751700
C	2.15152200	3.72557500	3.61720700
H	2.88360700	2.91298600	3.58308600
H	2.05560100	4.05033100	4.65878400
H	1.18196100	3.32808000	3.29714900
C	3.93606200	5.44722200	3.22199200
H	3.85431700	5.78522500	4.26058000
H	4.71273700	4.67757200	3.17878500
H	4.26332900	6.29692000	2.61394300
C	1.52850100	6.01524700	2.81626200
H	1.81176700	6.88481600	2.21491500
H	0.55037700	5.66774300	2.46741500
H	1.41941400	6.34770200	3.85352700
C	5.63754700	-5.95989000	-0.48577300
H	6.10036900	-5.02595300	-0.81930800
H	6.33925500	-6.77554200	-0.69035700
H	4.73759000	-6.12763300	-1.08684400
C	4.65661600	-7.24283500	1.42714900
H	4.41577100	-7.25747100	2.49492500
H	3.73434800	-7.43333300	0.86876800
H	5.34734100	-8.06853500	1.22888100
C	6.58587100	-5.69624600	1.82726300
H	7.07559900	-4.75605400	1.55596600
H	6.37327700	-5.67054400	2.90108300
H	7.29345700	-6.51115000	1.64102700
C	2.84766500	-4.66718600	4.59939500
H	3.55283700	-5.49684100	4.48805200
H	3.36149700	-3.74501600	4.30969600
H	2.58702200	-4.59041200	5.65985800
C	0.90320100	-6.19927200	4.20736500
H	0.63484100	-6.14672000	5.26791700

H	-0.01160100	-6.38784700	3.63714800
H	1.57254800	-7.05480200	4.06986600
C	0.61713100	-3.71372700	3.98101200
H	1.09058700	-2.76671100	3.69795400
H	-0.29772300	-3.83551600	3.39228300
H	0.33210200	-3.64561900	5.03636100
C	-7.39043600	-1.41193400	1.38062900
H	-8.18169700	-1.66361800	0.66747500
H	-6.69605200	-2.25684700	1.42815800
H	-7.85281900	-1.29876400	2.36639300
C	-7.69268200	1.03544300	0.93405500
H	-8.17088200	1.16391100	1.91092900
H	-7.21323300	1.98209500	0.66651800
H	-8.47702400	0.83091300	0.19796600
C	-5.59327700	0.20294100	2.03493400
H	-4.87079400	-0.61758600	2.10968000
H	-5.05201200	1.12046000	1.78242900
H	-6.04914500	0.34126200	3.02114200
C	-6.46183200	-2.86462400	-4.29760100
H	-5.71659100	-3.66600600	-4.29257500
H	-7.20590400	-3.10150200	-5.06547000
H	-5.96060300	-1.93471900	-4.58652000
C	-7.85363200	-4.03526500	-2.56155700
H	-8.60893000	-4.27796300	-3.31657800
H	-7.14872100	-4.87064000	-2.50972600
H	-8.35671900	-3.95505500	-1.59236100
C	-8.17028600	-1.58690600	-2.98761000
H	-8.68908200	-1.46239600	-2.03173700
H	-7.69954200	-0.63261700	-3.24531700
H	-8.92358400	-1.80792600	-3.75040800
C	-0.68529600	-0.06432600	1.20781700
N	-0.30423700	-0.45241800	-0.21007500
C	0.92356200	0.33289500	-0.62890700
C	-0.01819100	-1.92351300	-0.20205200
C	-1.45092100	-0.18531100	-1.13459000
H	-1.50044900	-0.73954300	1.48112500
H	0.17999700	-0.31497200	1.82763500
H	0.63181700	1.38388100	-0.59737600

H	1.67378500	0.16488500	0.14963200
H	0.20823500	-2.25268300	-1.21409800
H	-0.89660700	-2.46414800	0.15235800
H	0.83372600	-2.11660500	0.45095500
H	-2.34016600	-0.67786300	-0.73972300
H	-1.62351900	0.88850900	-1.20862200
H	-1.21748500	-0.59802400	-2.11504000
C	-1.10644200	1.38213200	1.42241000
H	-1.92285800	1.65694600	0.74727600
H	-0.27745000	2.06593100	1.21332800
C	-1.56248300	1.58032100	2.87202900
H	-2.33667600	0.84118700	3.11828000
H	-0.72318300	1.38740500	3.55417700
C	-2.11383400	2.98287400	3.12606300
H	-2.96834700	3.16019100	2.45922400
H	-1.35607200	3.72918400	2.85115600
C	-2.54394900	3.20487800	4.57524200
H	-3.29098600	2.44788200	4.84605000
H	-1.68378500	3.03894100	5.23969000
C	-3.10618100	4.60537700	4.82196700
H	-3.99245800	4.75616400	4.18911700
H	-2.36553600	5.34557300	4.49017400
C	-3.47450300	4.89401700	6.28027900
H	-3.74762500	5.95259800	6.36763100
H	-2.58612600	4.75639600	6.91146100
C	-4.62136300	4.04035100	6.82045800
H	-4.36334500	2.97662100	6.85023600
H	-4.88673900	4.33923400	7.83955400
H	-5.51875100	4.14910100	6.19953700
C	1.49106700	-0.01288100	-1.99579700
H	1.81226800	-1.05967900	-2.03413100
H	0.73963900	0.12683300	-2.78285700
C	2.69924600	0.88125700	-2.28513200
H	2.39410400	1.93565700	-2.23826200
H	3.43495100	0.73677000	-1.48429800
C	3.33599200	0.59259500	-3.64163800
H	3.62800600	-0.46504500	-3.68187800
H	2.58760500	0.73215900	-4.43633500

C	4.54601400	1.48037200	-3.93031700
H	4.24677100	2.53132800	-3.81365800
H	5.31699800	1.30144500	-3.16641200
C	5.15614800	1.29522900	-5.32078800
H	4.38726600	1.47986700	-6.08568400
H	5.91831800	2.07169500	-5.47336800
C	5.79589400	-0.07005300	-5.56787100
H	5.04064800	-0.86200000	-5.48188300
H	6.53434700	-0.26904700	-4.77763900
C	6.47064800	-0.16727800	-6.93271700
H	6.92516900	-1.15144700	-7.08793300
H	5.75064000	-0.00383700	-7.74351400
H	7.26170200	0.58431600	-7.04066800

[(P_3)-**3**·(**G16**)]$^+$

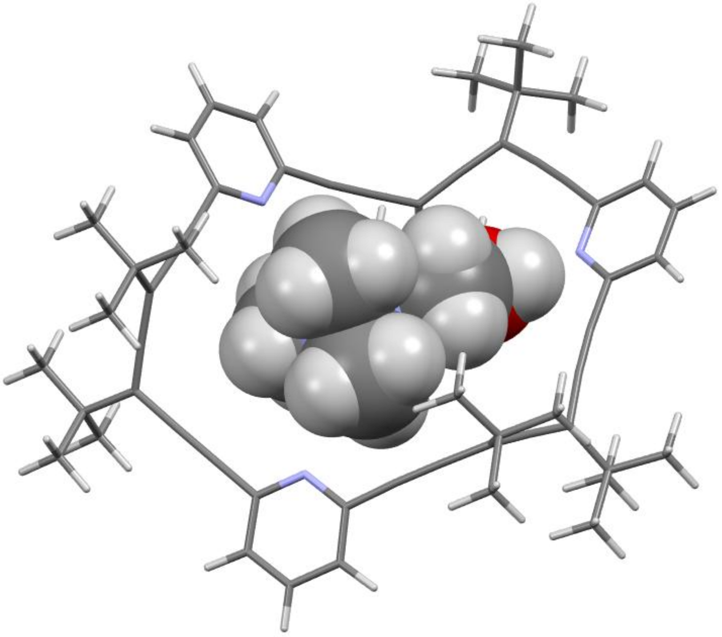

Figure S63. Optimized structure of a complex between (P_3)-**3** and choline ammonium (**G16**).

C	7.11355900	-2.22246400	2.33908900
C	6.10035300	-3.06681200	1.89979400
C	5.05754600	-2.53423700	1.13136200
N	5.01199700	-1.22196800	0.81340300
C	6.00633800	-0.40149600	1.23013200
C	7.07422000	-0.87567100	2.00169100
C	5.92248600	0.96158700	0.82598100
C	5.82464600	2.10143700	0.42782400
C	5.64900100	3.42418700	-0.09498100
C	3.48726700	3.75869100	-1.55552200
C	2.27686000	4.14955600	-0.88933200
C	1.26746100	4.48263200	-0.30527400
C	-2.02766300	5.95194700	1.80196200
C	-0.91034200	6.76462000	1.65392700
C	0.18723800	6.28533700	0.95106000
C	0.14262900	4.98981000	0.41730400
N	-0.93827800	4.19094500	0.56731900
C	-2.01075500	4.66943500	1.24071500
C	-3.15641600	3.82060400	1.35225800
C	-4.16790700	3.15788300	1.43910400
C	-6.00466300	2.18592300	2.91855000

C	-7.34769400	2.00871400	-1.60975400
C	-5.36624600	2.37162000	1.52637400
C	-6.32379300	1.31649100	-0.68788100
C	-5.85505700	0.01278000	-1.05953400
C	-5.45049500	-1.09244700	-1.34972200
C	-4.08127500	-4.95910700	-2.12226900
C	-5.23959300	-4.52945000	-2.75984900
C	-5.71015100	-3.24353200	-2.52337100
C	-4.99578800	-2.41302200	-1.65031600
N	-3.86256200	-2.82205900	-1.03823300
C	-3.41512000	-4.07591800	-1.26286700
C	-2.23019500	-4.49021500	-0.57173200
C	-1.24832800	-4.90855400	0.00380800
C	-0.13316800	-5.46651500	0.71755900
C	2.32485800	-5.24248000	-0.19306800
C	3.20587700	-4.20612200	0.26706600
C	4.01819600	-3.38895500	0.64456300
C	4.58317300	3.63696000	-0.83902500
C	-5.86227800	1.85275200	0.42288600
C	1.09117200	-5.32109600	0.25744500
H	7.92962900	-2.61319500	2.93462100
H	6.10392200	-4.12216500	2.13701000
H	7.85017000	-0.19122700	2.31710500
H	-2.90631800	6.29213000	2.33368600
H	-0.89791200	7.76189300	2.07645900
H	1.07207800	6.89074500	0.80687400
H	-3.69051300	-5.95622500	-2.27619500
H	-5.77299700	-5.19263600	-3.42988800
H	-6.61338400	-2.87861800	-2.99430300
C	-0.45455900	-6.25783400	2.00751300
C	2.91550800	-6.26378300	-1.19469500
C	6.70780000	4.49218100	0.23035800
C	3.42888700	3.47371500	-3.06986500
C	3.37846200	-5.50427700	-2.45349000
H	4.12194200	-4.74119400	-2.20698100
H	3.82978000	-6.20270400	-3.16515200
H	2.53313500	-5.01373100	-2.94593600
C	4.11825400	-6.96803000	-0.53706300

H	4.89115800	-6.24952100	-0.25018700
H	3.80843300	-7.51761500	0.35730800
H	4.56176900	-7.68100500	-1.23910100
C	1.86196200	-7.30808200	-1.58593500
H	1.52234700	-7.87981800	-0.71694300
H	0.98812900	-6.83957000	-2.04857900
H	2.29259200	-8.01072300	-2.30525600
C	-1.17161200	-5.31660300	2.99548900
H	-2.09021000	-4.91110900	2.56182800
H	-1.43726400	-5.86351400	3.90556900
H	-0.52278500	-4.48115500	3.27798800
C	-1.37799500	-7.43867000	1.64997300
H	-1.63423100	-7.99935800	2.55435300
H	-2.30769600	-7.09132400	1.19066900
H	-0.88454400	-8.12292900	0.95286000
C	0.83282400	-6.78858800	2.65106100
H	1.35957600	-7.47704800	1.98352100
H	1.51639300	-5.97364800	2.90776400
H	0.58777900	-7.32980500	3.56954800
C	-6.72711500	2.15695800	-3.01284300
H	-6.44179800	1.18473000	-3.42519800
H	-7.45029900	2.61650200	-3.69372000
H	-5.83701100	2.79308300	-2.98107600
C	-7.71493700	3.39383600	-1.06221400
H	-8.17086300	3.32285600	-0.06995900
H	-6.83460500	4.03947700	-0.98805600
H	-8.43443400	3.87518600	-1.73080200
C	-8.61171200	1.13039400	-1.68909200
H	-8.37813800	0.13218100	-2.07060000
H	-9.07403400	1.02079300	-0.70336300
H	-9.34369300	1.58940900	-2.36098300
C	-7.26547100	1.31765300	2.82071300
H	-8.02111100	1.78321000	2.18082800
H	-7.03766600	0.32768900	2.41423500
H	-7.70011200	1.18581100	3.81581600
C	-6.37154200	3.57180500	3.48386700
H	-6.80424500	3.46543800	4.48354500
H	-5.49017600	4.21518300	3.56148400

H	-7.10608000	4.07141800	2.84499900
C	-4.97836100	1.50128900	3.84278100
H	-4.72306700	0.50409900	3.46905100
H	-4.05987900	2.09102100	3.91997200
H	-5.39567300	1.38993200	4.84838300
C	6.32145600	5.83750400	-0.39767900
H	6.25413900	5.76390200	-1.48745100
H	5.35672200	6.19005800	-0.01997000
H	7.07776000	6.58969600	-0.15524600
C	8.06630500	4.02823800	-0.33080300
H	8.84272100	4.75955400	-0.08493400
H	8.36034600	3.06332400	0.09263900
H	8.02465400	3.92586800	-1.41946200
C	6.80213500	4.64372300	1.76122000
H	5.85015300	4.98464100	2.17979300
H	7.06531800	3.69447000	2.23713200
H	7.57189400	5.37847000	2.01738000
C	2.44455600	2.30991000	-3.30241200
H	1.43391200	2.58750700	-2.98382900
H	2.40715200	2.06288200	-4.36853000
H	2.75786600	1.42424600	-2.74081900
C	2.93876300	4.73721100	-3.80161500
H	2.85285700	4.53842200	-4.87467200
H	1.95786400	5.05376000	-3.43438600
H	3.64015200	5.56604500	-3.66372100
C	4.81580500	3.07739000	-3.59250000
H	5.54541500	3.87735200	-3.43302800
H	5.18295800	2.17409000	-3.09696400
H	4.76074900	2.87944400	-4.66699700
C	-1.00906200	0.65392100	1.33304400
N	-0.78427100	0.26240900	-0.10571000
C	-1.13850700	-1.19152100	-0.31060600
C	-1.68106700	1.12122800	-0.97228000
C	0.65770700	0.53160400	-0.51969700
H	-0.77854100	1.71426400	1.43754400
H	-0.37007400	0.04929500	1.97404500
H	-2.05600700	0.47882800	1.57977400
H	-0.62633000	-1.80189800	0.42972000

H	-2.21648300	-1.34092300	-0.22864600
H	-0.81892800	-1.48788300	-1.30975700
H	-1.49072300	0.87422400	-2.01634800
H	-1.45489800	2.16945800	-0.76712800
H	-2.71947700	0.90281300	-0.72313400
H	0.83378900	1.60230300	-0.39971800
H	0.73020600	0.28404800	-1.57985000
C	1.72390100	-0.26593700	0.22053200
H	1.48334100	-1.33656600	0.24442500
H	1.82586300	0.07968200	1.25802400
O	2.90796500	-0.02007900	-0.53682200
H	3.70558500	-0.45963800	-0.12029000

[(P_3)-**3**·(L-**G17**)]$^+$

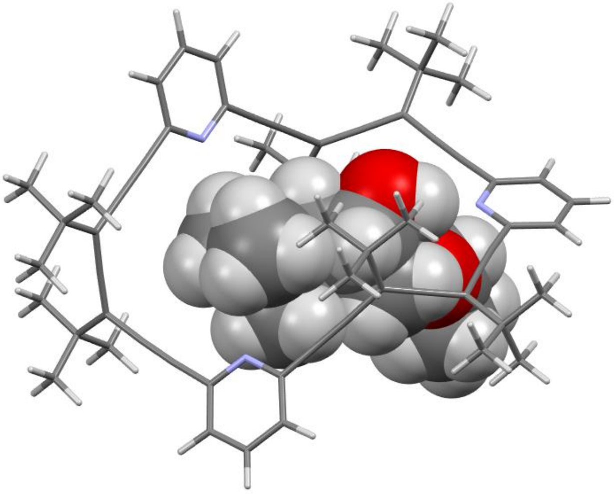

Figure S64. Optimized structure of a complex between (P_3)-**3** and hexyl ester L-carnitine (L-**G17**).

C	7.62876900	1.58206000	-0.37092800
C	6.68846800	2.60106900	-0.45521700
C	5.33351200	2.26540600	-0.56858100
N	4.91873400	0.97926300	-0.59872300
C	5.83392300	-0.01332000	-0.51018100
C	7.20350500	0.26005900	-0.39657400
C	5.36426700	-1.36006600	-0.55055000
C	4.99769500	-2.51346600	-0.60953400
C	4.56391900	-3.87351400	-0.74072900
C	2.18413400	-4.56159000	0.14799200

C	1.06370800	-4.40535900	-0.73609200
C	0.10414100	-4.27017700	-1.46395200
C	-3.26528200	-3.96715300	-3.91697000
C	-2.17178900	-4.69045000	-4.37921100
C	-1.03799000	-4.79787200	-3.58240400
C	-1.02384100	-4.15872800	-2.33573000
N	-2.07487000	-3.43411700	-1.89416500
C	-3.18476700	-3.35189000	-2.66160800
C	-4.29020800	-2.62482000	-2.12081100
C	-5.23770800	-2.04231100	-1.63860600
C	-7.52709700	-0.97726800	-1.98885600
C	-6.78863800	-1.56466300	2.64950000
C	-6.35047300	-1.34607300	-1.06112300
C	-6.24789900	-0.70444800	1.49150800
C	-5.62802800	0.55230400	1.80732700
C	-5.08525100	1.61704600	2.01319900
C	-3.12174400	5.29362100	2.27609300
C	-4.16268200	5.04239300	3.16375700
C	-4.83325100	3.82546300	3.10645800
C	-4.43203400	2.88103300	2.15400700
N	-3.41229800	3.11578700	1.29847700
C	-2.77202600	4.30326800	1.34946900
C	-1.72135200	4.50903000	0.39788700
C	-0.84998400	4.70114800	-0.42326200
C	0.13551600	4.92705900	-1.44384100
C	2.65403700	5.32347900	-0.78884600
C	3.55314700	4.20958300	-0.71200000
C	4.34328700	3.29190300	-0.64827400
C	3.38143700	-4.22301800	-0.28016800
C	-6.31852900	-1.04881600	0.22144300
C	1.39291500	5.12226100	-1.10885600
H	8.68286500	1.81638600	-0.28577100
H	6.98172300	3.64214200	-0.43684300
H	7.90663900	-0.55963900	-0.33333700
H	-4.17043000	-3.87777800	-4.50302100
H	-2.20708300	-5.17508100	-5.34734300
H	-0.17556700	-5.36678700	-3.90329900
H	-2.58691200	6.23414000	2.28748700

H	-4.45342300	5.79200500	3.88962900
H	-5.65438900	3.60243300	3.77486500
C	-0.35652900	4.93933600	-2.90973400
C	3.23594500	6.72447700	-0.49500500
C	5.51940000	-4.85682500	-1.45201300
C	1.90962800	-5.14297900	1.55161100
C	3.81199800	6.72932200	0.93462500
H	4.58730600	5.96707700	1.05393600
H	4.25710700	7.70488000	1.15385500
H	3.02662400	6.53890000	1.67266000
C	4.35660600	7.01795100	-1.51121200
H	5.14947100	6.26647300	-1.45636100
H	3.96532000	7.02817200	-2.53308500
H	4.80030700	7.99707000	-1.30544400
C	2.14447200	7.79658800	-0.60936000
H	1.72303700	7.83242400	-1.61852100
H	1.32798900	7.61133700	0.09510000
H	2.57012200	8.77915300	-0.38620600
C	-1.02484300	3.58584300	-3.22035500
H	-1.87116000	3.40096300	-2.55180400
H	-1.39906200	3.58215300	-4.24882500
H	-0.30685100	2.76486500	-3.11883600
C	-1.38339900	6.07655800	-3.07552100
H	-1.76163700	6.09158300	-4.10254700
H	-2.23402500	5.94547300	-2.40066500
H	-0.92579600	7.04832500	-2.86653300
C	0.81853600	5.16039300	-3.87102600
H	1.30671400	6.12273100	-3.69048000
H	1.57088200	4.37228000	-3.77119000
H	0.45488500	5.15555200	-4.90268100
C	-5.62745700	-1.89005400	3.60949600
H	-5.16509100	-0.97686600	3.99633600
H	-5.99713400	-2.47014200	4.46065700
H	-4.85775600	-2.48233900	3.10372500
C	-7.39192000	-2.86882200	2.11279000
H	-8.22930600	-2.67119700	1.43681700
H	-6.64744400	-3.45865300	1.56954800
H	-7.76469600	-3.47326300	2.94477100

C	-7.87043500	-0.76022300	3.39685700
H	-7.46975600	0.18291800	3.78012600
H	-8.71273600	-0.53189800	2.73680800
H	-8.24807800	-1.33977200	4.24504300
C	-8.60821000	-0.21434200	-1.21250500
H	-9.01510600	-0.81923400	-0.39639000
H	-8.21431900	0.71343500	-0.78668800
H	-9.43154900	0.04270000	-1.88514800
C	-8.12327500	-2.27559300	-2.56707600
H	-8.94224400	-2.03720600	-3.25297000
H	-7.37101800	-2.84611500	-3.11941500
H	-8.51925200	-2.91252900	-1.77025000
C	-6.99376800	-0.09274400	-3.13280400
H	-6.57792800	0.84131400	-2.74235500
H	-6.21273800	-0.60659000	-3.70062000
H	-7.80725600	0.15695100	-3.82118400
C	4.89088800	-6.25356300	-1.53902700
H	4.69042900	-6.66499400	-0.54500200
H	3.94905700	-6.23227500	-2.09529400
H	5.57583600	-6.93326900	-2.05432800
C	6.83552200	-4.93164700	-0.65383800
H	7.54143000	-5.59957200	-1.15746800
H	7.30228800	-3.94604300	-0.56736000
H	6.65944600	-5.31839700	0.35477200
C	5.80069100	-4.33145300	-2.87348600
H	4.87803700	-4.27875700	-3.45927900
H	6.24560200	-3.33276500	-2.84732000
H	6.49635800	-5.00186800	-3.38810500
C	0.88904900	-4.24920700	2.28277600
H	-0.03717800	-4.16552800	1.70503100
H	0.64137300	-4.68971500	3.25424300
H	1.28960400	-3.24570000	2.45733600
C	1.32952500	-6.56180000	1.38334600
H	1.10745800	-6.99376700	2.36440300
H	0.40378000	-6.54416400	0.80109200
H	2.04241500	-7.21766600	0.87417600
C	3.20638900	-5.21495500	2.36781400
H	3.94469900	-5.86512400	1.88887300

H	3.65513600	-4.22435500	2.48911700
H	2.99405700	-5.61833100	3.36226300
C	-1.82589400	0.69035500	-0.53708700
N	-1.14169300	-0.10642700	0.54826100
C	-1.16861600	0.66779100	1.84237500
C	-1.90995300	-1.39624500	0.75926300
C	0.27809100	-0.48176500	0.13361100
H	-1.99154100	0.03420300	-1.39082800
H	-1.19454900	1.53076700	-0.81763600
H	-2.76508300	1.08353900	-0.14820100
H	-0.84780000	1.68966200	1.65380500
H	-2.19560400	0.68960400	2.20239400
H	-0.50928800	0.16905300	2.55271100
H	-1.47141900	-1.91591800	1.61133500
H	-1.85141300	-2.02266900	-0.13569400
H	-2.95004500	-1.14135900	0.96526600
H	0.20146500	-0.92725200	-0.85856900
H	0.61684700	-1.24405300	0.83258700
C	1.33711300	0.62865100	0.03507500
H	0.93450000	1.51152300	-0.48424300
O	2.32886200	-0.02440000	-0.76117500
H	3.22681700	0.42007300	-0.74346700
C	1.96512200	1.13403800	1.35396300
H	1.24702500	1.72305300	1.92719900
H	2.79476700	1.79337300	1.09817400
C	2.46222500	0.02349900	2.23107900
O	1.72112200	-0.81105000	2.77347800
O	3.80326500	0.02262700	2.37396600
C	4.42856900	-1.03022900	3.19683700
H	5.43593500	-1.11106800	2.78943600
H	3.88532500	-1.96246600	3.03033600
C	4.43253100	-0.64281800	4.66604200
H	3.39795000	-0.54105200	5.01006000
H	4.91191900	0.33593600	4.77488500
C	5.16459000	-1.68908100	5.51132800
H	5.16102200	-1.40620700	6.56683500
H	4.68760300	-2.67134900	5.42910900
H	6.20899200	-1.79462000	5.19922300

[(P_3)-**3**·(D-**G17**)]$^+$

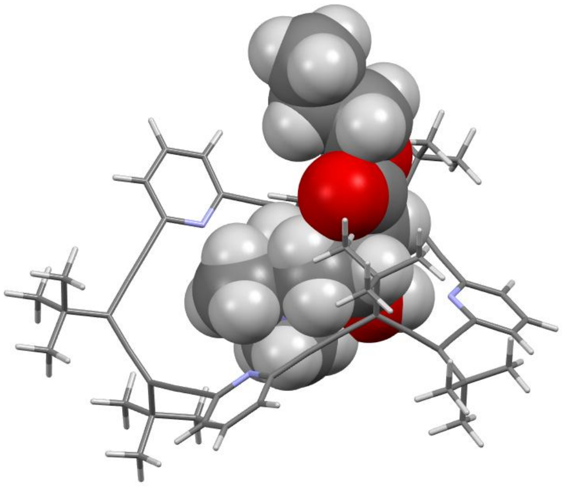

Figure S65. Optimized structure of a complex between (P_3)-**3** and hexyl ester D-carnitine (D-**G17**).

C	6.64607300	0.92085900	-3.59786500
C	5.87807500	2.00195000	-3.18057100
C	4.79358100	1.77276500	-2.32638700
N	4.46874900	0.52646900	-1.91074200
C	5.24097500	-0.52068300	-2.28389700
C	6.33797200	-0.35334600	-3.13890400
C	4.92974000	-1.79947600	-1.73625300
C	4.74343500	-2.88779200	-1.23593200
C	4.54510100	-4.18323700	-0.65839800
C	2.86422600	-4.49007500	1.34419600
C	1.50257500	-4.78147800	0.98749700
C	0.36798300	-5.03362300	0.64376000
C	-3.41025500	-6.14572300	-0.74117500
C	-2.53825500	-7.13968700	-0.31487800
C	-1.28361400	-6.78063600	0.16122000
C	-0.93297100	-5.42475000	0.19577200
N	-1.77984700	-4.45268800	-0.20978700
C	-3.00020600	-4.80839000	-0.67171800
C	-3.88548900	-3.76136300	-1.07839600
C	-4.68282600	-2.90790000	-1.40140000
C	-5.90169300	-1.61197200	-3.23446600

C	-7.96847300	-0.88570700	0.98803900
C	-5.61922500	-1.87280300	-1.74229800
C	-6.66229200	-0.51708600	0.25787800
C	-5.93428600	0.63126600	0.71265600
C	-5.30411100	1.60150600	1.07404800
C	-3.21419400	5.06150600	2.10952800
C	-4.33710000	4.71990500	2.85448000
C	-5.04201000	3.56742000	2.53056600
C	-4.58983300	2.77402700	1.46721700
N	-3.48644100	3.08972000	0.75315300
C	-2.81946100	4.22307700	1.05863600
C	-1.69892400	4.57216900	0.23728600
C	-0.77712200	4.94693200	-0.45496200
C	0.26316700	5.45204900	-1.30621900
C	2.78653200	4.94903200	-0.75905800
C	3.43466700	3.81311600	-1.34952500
C	4.02120400	2.86031300	-1.81512800
C	3.72694900	-4.31928600	0.36470400
C	-6.17595300	-1.19631200	-0.76029000
C	1.52197300	5.19164900	-1.02952100
H	7.48702800	1.07299800	-4.26336200
H	6.10437200	3.01107300	-3.49730400
H	6.93046600	-1.21314500	-3.42121300
H	-4.39553500	-6.38387900	-1.11936000
H	-2.83237800	-8.18140700	-0.35440300
H	-0.57475200	-7.52498000	0.49882400
H	-2.65046800	5.96088100	2.31971600
H	-4.66541300	5.35137200	3.67115300
H	-5.93070400	3.27824400	3.07607200
C	-0.17292300	6.30982000	-2.51613700
C	3.64035500	5.84159400	0.16966700
C	5.31664900	-5.36560700	-1.28569400
C	3.23648300	-4.46049900	2.83999400
C	4.25061200	4.96171900	1.27897300
H	4.91629500	4.20209500	0.85886700
H	4.83725500	5.58374800	1.96274200
H	3.47492900	4.44937100	1.85679000
C	4.76635800	6.48697900	-0.66164700

H	5.38892300	5.72666500	-1.14248100
H	4.35462600	7.13659400	-1.44020100
H	5.40924500	7.09290400	-0.01516000
C	2.77389400	6.93758200	0.80338500
H	2.32784600	7.58558900	0.04308600
H	1.96465000	6.50746400	1.40134100
H	3.39007500	7.55974000	1.45906400
C	-1.09060400	5.45859300	-3.41548500
H	-1.96868900	5.10705800	-2.86651100
H	-1.43589900	6.05522000	-4.26571500
H	-0.55477300	4.58719800	-3.80465500
C	-0.94335200	7.54085200	-2.00066200
H	-1.27944900	8.15079100	-2.84514600
H	-1.82362800	7.24495600	-1.42319500
H	-0.30626900	8.16234200	-1.36356800
C	1.05036100	6.76818000	-3.32074500
H	1.71838700	7.38830300	-2.71517100
H	1.62339600	5.91493300	-3.69600100
H	0.72323800	7.36236200	-4.17888500
C	-7.64900800	-1.13566000	2.47493900
H	-7.19278400	-0.25468900	2.93577400
H	-8.56890000	-1.36805300	3.02058900
H	-6.96133500	-1.97929100	2.59008500
C	-8.58747000	-2.14917500	0.37675100
H	-8.83574300	-2.00052600	-0.67856800
H	-7.90601200	-3.00208800	0.45050600
H	-9.50914600	-2.40260000	0.90850800
C	-8.95531700	0.29092600	0.85707500
H	-8.53437400	1.20994400	1.27519100
H	-9.20542500	0.47579800	-0.19210600
H	-9.88143000	0.06411100	1.39458700
C	-6.92942800	-0.48479800	-3.39691800
H	-7.88355400	-0.74692800	-2.92965200
H	-6.57452500	0.44713100	-2.94678100
H	-7.11232500	-0.30230700	-4.45988600
C	-6.44472600	-2.90601100	-3.87078800
H	-6.63023600	-2.74882800	-4.93795200
H	-5.73180400	-3.72916300	-3.76642900

H	-7.38647100	-3.20534800	-3.40081500
C	-4.58104300	-1.20911700	-3.92077800
H	-4.19233200	-0.27680800	-3.49728100
H	-3.82183800	-1.98889900	-3.80601500
H	-4.74740100	-1.05193600	-4.99111500
C	4.98537000	-6.67372400	-0.55595600
H	5.27848800	-6.62823400	0.49744300
H	3.91546300	-6.89713200	-0.60386700
H	5.52745100	-7.50168900	-1.02190400
C	6.82786000	-5.08388400	-1.17861300
H	7.39365600	-5.89805100	-1.64230200
H	7.09516900	-4.15210700	-1.68554000
H	7.13778600	-5.00532100	-0.13199200
C	4.91105000	-5.48691900	-2.76784600
H	3.84269700	-5.70354800	-2.86364400
H	5.12352100	-4.56418500	-3.31539900
H	5.46917800	-6.30049700	-3.24173800
C	2.27910200	-3.51592300	3.59002000
H	1.23842700	-3.83135800	3.46722100
H	2.51069200	-3.53229600	4.65991500
H	2.37120400	-2.48210200	3.24516000
C	3.08988300	-5.89210000	3.39590200
H	3.32427900	-5.90315800	4.46508100
H	2.06797600	-6.26181600	3.26836400
H	3.77124700	-6.58193300	2.88850800
C	4.68218300	-3.98026300	3.02109300
H	5.39173700	-4.64278000	2.51612800
H	4.81618800	-2.96957500	2.62339100
H	4.93422000	-3.96330800	4.08537300
C	-0.77463700	-1.05601100	-1.42472800
N	-0.74663300	-0.45229900	-0.03281300
C	-0.86104600	1.04821100	-0.12961500
C	-1.93444800	-0.99237800	0.73217200
C	0.51869200	-0.89556400	0.70586400
H	-0.85506400	-2.13868400	-1.31827600
H	0.14895900	-0.76936800	-1.92171000
H	-1.65323800	-0.66965400	-1.94216800
H	-0.07131100	1.40691000	-0.78556600

H	-1.84655800	1.31283100	-0.51206900
H	-0.76454800	1.47430700	0.86812700
H	-1.93467900	-0.56166000	1.73311900
H	-1.85846900	-2.08017100	0.76806100
H	-2.84548500	-0.70475500	0.20775400
H	0.60013600	-1.96475400	0.50061500
H	0.33313100	-0.76930500	1.77273000
C	1.84351200	-0.20044200	0.35089100
C	2.16087200	1.10877300	1.08990000
H	1.44289300	1.89852000	0.85613800
H	3.12092500	1.48139500	0.71700800
C	2.26524200	0.96882700	2.57866700
O	1.99814300	-0.05340000	3.22119000
O	2.69409200	2.11426300	3.16319800
C	2.85976300	2.13693900	4.63008200
H	3.61641300	2.90400400	4.79345400
H	3.23825400	1.16307600	4.94755500
C	1.54936100	2.47493700	5.32097200
H	0.81769500	1.69069100	5.09920200
H	1.16200800	3.41271800	4.90790000
C	1.73833800	2.60073900	6.83543300
H	0.79311400	2.84708900	7.32551400
H	2.10339600	1.66446100	7.26991600
H	2.45644300	3.38944500	7.08313800
H	2.61170000	-0.92905000	0.64544200
O	1.89567400	0.03018800	-1.06218300
H	2.83648500	0.21734500	-1.36481400

6. ECD Measurements of Thin Films

Samples were prepared by dropwise depositing ~ 150 µL of a 2.0×10^{-3} M solution of (M_2)-**2** (Et_3N) onto a quartz plate (1 mm thick), followed by slow evaporation. Then, another quartz plate was placed on top to fix the sample, resulting in a total optical path thickness of 2 mm. ECD measurements were taken from both sides of the plate (Face A and Face B). For each side, the plate was rotated 90 degrees four times, and measurements were taken at each rotation (**Figure S66**, left). Afterwards, the measurements from the different angles on the same side were averaged. Linear Dichroism (LD) measurements were also performed to confirm that the LD signal was negligible.

The experimentally obtained ECD of the thin films of (M_2)-**2** was compared with the computationally calculated ECD [# td=(nstates=30) cam-b3lyp/6-31g(d,p) integral=ultrafine] of three (M_2)-**2** forming a helix (**Figure S67**) directly obtained from the crystallographic coordinates previously determined by the research group[1] (**Figure S66**, right).

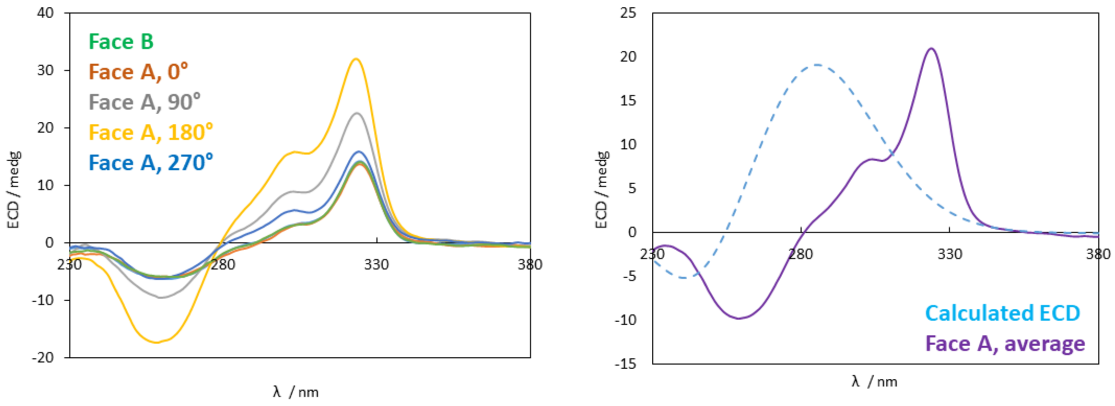

Figure S66. Left: ECD spectrum obtained for the thin films of (M_2)-**2** prepared under the conditions described above. Right: Comparison between the averaged ECD spectrum obtained for the thin film and the computationally calculated spectrum for structure shown in **Figure S67** (# td=(nstates=30) cam-b3lyp/6-31g(d,p) integral=ultrafine).

[1] The crystallographic information is available in CCDC 2280778

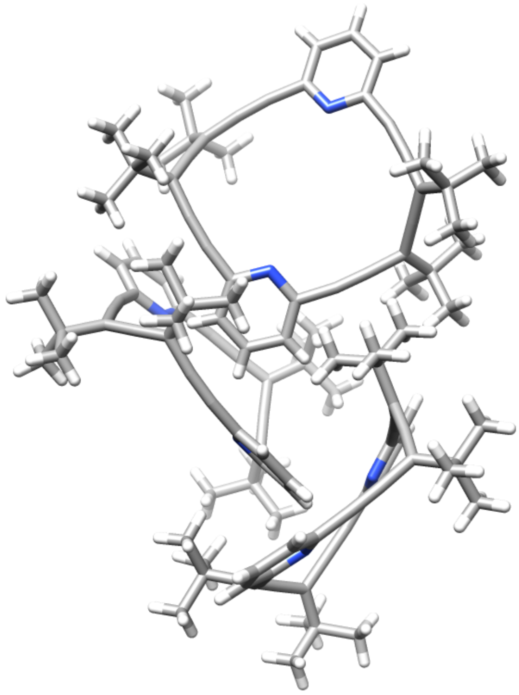

Figure S67. Helix formed by three (M_2)-**2** macrocycles, obtained directly from the crystallographic coordinates.

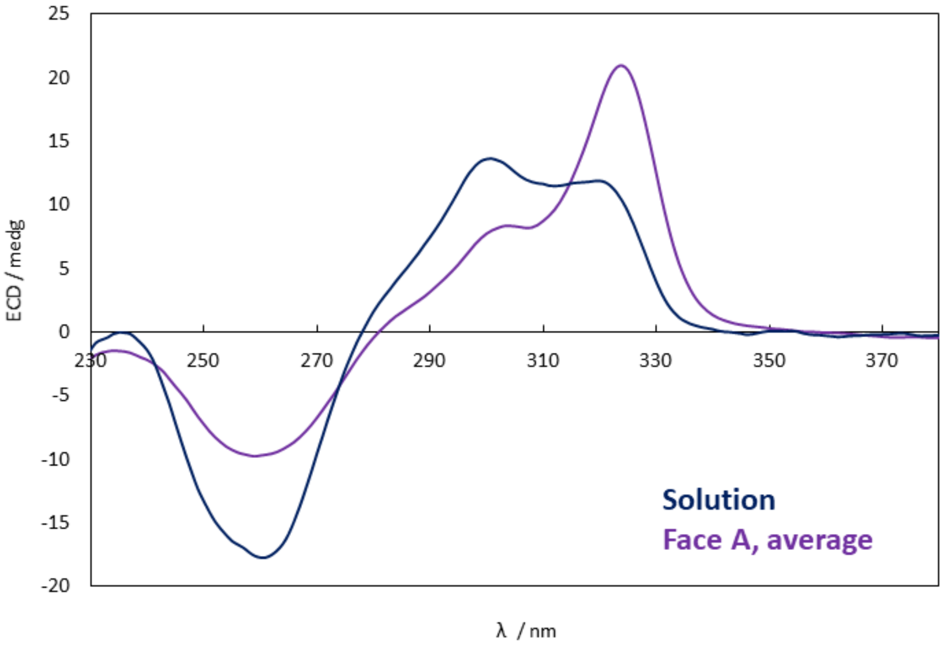

Figure S68. Comparison between the ECD spectrum obtained for the thin films of (M_2)-**2** and the ECD spectrum of (M_2)-**2** in solution (CH_3CN, 1.5 x 10^{-5} M).

8. Thermodynamic Analysis of Host–Guest Complexation

To gain deeper insight into the thermodynamic contributions to the binding, we selected two representative host–guest pairs — ((P_2)-**2** with catechol (**G1**)) and ((M_3)-**3** with the L-carnitine derivative L-**G17**) — as these show the highest association constants for each allenophane. Titration experiments were performed at different temperatures to determine the corresponding association constants. By applying the van't Hoff equation (**1**) and constructing the corresponding van't Hoff plots, the enthalpic (ΔH) and entropic (ΔS) contributions to the binding processes were obtained. The resulting thermodynamic parameters are shown below.

The titrations were carried out following the procedure previously described in the General Methods section. For the different temperatures at which the titrations were performed, a Peltier temperature controller integrated into the Jasco J-815 spectropolarimeter was used, allowing the temperature to remain stable and constant throughout the entire titration process.

$$ln(K) = -\frac{\Delta H}{R}\frac{1}{T} + \frac{\Delta S}{R} \quad (\mathbf{1})$$

Table S7. Association constants obtained at different temperatures for the titrations between (P_2)-**2** and **G1**, and between (M_3)-**3** and L-**G17**.

Entry	Host	Guest	(H:G)	K (M^{-1})	T (K)
1	(P_2)-**2**	**G1**[a]	1:1	K_1 = 1917 ± 491	283
2	(P_2)-**2**	**G1**[a]	1:1	K_1 = 1484 ± 380	293
3	(P_2)-**2**	**G1**[a]	1:1	K_1 = 1261 ± 321	298
4	(P_2)-**2**	**G1**[a]	1:1	K_1 = 1015 ± 260	313
5	(M_3)-**3**	L-**G17**[b]	1:1	K_1 = 4592 ± 550	283
6	(M_3)-**3**	L-**G17**[b]	1:1	K_1 = 4422 ± 530	293
7	(M_3)-**3**	L-**G17**[b]	1:1	K_1 = 4320 ± 648	298
8	(M_3)-**3**	L-**G17**[b]	1:1	K_1 = 4100 ± 388	313

[a] (1.5 × 10^{-5} M, $CHCl_3$). [b] (1.5 × 10^{-5} M, $CHCl_3$/EtOH 5%).

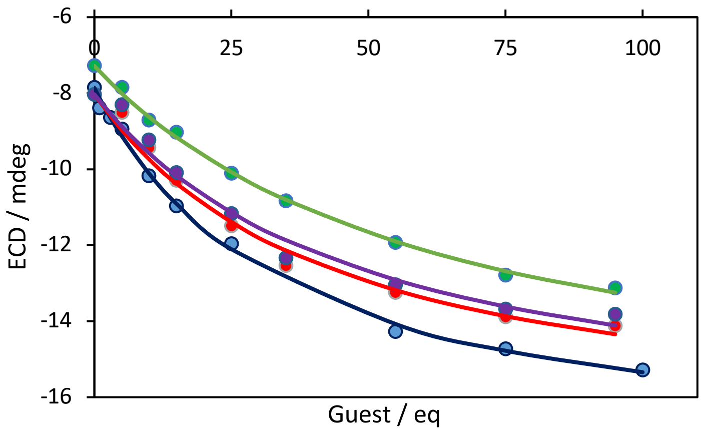

Figure S69. Mathematical fitting of experimental data for the ECD titration of (P_2)-**2** (1.5 × 10^{-5} M, $CHCl_3$) with catechol (**G1**) at different temperatures. Experimental data are shown as points, and the mathematical fits as lines. Wavelength: 307 nm. Blue (10 °C), red (20 °C), purple (25 °C), and green (40 °C).

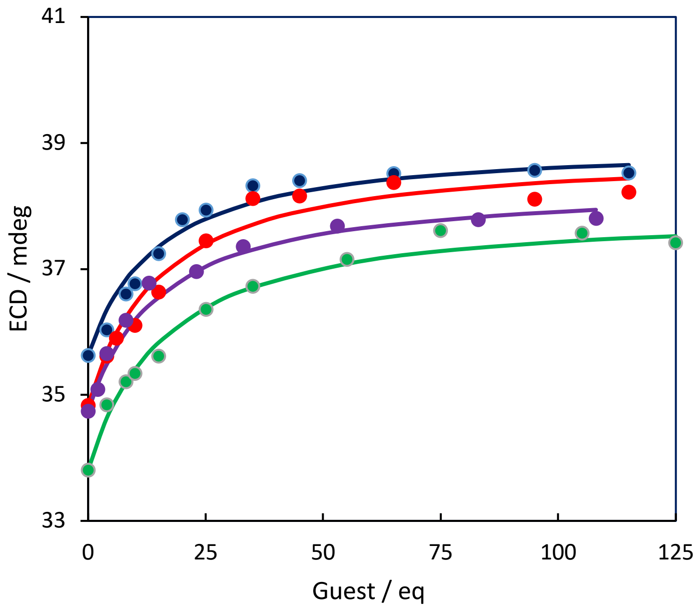

Figure S70. Mathematical fitting of experimental data for the ECD titration of (*M*$_3$)-**3** (1.5 × 10^{-5} M, $CHCl_3$/EtOH 5%) with L-**G17** at different temperatures. Experimental data are shown as points, and the mathematical fits as lines. Wavelength: 331 nm. Blue (10 °C), red (20 °C), purple (25 °C), and green (40 °C).

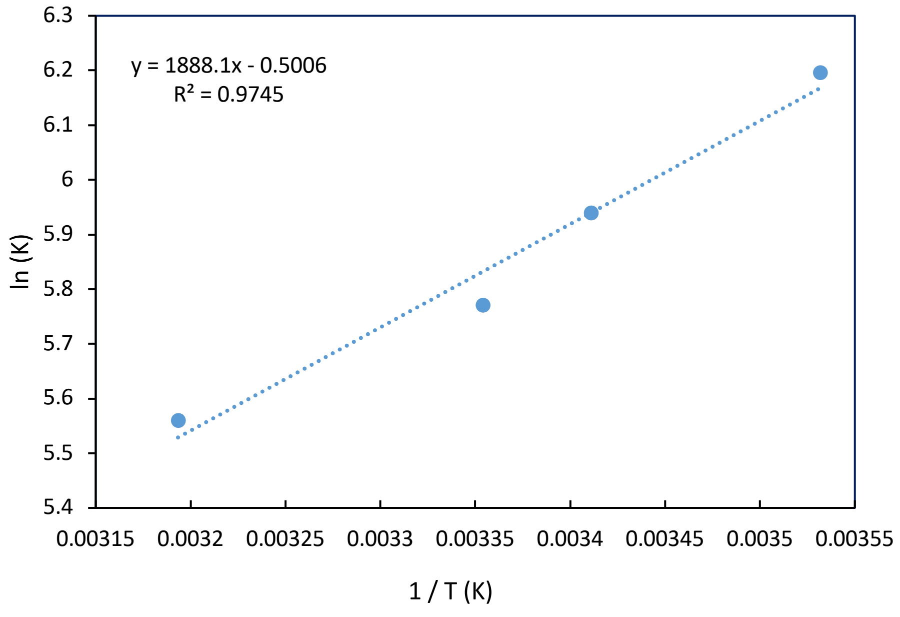

Figure S71. Van't Hoff plot for the binding of catechol (**G1**) by (*P*$_2$)-**2**.

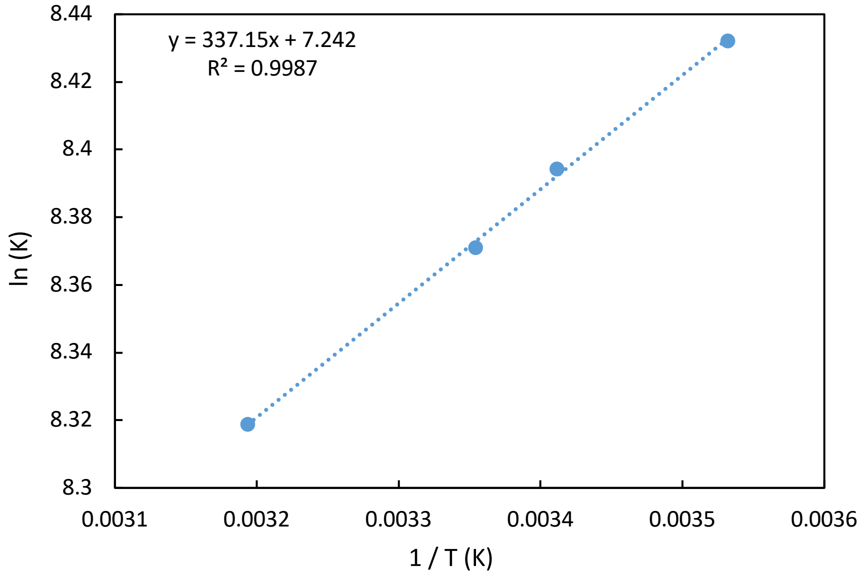

Figure S72. Van't Hoff plot for the binding of L-**G17** by (M_3)-**3**.

Table S8. Thermodynamic parameters obtained from the van't Hoff plots shown in **Figures S70** and **S71**.

**System**	(P_2)-**2** · **G1**	(M_3)-**3** · L-**G17**
**Dominant interaction**	Hydrogen bonding and van der Waals contacts	Electrostatic interactions and desolvation effects
**Host structure**	Rigid, preorganized, small cavity	More flexible host, larger cavity
**ΔH° (kJ mol⁻¹)**	−15.6	−2.8
**ΔS° (J mol⁻¹ K⁻¹)**	+7.9	+60
**ΔG° (298 K, kJ mol⁻¹)**	≈ −18	≈ −20
**Thermodynamic control**	**Enthalpy-driven**	**Entropy-driven**

The thermodynamic data reveal distinct driving forces for the two complexation processes. For system (P_2)-**2·G1**, the negative enthalpy and small positive entropy indicate an enthalpy-controlled association, dominated by directional hydrogen bonding and van der Waals contacts within a rigid, preorganized host featuring a small cavity. In contrast, system (M_3)-**3**·L-**G17** exhibits a much smaller enthalpic contribution but a markedly positive entropy, reflecting an entropy-driven complexation process. This behavior is consistent with electrostatic interactions accompanied by substantial solvent release upon binding within a more flexible, larger cavity. Although the direct interactions in (M_3)-**3**·L-**G17** are weaker (smaller |ΔH°|), the entropic gain (TΔS) compensates for this, resulting in a comparable overall stability (ΔG ≈ -18 /−20 kJ·mol^{-1}).

9. References

(1) Perrin, D.; Armarego, W. *Purification of Laboratory Chemicals*; Pergamosn Press, 1998.

(2) APEX4 v.2022.1-1 (Bruker AXS Inc., 2022).

(3) SAINT v. 8.40B (Bruker AXS Inc., 2019).

(4) Krause, L.; Herbst-Irmer, R.; Sheldrick, G. M.; Stalke, D. Comparison of Silver and Molybdenum Microfocus X-Ray Sources for Single-Crystal Structure Determination. *J. Appl. Crystallogr.* **2015**, *48* , 3–10.

(5) Sheldrick, G. M. SHELXT - Integrated Space-Group and Crystal-Structure Determination. *Acta Crystallogr. Sect. A Found. Crystallogr.* **2015**, *71* (1), 3–8.

(6) Sheldrick, G. M. Crystal Structure Refinement with SHELXL. *Acta Crystallogr. Sect. C Struct. Chem.* **2015**, *71* , 3–8.

(7) Dolomanov, O. V.; Bourhis, L. J.; Gildea, R. J.; Howard, J. A. K.; Puschmann, H. OLEX2: A Complete Structure Solution, Refinement and Analysis Program. *J. Appl. Crystallogr.* **2009**, *42* , 339–341.

(8) Spek, A. L. Single-Crystal Structure Validation with the Program PLATON. *J. Appl. Crystallogr.* **2003**, *36* , 7–13.

(9) Brynn Hibbert, D.; Thordarson, P. The Death of the Job Plot, Transparency, Open Science and Online Tools, Uncertainty Estimation Methods and Other Developments in Supramolecular Chemistry Data Analysis. *Chem. Commun.* **2016**, *52* , 12792–12805.

(10) Http://app.supramolecular.Org/Bindfit.

(11) Gaussian 09, Revision A.02, M. J. Frisch, G. W. Trucks, H. B. Schlegel, G. E. Scuseria, M. A. Robb, J. R. Cheeseman, G. Scalmani, V. Barone, G. A. Petersson, H. Nakatsuji, X. Li, M. Caricato, A. Marenich, J. Bloino, B. G. Janesko, R. Gomperts, B. Mennucci, 2016. Gaussian 09.

(12) Yanai, T.; Tew, D. P.; Handy, N. C. A New Hybrid Exchange–Correlation Functional Using the Coulomb-Attenuating Method (CAM-B3LYP). *Chem. Phys. Lett.* **2004**, *393* , 51–57.

(13) Marenich, A. V.; Cramer, C. J.; Truhlar, D. G. Universal Solvation Model Based on Solute Electron Density and on a Continuum Model of the Solvent Defined by the Bulk Dielectric Constant and Atomic Surface Tensions. *J. Phys. Chem. B* **2009**, *113* , 6378–6396.

(14) Padula, D.; Lahoz, I. R.; Díaz, C.; Hernández, F. E.; Di Bari, L.; Rizzo, A.; Santoro, F.; Cid, M. M. A Combined Experimental–Computational Investigation to Uncover the Puzzling (Chiro-)Optical Response of Pyridocyclophanes: One- and Two-Photon Spectra. *Chem. – A Eur. J.* **2015**, *21* , 12136–12147.

(15) Chai, J.-D.; Head-Gordon, M. Long-Range Corrected Hybrid Density Functionals with Damped Atom–Atom Dispersion Corrections. *Phys. Chem. Chem. Phys.* **2008**, *10*, 6615–.

(16) Álvarez-García, J.; Rubio-Pisabarro, V.; García-Río, L.; Cid, M.M. Deciphering the Degree of Proton-Transfer in Pyrido-Cyclophanes by Chiroptical Outcomes in Non-Aqueous Solvents. *Org. Chem. Front.* **2023**, *10* (21), 5435–5442.
